# Epithelium stratifies via nucleation and growth induced by foam-geometric instability

**DOI:** 10.1038/s41467-026-78964-1

**Published:** 2026-11-13

**Authors:** Shuya Fukamachi, Razib Datta, Duligengaowa Wuergezhen, Takehiko Ichikawa, Rei Yagasaki, Phoebe Leeaw, Makiko Arai, Aki Teranishi, Shuhei A. Horiguchi, Kohei Omachi, Aiko Sada, Itaru Imayoshi, Kentaro Kajiwara, Tetsuya Hiraiwa, Masanobu Oshima, Takeshi Fukuma, Hironobu Fujiwara, Satoru Okuda

**Affiliations:** 1 Graduate School of Frontier Science Initiative, Kanazawa University, Kanazawa, Japan; 2 Laboratory for Tissue Microenvironment, RIKEN Center for Biosystems Dynamics Research (BDR), Kobe, Japan; 3 Nano Life Science Institute, Kanazawa University, Kanazawa, Japan; 4 Division of Skin Regeneration and Aging, Medical Institute of Bioregulation, Kyushu University, Fukuoka, Japan; 5 Graduate School of Biostudies, Kyoto University, Kyoto, Japan; 6 Institute for Life and Medical Sciences, Kyoto University, Kyoto, Japan; 7 Research Institute for Microbial Diseases, Osaka University, Osaka, Japan; 8 Institute of Physics, Academia Sinica, Taipei, Taiwan; 9 Cancer Research Institute, Kanazawa University, Kanazawa, Japan; 10 Sapiens Life Sciences, Evolution and Medicine Research Center, Kanazawa, Japan

**Keywords:** Biophysics, Morphogenesis, Cell biology

## Abstract

Epithelial stratification, the transition from a monolayer to a multilayer structure, occurs across a wide range of phenomena. Recent studies have identified several cell behaviors as triggers, including junctional tension, cell density and geometry, and topological defects. However, how these factors drive stratification throughout the entire epithelium remains poorly understood. Here, we show that epithelial stratification proceeds through a nucleation-and-growth mechanism. Combining cell culture with three-dimensional vertex modeling, we demonstrate that multilayer origins emerge in a dispersed manner and expand across the epithelium through disordered intermediate states. This transition is induced by a mechanical instability inherent in the foam-like geometry of the epithelium. Moreover, the nucleation-growth concept applies to embryonic skin development and intestinal cancer transformation. These findings conceptualize epithelial stratification as a form of structural transition governed by foam mechanics, offering a physical perspective on various epithelial developments.

## Abstract (web summary)

Researchers show that epithelial stratification, the shift from monolayer to multilayers, occurs via a nucleation-and-growth process. Combining cell cultures and 3D modeling, this study reveals how local cell delaminations drive tissue-wide changes.

## Introduction

Many epithelia develop multilayer sheet structures^1^, such as those found in the skin^2^, hair follicles^3^, cornea^4^, esophagus^5^, stomach^6^, intestine^7^, bronchus^8^, and brain^9^. These multilayer structures typically evolve from a monolayer through a stratification process, a key phenomenon in embryogenesis. Defects in this process lead to fatal diseases. For instance, during embryogenesis, mouse skin, which normally forms a multilayer, remains a monolayer when the p63 gene is defective, resulting in postnatal death due to dehydration^10–12^. Moreover, during carcinogenesis, abnormal cells undergo stratification to form tumors on epithelial sheets^13^. Consequently, epithelial stratification has been widely studied in various biological contexts. However, although key molecules and cell behaviors have been identified, the mechanism of collective cell dynamics inducing the stratification of the entire epithelium remains poorly understood. Thus, it is crucial to develop a comprehensive understanding of the stratification process, particularly how individual cell delaminations lead to the stratification of the overall epithelium.

During epithelial stratification, cells typically delaminate from either the apical or basal side and accumulate vertically within the epithelial sheet. Several mechanisms have been proposed to explain delamination of individual cells. For example, in skin and brain development, cell delamination often results directly from vertically oriented cell divisions^14–16^, whereas recent pioneering work has shown that cells stratify even without the regulation of cell division orientation^15^. Additionally, cells that lose their basal side during mitosis can regain it by elongating their protrusions^17,18^. These findings suggest that transient vertically oriented cell divisions may not be sufficient; instead, a balance of mechanical forces among cells is required to form a stable, stratified structure.

From a physical viewpoint, apical junctional forces, such as increased actomyosin contractility and reduced cadherin-mediated adhesion, are well-known contributors to cell delamination^19–24^. Moreover, an increase in cell density within the epithelial plane can drive cells out of the layer^25–27^. Recent studies have also revealed the impact of cell geometry on this process^28–31^; particularly, topological defects trigger initiation of the stratification process^32,33^. These findings underscore the critical role of mechanical forces in epithelial stratification. However, current understanding is largely limited to the initiation of the stratification process, i.e., single-cell delamination, and little is known about how the delamination of individual cells expands into overall tissue stratification. Specifically, it remains unknown whether this transition propagates as a wave originating from a specific point of cell delamination or instead emerges from numerous origins, spreading through a disordered intermediate state, and what physics underlies this transition.

To bridge the gap between cell- and tissue-scale mechanics, we have combined three-dimensional (3D) quantifications and multicellular dynamics modeling. This approach elucidates how individual cells mechanically control the overall layer structure of the epithelium. Two primary objectives guided our study. First, we aimed to observe the collective cell dynamics in 3D space during epithelial stratification. Although the kinetic behaviors of individual cell delamination are well documented, the collective motions of cells throughout the entire stratification process remain largely unexplored. Specifically, the spatial distribution of delaminated cells during stratification and their subsequent influence on the delamination of other cells requires further clarification. Second, we focused on understanding the physical mechanisms underlying collective cell motion during stratification. Given the challenges of monitoring mechanical interactions in 3D tissue structures, we developed a 3D vertex model^34,35^. This model captures the dynamics of stratification, including 3D deformation and cellular arrangements. This approach allows for a comprehensive analysis of the 3D structures and mechanics involved in multicellular arrangements during epithelial stratification.

To elucidate the fundamental mechanisms of epithelial stratification, we observed the stratification process of Madin-Darby canine kidney (MDCK) cells cultured in a dish and employed a 3D vertex model to understand the physical mechanisms of stratification. Although MDCK cell cultures and vertex models have often been used to study cell delamination^36–38^, little has been done to extensively apply them to epithelial stratification. In both systems, we recapitulated the kinetic process of epithelial stratification at single-cell resolution, i.e., how individual cells delaminate and accumulate to form multilayer structures. To analyze this process, we quantified 3D cell configurations and their dynamics to reveal the physical concept of the structural transition with nucleation and growth, which could be applied to epithelial stratification. To understand the mechanisms of delamination, we performed analytical calculations using a 3D vertex model to reveal a mechanical instability inherent in the foam-like epithelial geometry. To validate the model predictions, we further conducted image analyses, atomic force microscopy (AFM) measurements, and pharmacological assays. Finally, we explored the applicability of the nucleation-growth concept to embryogenesis and carcinogenesis by observing skin development in mouse embryos and malignant transformation of intestinal cancer organoids. Throughout these interdisciplinary investigations, we conceptualized epithelial stratification as a nucleation-and-growth process governed by foam mechanics.

## Results

### Epithelial structure transitions from a monolayer to a multilayer

**Fig. 1:**
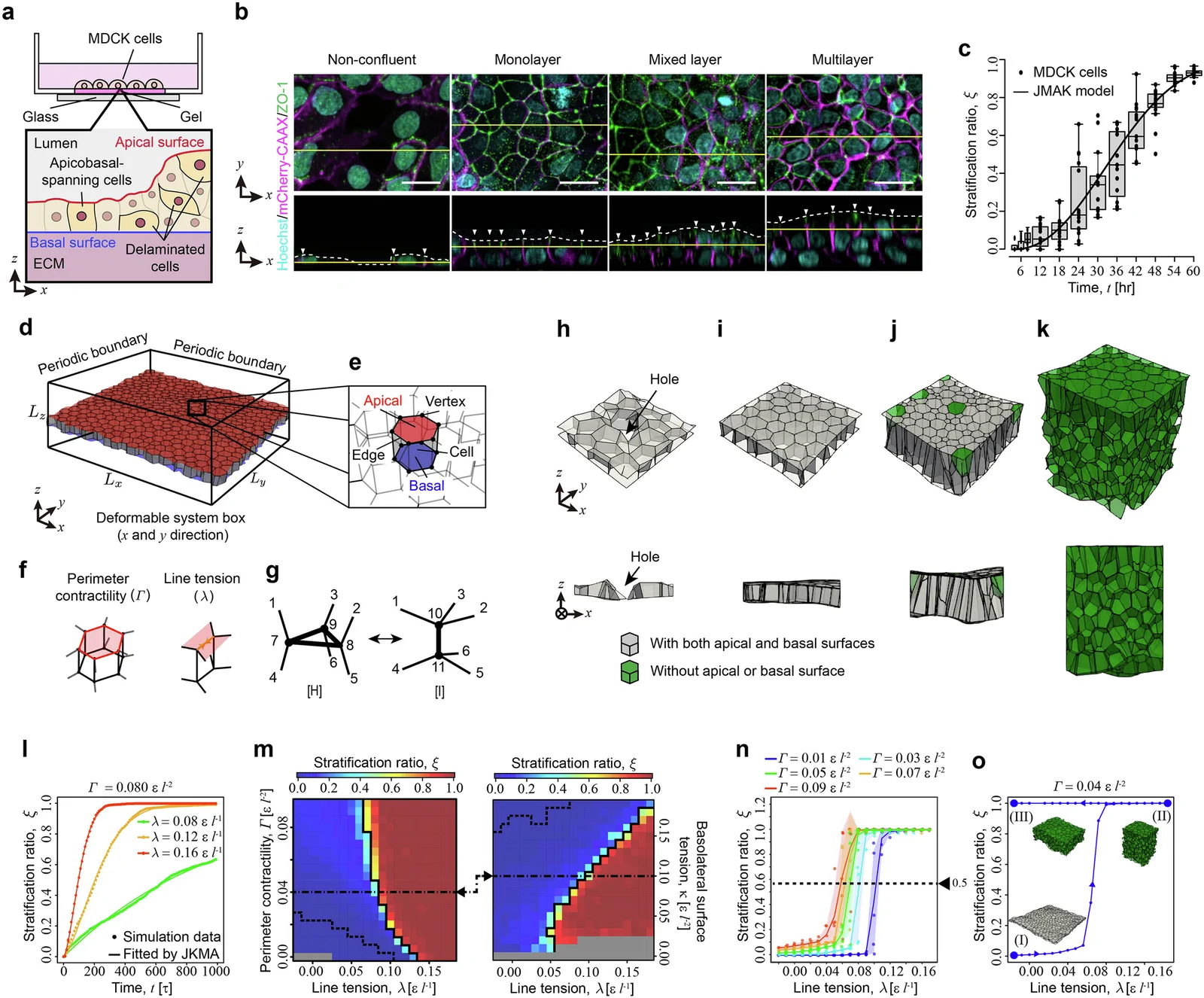
Epithelial cells stratify through a nucleation-growth process. **a** Schematic illustration of the MDCK cell culture setup used to observe epithelial stratification. Cells are categorized into two types: apicobasal-spanning cells, which possess both apical and basal surfaces, and delaminated cells, which lack an apical surface, a basal surface, or both. **b** Fluorescence images of MDCK cells stably expressing mCherry-CAAX, stained with a ZO-1 antibody and Hoechst. The top panel shows the *z*-projection, and the bottom panel displays a single *xz*-view image along the yellow line in the top panel. The white arrow highlights the ZO-1 signal in the *xz*-view, and the dotted line marks the apical surface. Scale bar: 20 µm. **c** Linear plots of the stratification ratio, *ξ*, as a function of time, *t*. Solid line shows JMAK model obtained by the least squares method (R² = 0.90). **d** Initial condition of a monolayer epithelial sheet in 3D space, where the system box is deformed by axial stress in the *xy* plane. **e** Enlarged image of a single cell within the epithelial sheet; surface colors indicate the apical (red), lateral (gray), and basal (blue) surfaces. **f** Schematic illustrations of apical junctional forces acting on individual apical cell perimeters: apical perimeter contractility, *Γ* (left), and individual apical cell edges: apical line tension, *λ* (right). **g** Definition of topological operations between patterns [H] and [I]. Epithelial structures after relaxation, showing different states: collapse (**h**), monolayer (**i**), partial multilayer (**j**), and multilayer (**k**), induced by varying apical junctional forces. Green cells represent those lacking apical, basal, or both surfaces. The parameters used were set to *Γ* = 0*, λ* = 0 (**h**), *Γ* = 0.015 *ε l*^−2^*, λ* = 0 (**i**), *Γ* = 0.075 *ε l*^−2^*, λ* = 0.03 *ε l*^−1^ (**j**), and *Γ* = 0.065 *ε l*^−2^*, λ* = 0.13 ε *l*^−1^ (**k**). **l** Linear plots of the stratification ratio, *ξ*, as a function of time, *t*, respectively. Dotted lines indicate simulation data. Solid line shows JMAK model fitted by the least squares method. **m** Stratification ratio, *ξ*, with respect to *Γ* and *λ* left, and *κ* and *λ* right. **n** Stratification ratio, *ξ*, as a function of *λ*. **o** Hysteresis behavior in epithelial stratification. Epithelial structures at the indicated points (I)–(III) are shown on the right. The results between points (I) and (II) were calculated starting from the monolayer initial condition shown in (**d**), and those between points (II) and (III) were obtained using the multilayer initial condition at point (II). The embedded images indicate epithelial structures at points (I)–(III). Images in **b** are representative of *N* = 4 biologically independent experiments with similar results. Data in **c** represent *n* = 15 fields of view analyzed across *N* = 3 biologically independent cell culture experiments. Box plots in **c**: center: median; bounds, 25th and 75th percentiles; whiskers, min and max. In **m**, solid and dotted lines indicate boundaries among the multilayer (*ξ* > 0.5), mixed layer (0.5 ≥ *ξ* > 0.0), and complete monolayer (*ξ* = 0.0). Gray regions indicate collapsed states. The data in **l**, **m**, and **o** indicate the average values calculated from *N* = 5 independent simulation runs with different initial conditions. In **n**, the solid line represents the mean, and the shaded band indicates the mean ± SD. Source data are provided as a Source Data file.

To understand the kinetic process of epithelial stratification, we employed simple MDCK cell cultures on a dish coated with collagen gel (Fig. 1a, see Materials and Methods). Although MDCK monolayers are generally stable, minor multilayering can occasionally occur even under standard culture. In addition, prior studies have shown that non-transformed MDCK cells can form multilayers under specific conditions^39,40^, and that apical stacking on an existing MDCK sheet is mechanically realizable^41^. Under our culture conditions (see Materials and Methods), this propensity was noticeably increased. We therefore used this setting in which the cells spread and stratified on the gel.

Under this condition, we identified four distinct stages during this stratification (Fig. 1b): a nonconfluent structure where cells partially occupied the dish, a complete monolayer with cells densely packed on the dish, a mixed layer featuring partially stratified cells, and a multilayer with cells mostly stratified. Throughout the stratification, the tight junction marker ZO-1 consistently localized at the cell-cell boundaries around the apical surface of the entire sheet (Fig. 1b). In contrast, cells delaminating from the apical side lost their junctions. Thus, the cells stratified from a monolayer to a multilayer structure while maintaining the apicobasal polarity of the entire tissue.

To understand this stratification process, we quantified its time evolution (detailed in Materials and Methods). The epithelium consists of two types of cells: apicobasal-spanning cells, which possess both apical and basal surfaces, and delaminated cells, which lack an apical surface, a basal surface, or both (Fig. 1a). Here, the apical surface refers to the surface of the cell facing the lumen or free space on the apical side of the epithelium, while the basal surface refers to the surface in contact with the extracellular matrix (ECM). Loss of either the apical surface, basal surface, or both leads to delamination.

Based on this classification, we introduced the stratification ratio, *ξ*, defined as the occupancy ratio of the multilayer domain on the plane of the dish:

1 $$\xi=1-\frac{{N}_{{{{\rm{ab}}}}}}{{N}_{{{{\rm{t}}}}}}.$$

In Eq. (1), ${N}_{{{{\rm{ab}}}}}$ represents the number of apicobasal-spanning cells of the epithelium, and ${N}_{{{{\rm{t}}}}}$ represents the total number of cells within the epithelium. We observe a gradual increase in *ξ* from 0 to approximately 1, with an S-shaped curve (Fig. 1c), signifying a structural crossover from a complete monolayer to a fully multilayer arrangement.

These findings raise two key questions: how epithelial stratification progresses across the tissue over time, and which cell-level behaviors give rise to this tissue-scale structural transition. To address these questions, we next constructed a cell-based mechanical model linking cellular behaviors to tissue-scale organization and used it together with quantitative observations of epithelial stratification at single-cell resolution to examine its dynamical and mechanical features.

### 3D foam mechanics-based modeling reproduces epithelial stratification

To explore the mechanical basis of epithelial stratification, we developed a 3D vertex model (detailed in Materials and Methods) based on the assumption that epithelial stratification can be regarded as a mechanical process of foam-like materials. This model overcomes the limitations of conventional models; while 3D particle models and 2D vertex models have unveiled the mechanics of stratification^13,43–45^, they cannot fully describe 3D cell deformations and arrangements.

Our model describes a 3D foam geometry of the epithelium equilibrated under stress-free boundary conditions, assuming balanced mechanical forces among cells (Fig. 1d; detailed in Materials and Methods). Cells have apicobasal polarity (Fig. 1e): based on observations of the tight junction marker ZO-1 (Fig. 1b). In our model, the epithelial sheet has two free surfaces, which we define as the apical and basal surfaces. Specifically, the upper free surface is defined as the apical surface, whereas the lower free surface is defined as the basal surface. Throughout the manuscript, we refer to the cell–cell interfaces and the basal surface collectively as the basolateral surfaces.

Moreover, we introduced two types of junctional contractility, well-established regulators of cell delamination: apical perimeter contractility of each cell, *Γ*, and line tension at apical junctions, *λ* (Fig. 1f). These parameters are known to describe apical behaviors of epithelial cells in 2D space^46^ and have been applied to those in 3D space^47–49^. While *Γ* homogenizes the size of apical cell surfaces across adjacent cells, *λ* works oppositely^30^.

The mechanical behaviors of cells with apicobasal polarity are given by the effective energy *U*, expressed as

2 $$U={{\sum}_{\alpha }^{{{{\rm{cell}}}}}} \, \frac{K}{2}{\left(\frac{{V}_{\alpha }}{{V}_{0}}-1\right)}^{2}+{{\sum}_{\alpha }^{{{{\rm{cell}}}}}} \, \kappa {A}_{\alpha }^{{{{\rm{bl}}}}}+{{\sum}_{\alpha }^{{{{\rm{cell}}}}}} \, {\varGamma \left({P}_{\alpha }\right)}^{2}+{{\sum}_{\left(\alpha \beta \right)}^{{{{\rm{junction}}}}}} \, \lambda {L}_{\left(\alpha \beta \right)}.$$

In Eq. (2), *K*, *V*_0_, *κ*, *Γ*, and *λ* are constants, and *K*, *V*_*0*_, *κ*, and *Γ* are positive. *α* and *β* denote cell indices, and (*αβ*) indicates a pair of the *α*th and *β*th cells. The first term represents the volume elastic energy, which restores the volume of each cell ${V}_{\alpha }$ to ${V}_{0}$ (set to ${l}^{3}$, where l represents the unit length), with the elastic constant *K*. The second term is the basolateral surface energy, characterized by the surface tension, *κ*, which acts to contract the basolateral surface area ${A}_{\alpha }^{{{{\rm{bl}}}}}$. The third term denotes the elastic energy associated with the apical junctional perimeter, where the elastic constant *Γ* drives contraction of the apical perimeter ${P}_{\alpha }$. The fourth term accounts for the tensile energy, where the tension constant *λ* contracts the length of the apical junction between neighboring cells, ${L}_{\left(\alpha \beta \right)}$. Because *K* and ${V}_{0}$ set the volume elasticity and preferred-volume scales and were kept fixed in this study, we independently varied *κ*, *Γ*, and *λ* in Eq. (2).

Using this model, we numerically calculated the energy relaxation process starting from an initial condition of a complete monolayer (Fig. 1d; detailed in Materials and Methods) and obtained an equilibrium structure at the local energy minimum. During this relaxation, cell configurations were rearranged in 3D space, leading to stratification. This cell rearrangement is described by introducing operations named [H]-[I] (Fig. 1g), as suggested in a previous study^34^. These operations cause the delamination of a cell from a monolayer to either the apical or basal side (Supplementary Fig. 1a-I), cell rearrangement within the plane of an epithelial sheet (Supplementary Fig. 1a-II), and the collapse of the epithelial sheet, creating a hole (Supplementary Fig. 1a-III). As in the experiments, we classified cells in the model according to their contacts with the apical and basal surfaces as apicobasal-spanning cells (Fig. 1a). Cells that lacked either the apical surface, the basal surface, or both were defined as delaminated cells.

By exploring the apical junctional parameters *Γ* and *λ*, we replicated all four epithelial structure types: a nonconfluent structure (Fig. 1h), a complete monolayer (Fig. 1i), a mixed layer (Fig. 1j), and a multilayer (Fig. 1k), corresponding to those observed in MDCK cell culture (Fig. 1b). We calculated the average number of layers in individual epithelia, which increased with *Γ* and *λ* (Supplementary Fig. 1b).

To analyze the dependence of the layer structure on the apical junctional parameters *Γ* and *λ*, we calculated the stratification ratio, *ξ* (Fig. 1m). At small values of *Γ* and *λ*, the epithelium formed an incomplete monolayer, which transitioned to a complete monolayer as *Γ* and *λ* slightly increased. As *Γ* increased further, the epithelial structure transitioned from a complete monolayer to a mixed layer, and as *λ* increased further, it transitioned to a complete multilayer structure. Quantitatively, *ξ* jumped from 0 to 1 with an increase in *λ* in a switch-like manner (Fig. 1n), indicating a structural transition from a monolayer to a multilayer. We also quantified the dependence of the layer structure on the apical junctional parameter, *λ*, and basolateral surface tension, *κ* (Fig. 1m). As *λ* increases and *κ* decreases, the epithelial structure transitioned from a monolayer to a multilayer structure. Quantitatively, *ξ* also jumped from 0 to 1 with an increase in *λ* and a decrease in *κ*. The stratification ratio depended more strongly on *λ* and *κ* than on *Γ* within the examined parameter range, indicating that *λ* and *κ* primarily control the mechanical balance leading to stratification. In contrast, *Γ* mainly shifted the onset of stratification without substantially altering the overall structural behavior.

Moreover, to investigate the structural transition characteristics, we varied *λ* and observed the evolution of *ξ* starting from the initial condition of either a monolayer or a multilayer structure (Fig. 1o). The monolayer-to-multilayer transition was observed when starting from a monolayer (Fig. 1o, from points I to II), but the multilayer-to-monolayer transition did not occur when starting from a multilayer (Fig. 1o, from points II to III). These results show that this transition exhibits hysteresis. Thus, our 3D foam-based model replicates the monolayer-to-multilayer transition observed in epithelial stratification, characterizing it as a first-order-like transition.

### The nucleation-growth process drives epithelial stratification

**Fig. 2:**
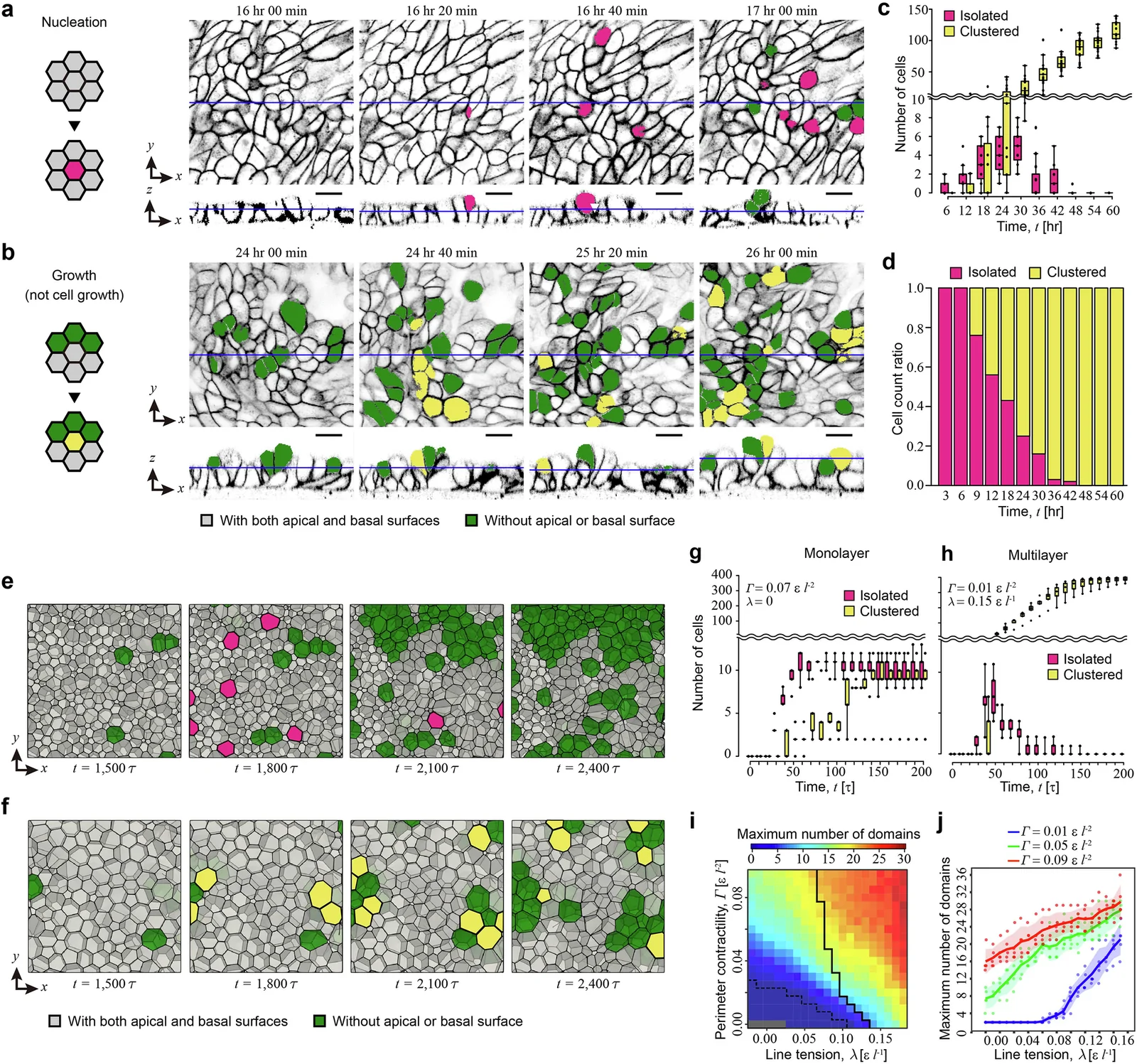
Nucleation-growth process drive epithelial stratification. **a**, **b** Schematic illustration (left) and corresponding experimental images (right) depicting nucleation and growth during stratification of MDCK cells. Red cells represent newly delaminated cells appearing in the monolayer, and yellow cells represent newly delaminated cells adjacent to existing ones. Both were classified as lacking apical or basal surfaces. Scale bar: 20 µm. **c** Temporal changes in the number of isolated versus clustered delaminated cells. **d** Temporal changes in the ratio of isolated and clustered delaminated cells. **e**, **f** Nucleation and growth during stratification of 3D vertex model. These images show the relaxation process of the simulation. Green cells represent those lacking apical, basal, or both surfaces. Red cells represent newly delaminated cells appearing in the monolayer, and yellow cells represent newly delaminated cells adjacent to existing ones. The parameters used were set to *Γ* = 0.085 *ε l*^−2^*, λ* = 0.07 *ε l*^−1^ (**e**) and *Γ* = 0.005 *ε l*^−2^*, λ* = 0.13 *ε l*^−1^ (**f**). **g**, **h** Temporal changes in the number of isolated versus clustered delaminated cells under monolayer and multilayer conditions, respectively. **i** Maximum number of domains over time with respect to *Γ* and *λ*. **j** Maximum number of domains over time as a function of *λ*. Images in **a** and **b** are representative of *N* = 3 biologically independent experiments with similar results. Data in **c** and **d** represent *n* = 15 fields of view from *N* = 3 biologically independent cell culture experiments (5 fields of view per experiments). Box plots in **c**, **g**, and **h**: center: median; bounds, 25th and 75th percentiles; whiskers, min and max. In **i**, solid and dotted lines indicate boundaries among the multilayer (*ξ* > 0.5), mixed layer (0.5 ≥ *ξ* > 0.0), and complete monolayer (*ξ* = 0.0). Gray regions indicate collapsed states. Data in **g**–**i** are presented as mean values from *N* = 5 independent simulation runs with different initial conditions. The solid line in **j** represents the mean, and the shaded band indicates the mean ± SD. Source data are provided as a Source Data file.

Next, to understand how individual cell behaviors contribute to epithelial stratification, we examined the dynamics of delamination. During stratification, individual cells delaminated by losing their apical or basal side, thereby dividing the epithelium into monolayer and multilayer domains. We identified two characteristic types of delamination events: nucleation and growth (Fig. 2a, e). Nucleation refers to delamination events that occur individually within a monolayer domain (Fig. 2b, f). Growth refers to cell delamination events that occur at the boundary of the monolayer adjacent to a multilayer domain. These events are analogous to the physical processes of nucleation and growth observed in structural transformations such as crystallization.

To quantify the temporal progression of these events in MDCK cell culture, we classified delaminated cells as either isolated or clustered, depending on whether they were adjacent to other delaminated cells (detailed in Materials and Methods). Isolated delaminations increased during the early stage of stratification, followed by an increase in clustered delaminated cells (Fig. 2c). Consistently, the increase in clustered cells eventually exceeded that of isolated delaminations (Fig. 2d). This temporal sequence is reminiscent of a nucleation-growth process in structural transitions, in which local delamination events act as nucleation sites and subsequently expand into multilayer domains.

Based on this nucleation-growth-like behavior, we applied the Johnson-Mehl-Avrami-Kolmogorov (JMAK) model^42^ to the time evolution of the stratification ratio *ξ*. The JMAK model describes the temporal progression of nucleation-and-growth processes and is expressed as:

3 $$\xi (t)=1-\exp \left[-k{(t-{t}_{0})}^{\nu }\right].$$

Here, *k* represents an effective growth rate, ${t}_{0}$ represents the initiation time, and *ν* represents the Avrami exponent. The time evolution of *ξ* in MDCK cell culture was well fitted by the JMAK model (Fig. 1c, Supplementary Fig. 2a; detailed in Materials and Methods), with a high coefficient of determination (R² = 0.90). The Avrami exponent was estimated to be *ν* = 2.29. Because the Avrami exponent reflects the effective dimensionality and spatial distribution of nucleation and growth, this value suggests that epithelial stratification follows a constrained nucleation-growth-like process rather than spatially uniform delamination across the entire sheet.

We next examined whether similar nucleation-growth-like dynamics emerged in the 3D vertex model. By tracking individual cell behaviors during numerical simulations, we identified both nucleation and growth events, as observed in MDCK cell culture (Fig. 2e, f). Consistently, simulations showed that nucleation initially occurred under both monolayer and multilayer-forming conditions, whereas subsequent growth was observed only under multilayer-forming conditions (Fig. 2g, h). These results suggest that the persistence of domain growth, rather than nucleation alone, is associated with multilayer formation.

To quantitatively compare the simulated and experimental kinetics, we also fitted the time evolution of *ξ* in the simulations using the JMAK model (Fig. 1l, Supplementary Fig. 2b), yielding Avrami exponents ranging from 1.0 to 2.69 (Supplementary Fig. 2c). The decrease in *ν* with increasing *Γ* suggests that nucleation sites became more spatially restricted as *Γ* increased. Notably, the parameter region with low *Γ* and high *λ* showed an Avrami exponent closest to that observed in MDCK cell culture (*ν* ≈ 2.29), and this region also corresponded to a similar number of layers (Supplementary Fig. 1b). This quantitative agreement suggests that the model recapitulates the kinetic features of epithelial stratification at the single-cell level.

To further characterize the nucleation-growth process in the simulations, we analyzed the number and size of multilayer domains during epithelial stratification (Fig. 2i, j; Supplementary Fig. 2d, e). Domains were quantified by identifying contiguous multilayer regions. Across the examined Γ*–*λ parameter range, stratification proceeded mainly through the growth of existing domains, whereas the number of newly formed domains varied depending on the apical junctional parameters. Consistently, analysis of the nucleation ratio, ${\psi }_{{{{\rm{n}}}}}$, and growth ratio, ${\psi }_{{{{\rm{g}}}}}$, showed that ${\psi }_{{{{\rm{g}}}}}$ more clearly distinguished monolayer and multilayer states than ${\psi }_{{{{\rm{n}}}}}$ (Supplementary Fig. 2f, g). These results indicate that apical junctional parameters modulate the frequency of nucleation events, whereas subsequent domain growth is the major contributor to multilayer expansion.

These results demonstrate that, in both the MDCK cell culture and the simulations based on foam mechanics, a small portion of cells initially delaminates individually, and the multilayer domains gradually expand through domain growth (Fig. 2a, b, e, f), rather than the majority of cells delaminating simultaneously across the entire epithelium. Consequently, under the conditions corresponding to MDCK stratification, the monolayer-to-multilayer transition is better described as a nucleation-growth process than as spinodal-decomposition-like behavior.

### Foam-geometric instability induces the nucleation-growth process

To understand the mechanisms inducing nucleation and domain growth (i.e., single cell delamination), we developed a theoretical model based on our previous theory^30^. In this model, we focus on the deformations of a single cell and its adjacent cells within a planar monolayer and derive an energy function for a delaminating cell (see Materials and Methods). This model simplifies the situation by approximating Eq. (2) using a mean-field approach, in which the heterogeneity of surrounding cell shapes is disregarded.

**Fig. 3:**
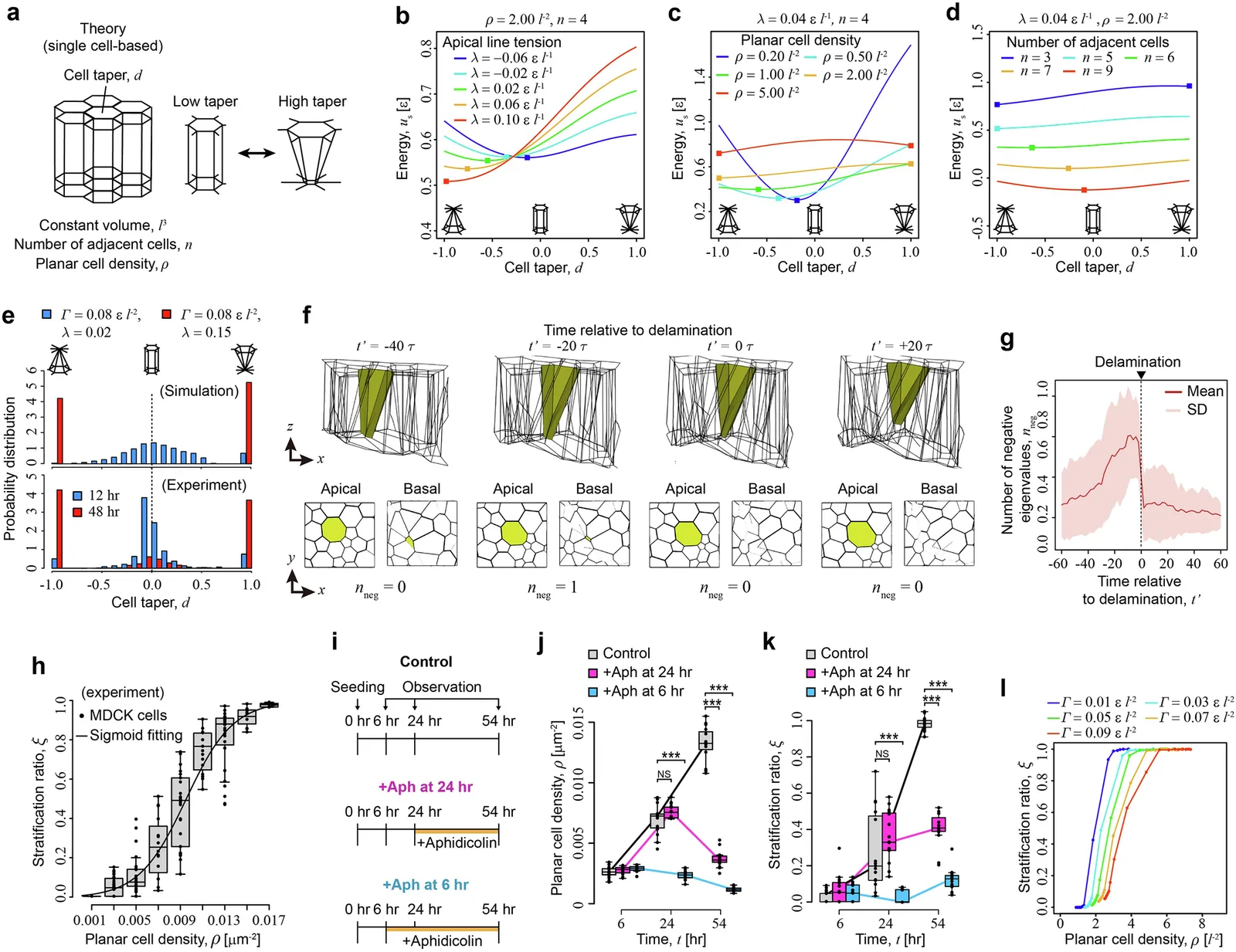
Foam-geometric instability induces epithelial stratification with scutoid formation. **a** Theoretical model describing the epithelial sheet 3D foam geometry. A single center cell embedded within a planar sheet is modeled as a regular frustum, with its geometry uniquely determined by the number of adjacent cells, *n*, planar cell density, *ρ*, and cell taper, *d*. **b**–**d** Energy landscapes as a function of *d*, with respect to apical line tension *λ*, *ρ*, and *n*, respectively, derived from the theoretical model. Squares indicate energy minima. **e** Probability distribution of the cell taper, d, calculated from 3D vertex-model simulations and experimental MDCK data. Blue and red indicate low- and high-stratification conditions, respectively, corresponding to $\lambda=0.02\,\varepsilon {l}^{-1}$ and $\lambda=0.15\,\varepsilon {l}^{-1}$ in simulations, and 12 and 48 h in MDCK cultures. Simulations were performed with $\varGamma=0.08\,\varepsilon {l}^{-2}$. **f**, **g** Temporal evolution of mechanical instability during cell delamination in the 3D vertex model. In **f**, representative snapshots show cell delamination. Top and bottom images show 3D and cross-sectional views, respectively. The number of negative eigenvalues of the Hessian matrix for the delaminating cell, ${n}_{{{{\rm{n}}}}{{{\rm{eg}}}}}$, is indicated below each image. In **g**, ${n}_{{{{\rm{n}}}}{{{\rm{eg}}}}}$ is plotted as a function of time relative to delamination*, t’*. Data represent mean ± s.d. **h** Stratification ratio, *ξ*, as a function of planar cell density *ρ* in the MDCK experiment. The solid line in **h** represents a fitted sigmoid function. **i**–**k** Aphidicolin perturbation assays in MDCK cells. Aphidicolin was added at 6 or 24 h after seeding to suppress the time-dependent increase in planar cell density. **i** Treatment scheme. **j** Planar cell density, *ρ*, as a function of time. **k** Stratification ratio, *ξ*, as a function of time. **l** Stratification ratio, *ξ*, as a function of planar cell density *ρ* in the 3D vertex model, for different values of *Γ*. Data in **e**, **h**–**k** represent *n* = 15 fields of view analyzed across *N* = 3 biologically independent cell culture experiments. Box plots in **h**, **j**, and **k**: center, median; bounds, 25th and 75th percentiles; whiskers, min and max**;** ***P < 0.001 (two-sided Welch’s t-test). For **j**, *P* = 4.37 × 10⁻¹⁴ (+Aph 6 h) and *P* = 0.62 (+Aph 24 h) at 24 h; *P* = 1.11 × 10⁻^22^ (+Aph 6 h) and *P* = 9.25 × 10⁻²⁰ (+Aph 24 h) at 54 h (all versus control). For **k**, *P* = 5.66 × 10⁻^5^ (+Aph 6 h) and *P* = 0.38 (+Aph 24 h) at 24 h; *P* = 4.55 × 10⁻^26^ (+Aph 6 h) and *P* = 1.31 × 10^−18^ (+Aph 24 h) at 54 h (all versus control). The data in **e**, **g**, and **l** indicate the average values calculated from *N* = 5 independent simulation runs with different initial conditions. Source data are provided as a Source Data file.

We examined the dependence of stable cell shapes on apical line tension (*λ*), planar cell density (*ρ*), and the number of adjacent cells (*n*) by analyzing energy landscapes. Planar cell density, *ρ*, was defined as the number of cells per unit projected area (see Materials and Methods). To describe the shape of a delaminating cell, we introduced the cell taper, *d* (Fig. 3a), which satisfies −1 <*d* < 1 when the cell is embedded in a monolayer and becomes −1 or 1 when it delaminates in the basal or apical direction. Stable cell states were determined by identifying *d* which minimizes the energy function, denoted by d^*^. We found that as *λ*, *ρ*, and *n* were varied, d^*^ shifted from −1 <d^*^ < 1 to d^*^ = −1 and/or d^*^ = 1, indicating the transition from an embedded to a delaminated state (Fig. 3b–d). Specifically, d^*^ bifurcated from −1 <d^*^ < 1 to d^*^ = −1 and 1 as a function of *ρ* and *n* (Fig. 3c, d). These results indicate that mechanical instability is inherent in the foam-like epithelial geometry; either an increase in *λ*, an increase in *ρ*, or a decrease in *n* leads to cell delamination.

To examine whether this predicted mechanical instability is reflected in epithelial stratification, we calculated the probability distribution of cell taper, *d*, in MDCK cell culture and 3D vertex-model simulations (Fig. 3e). Both experiments and simulations showed a trimodal distribution around *d* = −1, 0, and 1, with two distinct gaps between *d* = −1 and 0 and between *d* = 0 and 1. This pattern indicates that cells predominantly adopt either an embedded state or apically or basally delaminated states, rather than remaining in intermediate configurations. These observations are consistent with the idea that loss of stability of the embedded state drives cells toward delamination through an inherent mechanical instability.

Furthermore, to quantitatively assess local mechanical stability in the simulations, we analyzed the Hessian matrix of the effective energy around individual delaminating cells in the 3D vertex model (see Materials and Methods). Cells approaching delamination showed an increase in the number of negative eigenvalues immediately before delamination, indicating a loss of local mechanical stability in the energy landscape (Fig. 3f, g). These results suggest that cell delamination, and consequently epithelial stratification, is preceded by mechanically unstable cell states.

### Planar cell density drives nucleation and growth in MDCK cells

While the dependences on *λ*, *ρ*, and *n* were consistent with previous reports identifying contributors to cell delamination, including apical junctional forces^19–24^, planar cell density^25–27^, and cell geometry^28–31^, the key driving force of epithelial stratification in MDCK cell culture (Fig. 1b) remained unclear. To address this, we examined each contributor experimentally.

First, to determine whether the average value of the apical line tension *λ* drives epithelial stratification, we measured the apparent Young’s moduli at apical junctions using AFM (Supplementary Fig. 3a), which reflects the average value of junctional tensions^50^. We then examined the correlations between the modulus and the stratification ratio, *ξ* and found that the apical junctional modulus decreased significantly with increasing *ξ* (Supplementary Fig. 3b), which is in stark contrast to the theoretical prediction (Fig. 3b). These results suggest that apical junctional tension does not act as a key driving force behind stratification in MDCK cell culture.

Second, to examine the contribution of the planar cell density, *ρ*, to epithelial stratification, we quantified *ρ* in MDCK cell culture (Supplementary Fig. 3c). We observed a monotonic increase in *ρ* over time. Moreover, *ξ* increased with *ρ* in a switch-like manner and was well fitted by a sigmoidal curve (Fig. 3h). To further test this association, we inhibited cell proliferation using aphidicolin at 6 and 24 hours after seeding (Fig. 3i), corresponding to the periods when nucleation and growth predominantly occur, respectively (Fig. 2a, b). Aphidicolin treatment significantly suppressed the increase in *ρ* compared with the control (Fig. 3j) and consistently suppressed the increase in *ξ* at the corresponding time points (Fig. 3k). These results suggest that increasing planar cell density contributes to the progression of epithelial stratification in MDCK cell culture.

We next examined how planar cell density contributes to epithelial stratification in the simulations. Planar cell density varied depending on *Γ* and *λ* and increased with *λ* across the examined parameter range (Supplementary Fig. 3d, e). The stratification ratio generally increased with increasing density, although the relationship between density and layer structure varied across parameter regimes (Fig. 3l). Because domain growth occurs adjacent to pre-existing multilayer domains, we next focused on differences around these domains. We classified the monolayer region into potential nucleation and growth regions (Supplementary Fig. 3f). The potential nucleation region consisted of cells located away from existing multilayer domains, whereas the potential growth region consisted of cells adjacent to multilayer domains. We then calculated the frequency of delamination events in each region (Supplementary Fig. 3g).

Across most values of *Γ* and *λ*, delamination events occurred more frequently in the potential growth region than in the potential nucleation region. Moreover, the apparent planar cell density, defined for each cell as the inverse of the mean of its apical and basal areas (see Materials and Methods), was consistently higher on average in the potential growth region than in the potential nucleation region (Supplementary Fig. 3h). These results suggest that pre-existing multilayer domains locally increase cell crowding, thereby facilitating nearby delamination events and promoting cooperative domain growth during epithelial stratification.

These results suggest that both nucleation and growth induced by the increase in cell density play critical roles in driving epithelial stratification. This finding aligns with previous studies highlighting the influence of cell density on individual cell delamination^25–27^ as well as our analytical calculations (Fig. 3c). Thus, planar cell density, rather than apical junctional tension, is the key driving force behind epithelial stratification in MDCK cell culture.

### Cell delamination proceeds via scutoid formation

**Fig. 4:**
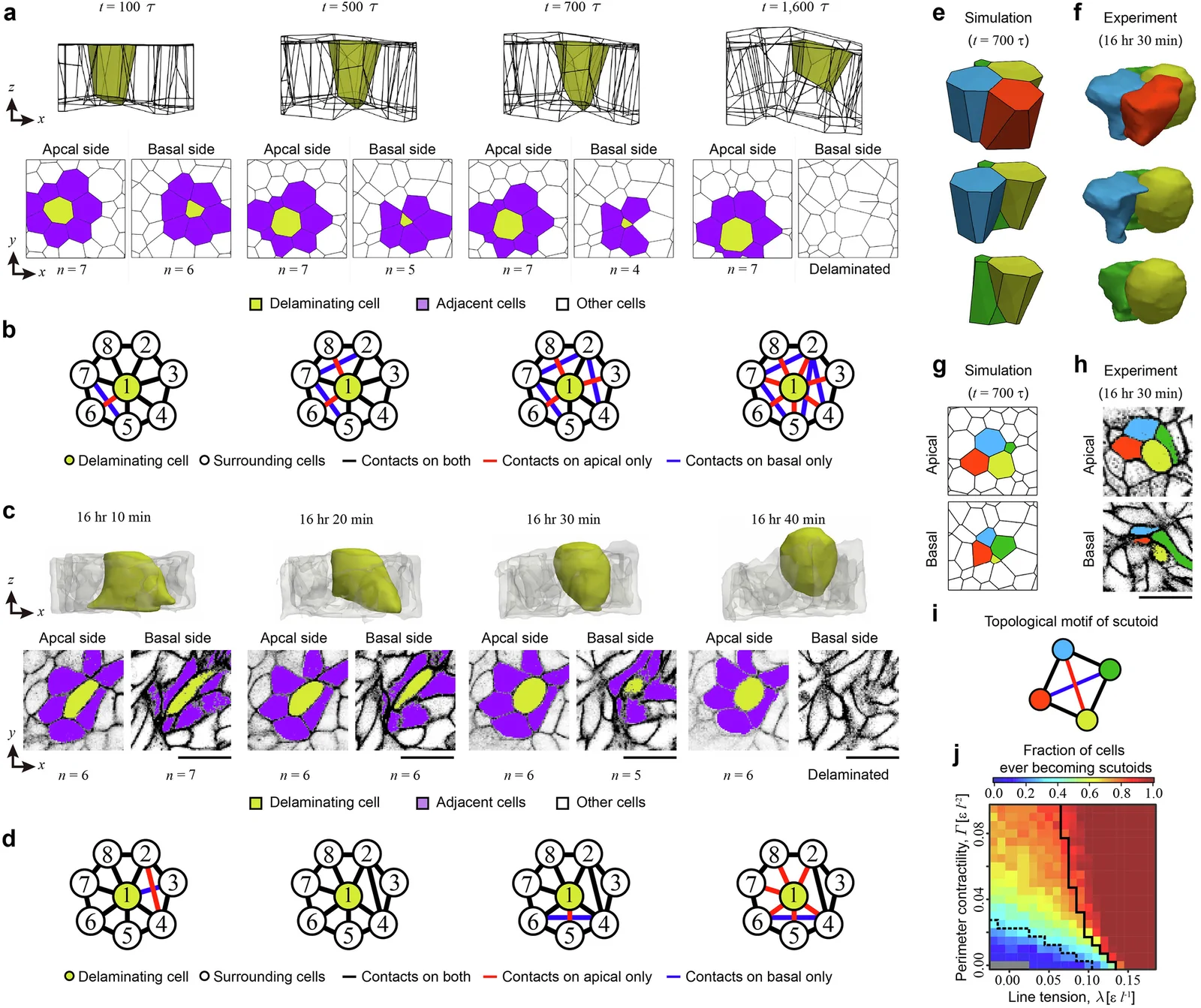
Cell delamination proceeds with formation of a scutoid. Delamination processes in the 3D vertex model (**a**) and MDCK cell culture (**c**). The top images depict 3D views, and the bottom images show cross-sectional views of the apical and basal surfaces, with the number of cells adjacent to delaminating cells, *n*, indicated below. In the top and bottom panels, yellow highlights the cell delaminating toward the apical side as cells lose basal surfaces. In the bottom panels, purple marks cells adjacent to delaminating cells. Scale bar: 10 µm in (**c**). **b**, **d** Topological networks of cell-cell contacts on the apical and basal surfaces. Yellow and white nodes represent delaminating and neighboring cells, respectively. Black, red, and blue edges indicate cell-cell contacts present on both the apical and basal sides, the apical side only, and the basal side only. Scutoid formation during delamination in the 3D vertex model (**e**, **g**) and MDCK cell culture (**f**, **h**). **e**, **f** 3D geometries of cells forming scutoids. The yellow cells correspond to those in (**a**, **b**) and (**c**, **d**), respectively. **g**, **h** Apical and basal shapes of the cells shown in (**e**) and (**f**), respectively. Scale bar: 10 µm. **i** Topological motif characteristic of scutoid geometry. This motif corresponds to the cell geometry shown in (**e**–**h**). Yellow, red, blue, and green nodes in **i** represent the respective cells in (**e**–**h**). **j** Fraction of cells ever becoming scutoids with respect to *Γ* and *λ*. In **a**, **b**, **e**, and **g**, the parameters used were set to *Γ* = 0.085 *ε l*^−2^ and *λ* = 0.07 *ε l*^−1^. In **j**, solid and dotted lines indicate boundaries among the multilayer (*ξ* > 0.5), mixed layer (0.5 ≥ *ξ* > 0.0), and complete monolayer (*ξ* = 0.0). Gray regions indicate collapsed states. The data in **j** indicate the average values calculated from *N* = 5 independent simulation runs with different initial conditions. Source data are provided as a Source Data file.

To investigate the influence of *n* on stratification, we observed the changes in cell geometries during the delamination process in both simulations and experiments (Fig. 4a–j). During stratification, each cell gradually reduced the number of adjacent cells and lost either its apical or basal side to delaminate from the monolayer (Fig. 4a, c), which aligns with our theory (Fig. 3d). These processes can be represented by topological networks of cell-cell contacts (Fig. 4b, d), where cells are described as nodes and contacts as edges. Specifically, the contact edges were categorized into three types: (1) contacts through lateral boundary faces that connect to both the apical and basal surfaces (black lines); (2) contacts that connect only to the apical surface (red lines); and (3) contacts that connect only to the basal surface (blue lines). In both experimental and simulation results, although contact edges on the apical side were largely preserved, those on the basal side changed dynamically; contacts between delaminating cells and their neighboring cells were replaced by new contacts formed between neighboring cells on the basal side.

Interestingly, during the delamination process, we found that cells formed a scutoid geometry^51–53^, which emerged when cell-cell contacts differed between the apical and basal sides. In such cases, cell-cell contacts switched along the lateral surfaces between neighboring cells (Fig. 4e, f). Specifically, the blue and yellow cells made contact on the apical side, whereas the red and green cells made contact on the basal side (Fig. 4g, h). The scutoid can be characterized by a specific topological motif within a local network of cell-cell contacts (Fig. 4i). In this motif, four cells, represented as nodes, form a quadrilateral contact topology, within which each cell exhibits different edge connections on the apical and basal sides, thereby adopting a scutoid geometry. Such motifs can be identified in the topological contact networks observed during stratification (Fig. 4b, d). For example, in the numerical simulation, cells 1 and 3 formed a contact, while cells 2 and 4 formed contacts at *t* = 500 τ, indicating that cells 1–4 collectively form scutoids (Fig. 4b).

In epithelia with finite thickness, cell–cell contact patterns can differ between the apical and basal surfaces. When cells rearrange within such epithelial sheets, they can transiently adopt scutoid geometries, which allow topological transitions through the exchange of cell–cell contacts along the apicobasal axis. In line with this idea, most scutoids appeared transiently during cell delamination (Fig. 4b, d). Moreover, scutoids became more frequent as stratification progressed and were observed in both mixed layer and complete multilayer structures (Fig. 4j). However, within the complete multilayer regime near the monolayer boundary at low *Γ*, scutoid numbers remained relatively low. This may reflect a regime in which domain growth contributes relatively more than new domain formation (Fig. 2i; Supplementary Figs. 2d, e and 3g). Scutoid formation was associated with an increase in the total number of cell–cell contacts across the tissue, suggesting that it facilitates the neighbor rearrangements required for delamination. Through this rearrangement process, delaminating cells ultimately reduce their contacts on either the apical or basal side, allowing them to exit the epithelial layer.

These results demonstrate that mechanical instability inherent in the foam-like epithelial geometry induces nucleation and domain growth. This instability can be triggered by an increase in apical junctional tension or planar cell density. During cell delamination, cells gradually lose either their apical or basal side while forming a scutoid geometry to reduce the number of adjacent cells, which further contributes to the onset of instability. The accumulation of cell delaminations leads to the structural transition of the epithelial structure from a monolayer to a multilayer.

### Nucleation-growth concept applies to embryogenesis and carcinogenesis

**Fig. 5:**
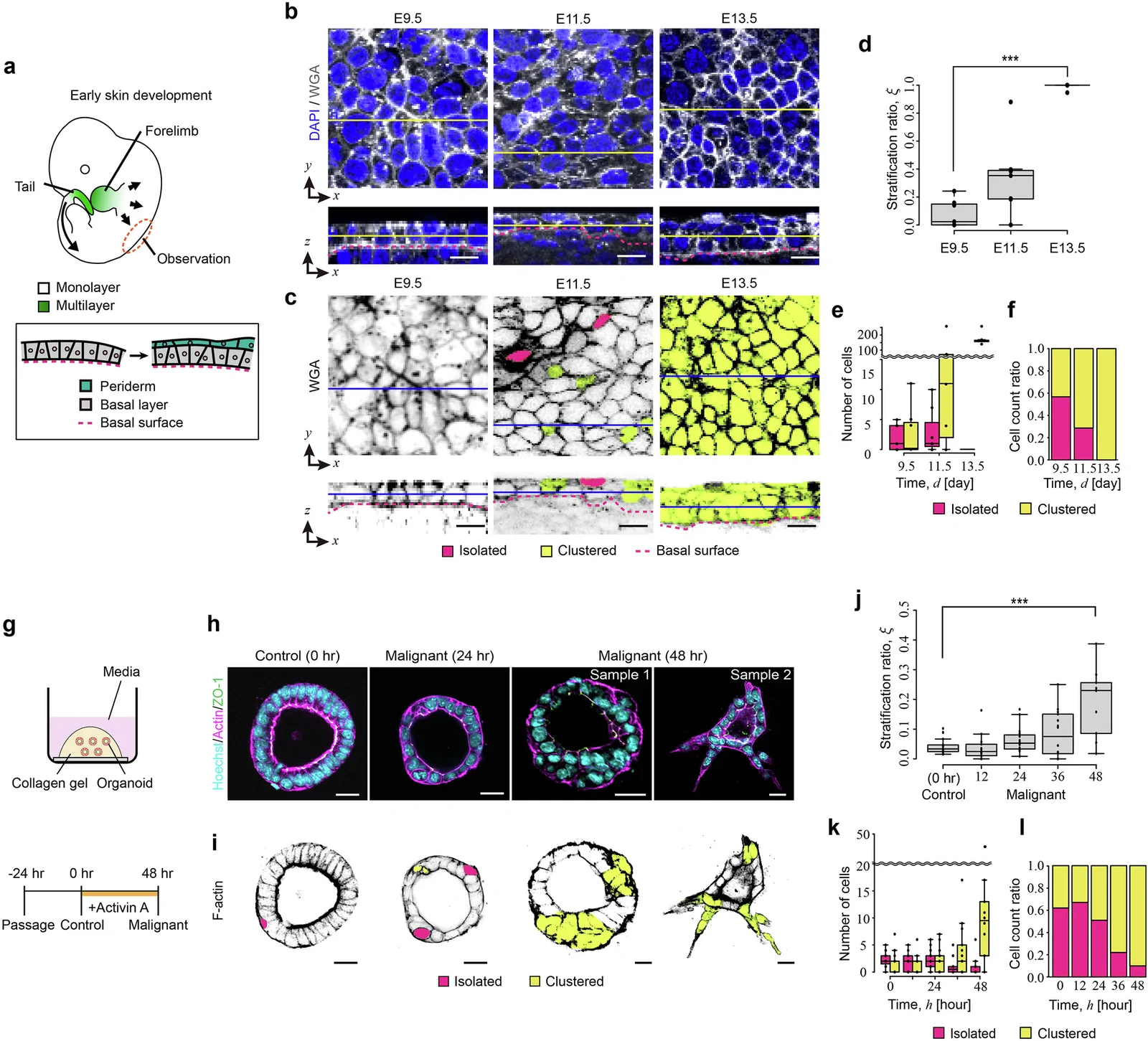
Nucleation-growth concept applies to embryogenesis and carcinogenesis. **a**–**f** Data for mouse skin development. **a** Schematic illustration of epidermal stratification progression in the entire mouse embryo. **b**, **c** Fluorescent section images of mouse skin and modified images highlighting delaminated cells. The skin is a monolayer in E9.5, mixed layer in E11.5, and multilayer structure in E13.5. Red and yellow cells represent isolated and clustered cells. The top panel shows the xy-view, and the bottom panel shows the *xz*-view. Yellow and blue lines indicate cross sections. The dotted lines mark the basal surfaces. Scale bar: 10 µm. In **b**, epidermal tissues were stained with wheat germ agglutinin (WGA) antibody and DAPI. **d** Stratification ratio, *ξ*, in each stage. **e** Temporal changes in the number of isolated versus clustered delaminated cells. **f** Temporal changes in the ratio of isolated and clustered delaminated cells. **g**–**l** Data for malignant transformation of intestinal cancer organoid. **g** Schematic illustration of cancer organoid culture and malignant phenotype induction. Fluorescent section images (**h**) and modified images highlighting delaminated cells (**i**), respectively. In **h**, organoids were stained with a ZO-1 antibody, phalloidin, and Hoechst. Scale bar indicates 20 µm. In **i**, red and yellow cells represent isolated and clustered cells. **j** Stratification ratio, *ξ*, under control and malignant induction. **k** Temporal changes in the number of isolated versus clustered delaminated cells. **l** Temporal changes in the ratio of isolated and clustered delaminated cells. Images and data in **b**–**f** were obtained from biologically independent mice, *N* = 8, 7, 7 at E9.5, E11.5, E13.5, respectively. In **c** and **i**, isolated and clustered delaminated cells are colored red and yellow, respectively. Images and data in **h**–**l** were obtained from *n* = 19, 13, 16, 14, and 10 fields of view obtained across *N* = 4, 4, 3, 3, and 5 biologically independent experiments at 0, 12, 24, 36, and 48 h, respectively. Box plots in **d**–**f**, **j**–**l**: center: median; bounds, 25th and 75th percentiles; whiskers, min and max; ***P < 0.001 (two-sided Welch’s t-test). For **d**, *P* = 1.53 × 10^−12^. For **j**, *P* = 6.01 × 10^−6^. Source data are provided as a Source Data file.

To determine whether the nucleation-growth concept applies to embryogenesis and carcinogenesis, we examined the epithelial stratification processes during mouse skin development and malignant transformation of mouse intestinal cancer organoids (Fig. 5). Skin development involves epithelial stratification, during which the epidermal monolayer transitions into a multilayer structure. Initially, basal cells delaminate and give rise to an intermediate suprabasal layer composed of poorly differentiated cells. This layer subsequently differentiates into committed periderm cells that cover the epidermis^12,54,55^ (Fig. 5a). This stratification appears to initiate at the tips of the limbs and tail and gradually extends across the body^54^. However, the mechanism underlying the spread of the multilayer domain remains poorly understood. To address this issue, we analyzed the dorsal skin of mouse embryos (Fig. 5a; see Materials and Methods), identifying three distinct structural states from embryonic day E9.5 to E13.5: near a monolayer, a mixed layer, and a multilayer (Fig. 5b). Spatial analysis of delaminated cells in the epidermis revealed two patterns: isolated and clustered arrangements (Fig. 5c). These observations indicate that epidermal stratification proceeds through both nucleation and growth, rather than through continuous growth of multilayer domains.

To clarify whether epidermal stratification proceeds via a nucleation and growth process, we quantified the layer structures of the epidermis. The stratification ratio increased from approximately 0.08 to 1.0 during the monolayer-to-multilayer transition (Fig. 5d). Moreover, to understand the time evolution of the nucleation and growth process during skin development, we determined whether the cells were isolated or clustered. Isolated cells increased during the early stages of stratification, followed by an increase in clustered cells, similar to the behavior observed in MDCK cells (Fig. 5e, f). These findings suggest that the concept of a structural transition, involving nucleation and growth, is applicable to skin development.

To investigate whether this concept also applies to cancer progression, we studied early-stage cancer cell invasion, where precancerous epithelial cells undergo abnormal stratification leading to invasion. For this purpose, we used key cancer-driving mutations in mouse intestinal cancer organoids: *Apc* (A), *Kras* (K), *Tgfbr2* (T), *Trp53* (P), and *Fbxw7* (F) (see Materials and Methods) ^56^. A previous study demonstrated that exposure to activin A in culture (Fig. 5g) induces malignant transformation in these organoids, mimicking in vivo cancerous behaviors^57^. Specifically, organoids cultured without activin A formed spherical cysts, whereas those exposed to activin A displayed malignant morphologies, such as heterogeneous spherical or protruded, star-like structures (Fig. 5h). At 0 hours, organoids exhibited a monolayer structure with a few isolated delaminated cells. By 48 hours post-transformation, these organoids transitioned to a multilayer structure with clustered delaminated cells (Fig. 5i).

Consistent with the observations, stratification ratios increased from approximately 0.02 to 0.25 during this transition (Fig. 5j). Moreover, to understand the time evolution of the nucleation and growth process during malignant transformation, we determined whether cells were isolated or clustered (Fig. 5k, l). Isolated cells increased during the early stages of stratification, followed by an increase in clustered cells, similar to the behavior observed in MDCK cells. These results suggest the analogy between the structural transition involving nucleation and growth and malignant transformation. Taken together, our findings demonstrate that the nucleation-growth concept applies beyond only MDCK cell culture to embryogenesis and carcinogenesis.

## Discussion

**Fig. 6:**
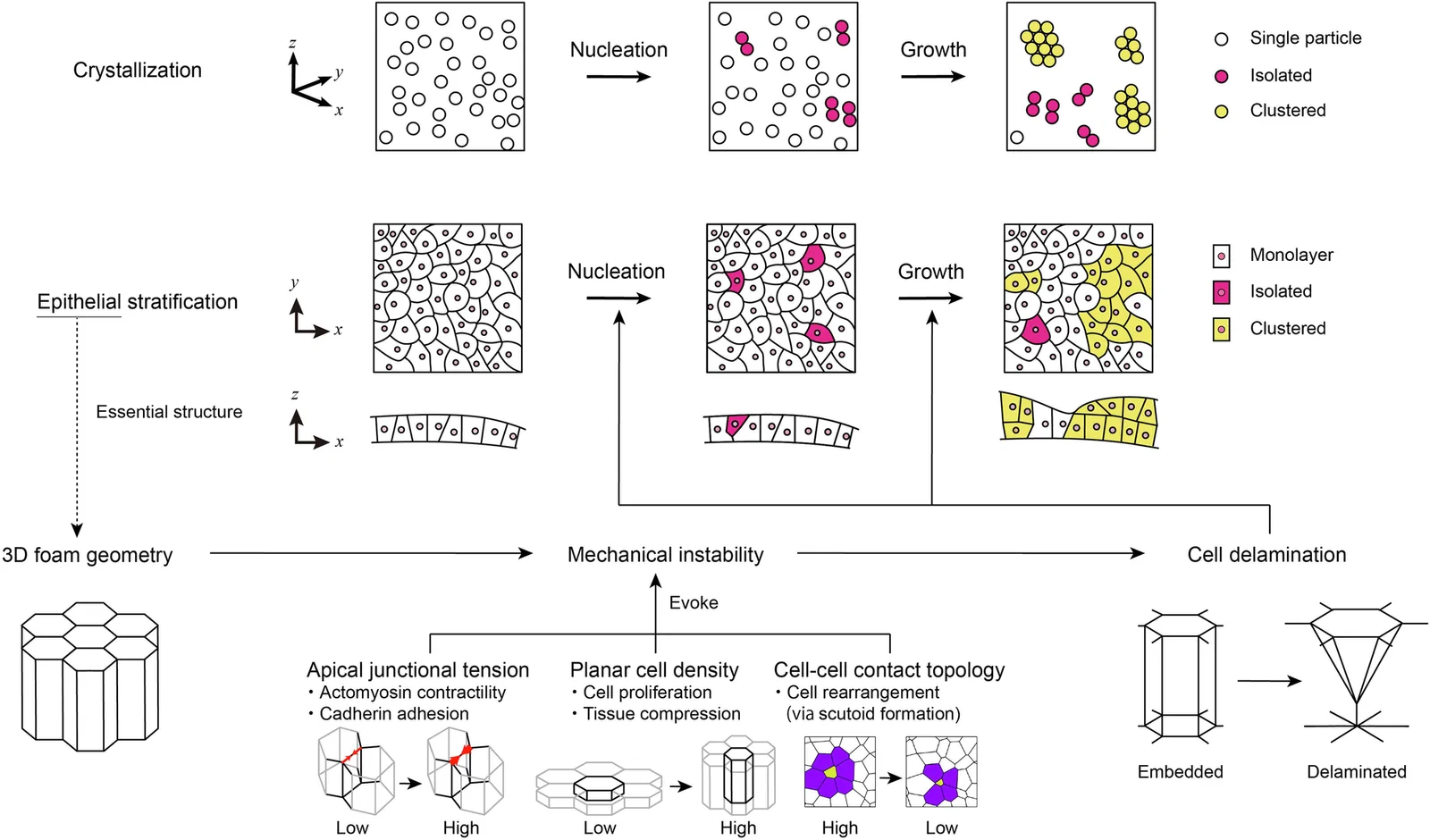
Epithelium stratifies via nucleation and growth induced by foam-geometric instability. Epithelial stratification can be understood as a structural transition, such as crystallization, that involves a nucleation-growth process. In this framework, stratification represents a structural transition from a monolayer to a multilayer configuration. The fundamental process driving this transition is cell delamination, which arises from mechanical instability inherent in the 3D geometry of the epithelial sheet. This instability can be evoked by various factors, including increased apical junctional tension due to actomyosin contractility and cadherin-mediated adhesion, increased planar cell density resulting from cell proliferation and tissue compression, and changes in cell-cell contact topology caused by rearrangements associated with scutoid formation.

In this study, we elucidated the physical mechanism underlying epithelial stratification, focusing on the transition from a monolayer to a multilayer structure (Fig. 6). We found that epithelial stratification proceeds as a switch-like structural transition involving nucleation and growth. The elementary process of both nucleation and growth, namely single-cell delamination, is induced by the mechanical instability inherent in foam-like epithelial geometry. This instability can be evoked by an increase in either apical junctional tension or planar cell density, regulated by various cell behaviors such as actomyosin contractility^19–24^, cadherin adhesion^58–60^, and cell proliferation^25–27^. The instability can also be triggered by a decrease in the number of cells adjacent to a delaminating cell, mediated by the formation of cell scutoid geometry. Overall, these findings demonstrate that epithelial stratification can be understood as a structural transition governed by foam mechanics. This analogy could be applied to general epithelial stratification, including those occurring during embryogenesis and carcinogenesis.

Here, “foam mechanics” refers to the geometric and mechanical analogy between epithelial cells and foam-like packed objects, where cell shapes are constrained by volume, surface tension, and interfacial tension. Unlike passive foams, epithelial tissues are active materials, but in our model their active processes, such as cytoskeletal contraction, adhesion, and proliferation, are coarse-grained into effective mechanical parameters held constant within each simulation. This approximation allows us to extract the foam-like geometric instability underlying delamination and stratification, while local, state-dependent regulation of active mechanical processes remains an important direction for future work.

The stratified regime in our 3D model may be conceptually related to the collapsed-tiling regime described in 2D vertex models^46^. In 2D, apical packing instability can only be resolved within the epithelial plane, whereas in 3D cells can also deform and rearrange along the apicobasal axis, leading to delamination and stratification. Therefore, although the two regimes appear qualitatively similar, they occupy different regions in the *λ* and *Γ* parameter space. This additional geometric freedom may explain why the corresponding regimes are not quantitatively aligned in parameter space. Relatedly, monolayer tissues generally remained relatively flat, whereas stratified tissues in our simulations often exhibited increased surface curvature and wrinkling (Supplementary Fig. 4a–e). These observations suggest that collapsed tiling in 2D may represent a planar signature of an instability that can manifest as stratification and tissue-scale deformation in 3D, although future studies will be needed to quantitatively compare these regimes and determine the causal role of curvature.

The process of nucleation and growth is a well-established concept in solid matter physics. A previous study demonstrated that, in epithelial cell clustering, the transition from dispersion to aggregated states proceeds via a phase transition driven by and growth processes^61^. In the present study, we examine epithelial stratification from the perspective of nucleation-and-growth processes. Traditionally, crystallization of materials, such as water and metals, is driven by nucleation and growth, typically induced by an increase in material density^62^. Our research demonstrated that epithelial stratification shares these characteristics (Figs. 1 and 2), highlighting their similarities with nucleation-and-growth processes in a wide range of biological phenomena, including embryogenesis and carcinogenesis (Fig. 5). Based on this analogy, we can apply methods used in the physical understanding of nucleation and growth to investigate epithelial stratification.

This viewpoint is useful because it describes epithelial stratification not simply as a gradual accumulation of delaminating cells, but as a structural transition involving the initiation and expansion of multilayer domains. It therefore allows us to separately evaluate the onset of local delamination events and the subsequent growth of multilayer regions, and to identify which cellular behaviors control each step. For instance, using the JMAK model^42^, we evaluated whether nucleation or growth drives each stratification process. Specifically, frequent growth was observed in MDCK cell culture (Fig. 2c, d). This analysis, together with existing methods for nucleation and growth, may help deepen our understanding of cell behaviors underlying a wide range of phenomena involving epithelial stratification, including epithelial morphogenesis during development^1–9^, teratogenic diseases caused by stratification failure, such as lissencephaly and neuronal migration disorders^63^, and malignant transformation initiated by stratification^13^.

Other physical behaviors may also contribute to the transition from a monolayer to a multilayer structure in epithelia. One possibility is active cell migration^64^, which has been observed in wound healing^65^ and morphogenetic processes^66^. Cells actively move and rearrange in response to internal or external cues, potentially leading to local overcrowding and cell delamination. Another candidate is cell sorting^67^, driven by differential adhesion or cortical tension, which can cause spatial segregation of cell populations and potentially result in multilayer structures, particularly when combined with variations in cell type or mechanical properties. Moreover, several biological behaviors may also contribute to this transition, such as the orientation of cell division directions^14–16^, differences in proliferation rates^15^, and signaling patterns^68,69^. Topological defects may also influence the spatial localization of nucleation within the monolayer. Previous studies have shown that such defects can promote the initiation of stratification by locally altering cell geometry and mechanical stress^32,33^. Thus, topological defects may act as preferential nucleation sites, and incorporating these spatial cues into the present framework will be important for understanding how nucleation sites are selected in developing and pathological epithelia. These alternative behaviors highlight the complexity of epithelial morphogenesis and suggest that multiple, possibly concurrent, mechanisms may underlie stratification depending on the biological context.

While our model revealed that an increase in planar cell density facilitates epithelial stratification, high density alone does not always lead to stratification. Our model also predicted that whether stratification occurs depends not only on cell density but also on other parameters, such as apical junctional tension. Consistently, the *Drosophila* imaginal disc epithelium, a pseudostratified monolayer, maintains its structure despite increased proliferation and crowding^70^. Similarly, larval salivary glands remain as a monolayer even though their cells are large and polyploid^71^. These observations suggest that additional mechanical factors, such as apical junctional tension, cell–ECM interactions, and cell-nuclear interactions, play critical roles in determining epithelial layer architecture. The deformability of individual cells, as observed in pseudostratified tissues, may also be a key determinant. Models that incorporate more flexible representations of cell shape^72,73^ may help address these complexities and further refine our understanding of stratification mechanisms.

Recent studies have revealed that scutoids emerge frequently in epithelia with high cell density or high curvature or after proliferation events^51–53^. Our model and experiments revealed that scutoids also appear during epithelial stratification. However, scutoid formation does not always induce stratification. Consistently, tissues such as the salivary gland^51^ and sea star blastulae^53^ exhibit scutoid-rich architectures while maintaining a monolayer. These findings suggest that scutoids represent an intermediate state of cell rearrangement, enabling topological transitions through the exchange of cell-cell contacts within the epithelial plane. While this study provides a conceptual understanding of epithelial stratification, it is important to acknowledge its limitations. Through our experiments, the nucleation-growth process was observed in MDCK cell cultures, skin development, and cancer cell invasion, suggesting the applicability of this concept to a wide range of epithelial stratification. On the other hand, the contribution of mechanical instability to cell stratification in biological systems remains only partially understood. The proposed theoretical model, exhibiting mechanical instability, showed good agreement with perturbative experiments in MDCK cell cultures as well as previous studies, thereby supporting its applicability. Nevertheless, clarifying its role in embryogenesis and carcinogenesis will require further investigation. Additionally, our model simplifies the problem of energy minimization for a fixed number of cell populations with uniform properties. The model also adopts stress-free boundary conditions; however, in vivo epithelial tissues are subject to various external forces, including confinement by surrounding tissues and interactions with the substrate and ECM. These factors may significantly influence cell delamination and stratification^74,75^. This simplification allows clarification of the fundamental understanding, whereas the roles of more complex cell behaviors remain unclear, such as cell proliferation, cell motility, and the diversity in cell properties caused by differentiation, all of which could play significant roles in regulating stratification in broader biological scenarios^14–16,69,71,76–78^. As such, the relationship between our experiments and simulations remains primarily qualitative. This study marks the beginning of a multiscale physical exploration aimed at understanding the entire stratification process. Incorporation of these additional cellular behaviors in future research could lead to a more quantitative understanding of critical biological processes such as embryonic development and cancer progression.

## Methods

### MDCK cell line

Madin-Darby canine kidney (MDCK, type I) cells stably expressing mCherry-CAAX were provided by Dr. Kajiwara (Osaka University, Osaka). The cells were cultured in Dulbecco’s Modified Eagle’s medium (DMEM) (FUJIFILM Wako, 043-30085) supplemented with 10% fetal bovine serum (FBS, Gibco, 26140079 or NICHIREI, 175012) and 1% penicillin-streptomycin (FUJIFILM Wako, 168-23191 or Sigma-Aldrich, P4333-100ML). Cultures were maintained in a 5% CO_2_ incubator at 37 °C.

### MDCK cell culture to observe epithelial stratification

A 35 mm glass-bottom imaging dish (Matsunami) was coated with 30 µL of Cellmatrix Type I-A (Nitta Gelatin, 637-00653). A cell suspension containing 1.8 × 10^5^ cells in 250 µL of DMEM supplemented with 10% FBS was added to the dish and incubated. After 4 hours, 2 mL of the medium was added. The dish was incubated until 60 hours after addition of the cell suspension. To inhibit proliferation, MDCK cells were treated with aphidicolin (1 µM final concentration; prepared from a 10 mM stock solution in dimethyl sulfoxide; Sigma-Aldrich, A0781) at 6 or 24 hours after addition of the cell suspension.

### Immunostaining of MDCK epithelium

Cells were fixed in 4% paraformaldehyde (PFA) (FUJIFILM Wako, 163-20145) in phosphate-buffered saline (PBS) (Nacalai tesque, 11480-35) for 30 minutes at room temperature (RT), followed by two washes with PBS. The cells were permeabilized with 0.5% Triton X-100 (MP Biomedicals, 11456631) in PBS for 2 minutes. After three washes with PBS, the samples were blocked with 4% bovine serum albumin (BSA) (FUJIFILM Wako, 015-27053)/PBS for 10 minutes and incubated for 1.5 hours with rabbit anti-ZO-1 (1:100, Invitrogen, 61-7300). After washing with PBS, the samples were incubated for 1.5 hours with Goat anti-Rabbit IgG 488 H&L antibody (1:100, Abcam, ab150077), Phalloidin-iFluor 647 Conjugate (1:1000, Cayman, 20555), and Hoechst 33258 (1:1000, FUJIFILM Wako, 343-07961). Following three washes with PBS, the samples were mounted in 50% glycerol in PBS. Fluorescent images were acquired using a BZ-X810 microscope (Keyence).

### Observation and quantification of MDCK epithelium

Z-stack imaging was performed at each time point using a BZ-X810 microscope (Keyence) equipped with a 60× (NA 0.45) objective and an LSM800 microscope (Zeiss) equipped with a 25× (NA 0.8) objective. Images were captured using the 2× zoom function with a step size of 0.4–2 µm. Five images were obtained from the center to the periphery of the dish at each time point.

The stratification ratio, *ξ*, was calculated using Eq. (1). For this calculation, cells were categorized into four types: cells with both apical and basal surfaces, cells with either apical or basal surfaces, and cells with neither apical nor basal surfaces (Fig. 1a). The presence of apical and basal surfaces was manually identified using an Advanced Observation Module (BZH4XD, Keyence), based on the mCherry-CAAX intensity. *ξ* was calculated at every time point and fitted with the JMAK model in Eq. (3) using a nonlinear least squares method to obtain the parameters *k*, *ν*, and ${t}_{0}$. Moreover, delaminated cells were classified as isolated and clustered based on the presence of adjacent delaminated cells using Fiji software.

### Quantification of planar cell density

Planar cell density, ρ, was defined as the number of cells per unit projected area in both experiments and simulations. In MDCK experiments, cells were counted using mCherry-CAAX fluorescence. To account for cells intersecting the image boundary, the number of cells was calculated as ${N}_{{{{\rm{c}}}}}+{N}_{{{{\rm{e}}}}}/2$, where ${N}_{{{{\rm{c}}}}}$ is the number of cells fully contained within an image and ${N}_{{{{\rm{e}}}}}$ is the number of cells intersecting the image boundary. ρ was then calculated by dividing this cell number by the total image area. To analyze the relationship between ξ and ρ, both quantities were calculated for each individual image. In simulations, where periodic boundary conditions were imposed, ρ was calculated as ${N}_{{{{\rm{t}}}}}/({L}_{x}{L}_{y})$, where ${N}_{{{{\rm{t}}}}}$ is the total number of cells and ${L}_{x}$ and ${L}_{y}$ are the system sizes in the x- and y-directions, respectively.

### Observation and visualization of single MDCK cell shapes

Time-lapse imaging was performed using an FV 3000 confocal microscope (Olympus) equipped with a GaAsP detector (FV31-HSD, Evident) and a dichroic mirror (U-FBNA, Olympus) in a humidified 5% CO_2_ atmosphere at 37 °C. Images were captured with a step size of 0.43 for 6 hours, at 10-minute intervals. Using the mCherry-CAAX intensity, individual cells were manually masked using Fiji and Drishti v. 3.2. The masked images were visualized using ParaView (Kitware Inc.) and Imaris viewer (Bitplane).

### Immunostaining and observation of mouse embryonic skin

To prepare mouse embryonic skin tissue samples, pregnant Jcl:ICR mice (CLEA Japan, Inc.) were euthanized by cervical dislocation, and embryos at E9.5, 11.5, and 13.5 were collected. The head, tail, limbs, and internal organs were removed, leaving the skin tissue, which was fixed with 4% PFA in PBS for 1 hour at 4 °C. Following fixation, the tissues were washed with PBS and blocked with 1% BSA/PBS for 1 hour at 4 °C. Nuclei were stained with DAPI (1:1000, Dojindo, D523), and plasma membranes were labeled with wheat germ agglutinin (WGA) (1:200, Invitrogen, W32466) for 2 hours at RT. The tissues were then washed with PBS and immersed in 60% 2,2’-thiodiethanol (FUJIFILM; 201-04193) in PBS for 1 hour at RT to enhance optical clarity. Fluorescence signals were captured with a TCS SP8 X confocal microscope (Leica) with a 40× oil immersion objective lens (Leica, HC PL APO 40×/1.30 Oil CS2) and LAS X software (version [BETA] 3.5.7.23723).

### Quantification of mouse embryonic skin

The stratification ratio, *ξ*, was calculated using Eq. (1). For this calculation, we counted the total number of cells constituting epidermis in 3D stacked images based on both DAPI and WGA signals using LAS X. Delaminated cells in skin tissue were defined as cells without apical or basal surfaces, and the number of cells was manually counted. Basal surfaces were defined as having high area fluorescence intensity of the WGA signal. Seven samples were randomly selected for each stage.

### Intestinal cancer cell line

Intestinal cancer cells were provided by Dr. Oshima (Kanazawa University, Kanazawa). The cells were originally established from small intestinal tumors of mouse models carrying mutations in the colorectal cancer driver genes *Apc* (A), *Kras* (K), *Tgfbr2* (T), *Trp53* (P), and *Fbxw7* (F) in intestinal epithelial cells^56^. These cells reflect in vivo malignant behaviors, serving as a realistic model to study cancer progression and transformation.

### Intestinal cancer organoid culture

Intestinal cancer cells were enzymatically or mechanically dissociated into either single cells or small aggregates and then cultured at 37 °C in a 48-well dish (Thermo Fisher Scientific, 50687) embedded in 30 µL of Matrigel (Corning, 354230) and surrounded by 300 µL of organoid culture medium^79^. The media contained Advanced DMEM/F-12 medium (Gibco, 12634-010), supplemented with 1% GlutaMAX^TM^ (Gibco, 35050061), 1% N-2 (Gibco, 17502-048), 1% 1 M HEPES buffer solution (FUJIFILM Wako, WK34601373), 1% Penicillin-Streptomycin, 2% B-27 supplement (Gibco, 17504044), and 0.1% 1 mM N-acetylcysteine (Sigma-Aldrich, A91650). Organoids were passaged every 2–3 days for maintenance by resuspending them while embedded in fresh Collagen gel with media in a new well of a culture dish.

### Malignant phenotype induction of intestinal cancer organoids

Intestinal organoids were passaged into a Collagen I matrix (FUJIFILM Wako, 638-00781) using the organoid culture medium described above^57^. After 24 hours of incubation at 37 °C, the medium was replaced with fresh medium with an additional 20 ng mL^−1^ of the induction factor activin A (Cosmobio, 800-01).

### Intestinal cancer organoid immunostaining and observation

Organoids were fixed by immersion in 4% PFA at RT for 30 minutes. Samples were then washed three times in PBS before permeabilization and blocking. Permeabilization was a two-step process starting with immersion in 0.5% Triton X-100 in PBS for 30 minutes at 4 °C. Next, samples were permeabilized in PBS containing 0.1% BSA, 0.2% Triton X-100, and 0.05% Tween20 (FUJIFILM Wako, 166-21213) at RT for a minimum of 2 hours. The samples were blocked by incubation in 1% BSA solution for 1 hour at RT. They were then immunostained overnight at 4 °C with Phalloidin-iFluor 647 Conjugate (1:200), Hoechst 33258 (1:300), and rabbit anti-ZO-1 (1:100) diluted in 1% BSA. Goat anti-Rabbit IgG 488 H&L antibody (1:200) incubation was performed under the same conditions as for primary antibodies after permeabilization in 0.1% BSA, 0.2% Triton X-100, and 0.05% Tween20 buffer at RT for a minimum of 2 hours. Following immunofluorescence assays, fluorescent imaging of samples was performed using a confocal microscope (FV3000, Olympus) equipped with a 20× objective lens (20x-UPlanXApo, Olympus). Three-dimensional projections of images were reconstructed using ImageJ for analysis.

### Intestinal cancer organoid stratification ratio calculation

The stratification ratio, *ξ*, was calculated using Eq. (1). The total number of cells constituting each organoid in 3D stacked images was counted based on both F-actin and Hoechst signals using Fiji software. Delaminated cells in the organoids were defined as cells lacking either apical or basal surface, similar to the definition used for MDCK cells. These cells were manually counted based on ZO-1 and phalloidin immunostaining. For the control group, five organoids were randomly selected from four separate culture dishes. For the activin-treated group, more than ten organoids were selected from more than three dishes.

### 3D modeling of epithelial stratification

We employed a 3D vertex model to calculate the dynamics of multiple cells comprising an epithelial sheet in 3D space. The dimensions of the system box were defined as $0\le x < {L}_{x}$, $0\le y < {L}_{y}$, with z extending infinitely in both directions (−∞ < *z* < ∞) in the xyz coordinates. We applied periodic boundary conditions at x=0, ${L}_{x}$, y=0, and ${L}_{y}$. Within this box, an epithelial sheet, consisting of ${N}_{{{{\rm{t}}}}}$ cells, was arranged in the *xy* plane.

Each cell within the epithelium was modeled as a polyhedron, with shared vertices between adjacent cells. Therefore, the shape and arrangement of cells in the epithelium were described by the positions of the vertices of the polyhedrons and the topological connections among these vertices. The epithelial polarity of each cell surface was defined based on this topology. The top and bottom surfaces of the sheet along the *z* axis were designated as the apical and basal surfaces, respectively, for each cell. Boundaries between adjacent cells were defined as lateral surfaces for each cell.

Cell movements are described by changes in the positions of the vertices. The time evolution of the position of the *i*th vertex, denoted by ${{{{\bf{r}}}}}_{i}$, is described by

4 $${\eta }_{i}\left(\frac{d{{{{\bf{r}}}}}_{i}}{{dt}}-{{{{\bf{v}}}}}_{i}\right)=-{{{{\boldsymbol{\nabla }}}}}_{i}U.$$

The left-hand side of Eq. (4) represents the viscous force acting on the *i*th vertex. Here, *η*_*i*_ denotes the friction coefficient for this vertex, which is calculated as the sum of the friction coefficients from the adjacent cells, expressed as ${\sum }_{\alpha (i)}^{{{{\rm{cell}}}}}{\eta }_{{{{\rm{c}}}}}$ where *η*_*c*_ represents the friction coefficient between cells. The local velocity vector at the *i*th vertex, denoted by **v**_*i*_, in Eq. (4) is defined as the average velocity of the surrounding cells:${\sum }_{\alpha (i)}^{{{{\rm{cell}}}}}{{{{\bf{v}}}}}_{{\alpha }}/{{{{\rm{\sum }}}}}_{\alpha (i)}^{{{{\rm{cell}}}}}$, where ${{{{\bf{v}}}}}_{\alpha }$ is the velocity of the *α*th cell. **v**_α_ is calculated as the average velocity of its constituent vertices. This formulation ensures the balance of the total viscous force within each cell. The right-hand side of Eq. (4) represents the mechanical force acting on the *i*th vertex, which is derived from the effective energy of the system as described in Eq. (2).

During epithelial stratification, cell configurations were rearranged in 3D space. In our model, this cell rearrangement is described by introducing [H]-[I] operations^34^. When individual edges in the network occasionally shrink to meet or retract from neighboring cells, the topological network is reconnected. These [H]-[I] operations differ from the well-known T1 transformation commonly used in 2D vertex models but express several changes in 3D cell configurations, including the rearrangement of cells within the plane of the epithelium, stacking of cells vertically on the plane, and hole formation within the epithelial sheet.

### Stress-free periodic boundary conditions

During the simulation, we ensured that the axial stress in the *xy* plane was approximately free by affinely deforming the system box. This deformation is described by

5 $$\mu \frac{d{L}_{i}}{{dt}}=-\frac{{V}_{{{{\rm{t}}}}}}{{L}_{i}}{\sigma }_{{ii}}.$$

In this equation, the index *i* takes *x* and *y*, representing the respective axes. Here, ${V}_{{{{\rm{t}}}}}$ denotes the total volume of the cells, calculated as ${V}_{{{{\rm{t}}}}}={\sum }_{\alpha }^{{{{\rm{cell}}}}}{V}_{\alpha }$. The constant *μ* is defined as the friction coefficient of the epithelial sheet to its surroundings. ${\sigma }_{{ii}}$ represents the normal component of the stress tensor in the *i*th axis.

The stress tensor of the system, denoted by ${\sigma }_{{ij}}$, is calculated by

6 $${\sigma }_{{ij}}=\frac{{\sum }_{\alpha }^{{{{\rm{cell}}}}}{\sigma }_{{ij}}^{\alpha }{V}_{\alpha }}{{V}_{{{{\rm{t}}}}}}.$$

In this equation, ${\sigma }_{{ij}}^{\alpha }$ represents the stress tensor of the *α*th cell. We define ${\sigma }_{{ij}}^{\alpha }$ by

7 $${\sigma }_{{ij}}^{\alpha }=\frac{1}{{V}_{\alpha }}\mathop{\sum }_{\beta \left(\alpha \right)}^{{{{\rm{vertex}}}}}{\left({{{{\bf{r}}}}}_{\beta \left(\alpha \right)}\right)}_{j}{\left({{{{\bf{F}}}}}_{\beta \left(\alpha \right)}\right)}_{i}.$$

Here, ()_i_ indicates the *i*th component of a vector. The vector ${{{{\bf{r}}}}}_{\beta \left(\alpha \right)}$ is the distance vector from the center of the *α*th cell to the position of the *β*th vertex. ${{{{\bf{F}}}}}_{\beta \left(\alpha \right)}$ represents the force acting on the *β*th vertex within the *α*th cell, given as ${{{{\bf{F}}}}}_{\beta \left(\alpha \right)}=-d{u}_{\alpha }/d{{{{\bf{r}}}}}_{\beta }$, where ${u}_{\alpha }$ denotes the effective energy of the *α*th cell. The total effective energy *U*, described in Eq. (2), is the sum of the effective energies of individual cells: $U={\sum }_{\alpha }^{{{{\rm{cell}}}}}{u}_{\alpha }$, where the effective energy of each cell is given by

8 $${u}_{\alpha }=\frac{K}{2}{\left(\frac{{V}_{\alpha }}{{V}_{0}}-1\right)}^{2}+\kappa {A}_{\alpha }^{{{{\rm{bl}}}}}+{\varGamma \left({P}_{\alpha }\right)}^{2}+\frac{\lambda }{2}{P}_{\alpha }.$$

### Nondimensionalization and numerical simulations

Physical parameters were nondimensionalized by units: length, *l*, was scaled by ${V}_{0}^{1/3}$, time, *τ*, by $12.5{\eta }_{{{{\rm{c}}}}}{\kappa }^{-1}$, and energy, *ε*, by $50\kappa {V}_{0}^{2/3}$. For the numerical integration of Eq. (4), we employed the first order Euler’s method with a time step of Δt, set to 0.005. Vertex velocities in Eq. (4) were iteratively solved by convergent calculations to ensure that the mean residual error was below the threshold, $R{E}_{{{{\rm{th}}}}}$, set to 0.00001. Topological operations were applied to each edge and trigonal face when related edge lengths were under the threshold, $\Delta {l}_{{{{\rm{th}}}}}$, set to 0.05, at each time interval of $\Delta {t}_{{{{\rm{r}}}}}$, set to 1.0. Details of the numerical implementations are similar to those of our previous studies^34,35,47^.

To determine the local energy minimum for each parameter set, we calculated the relaxation process of vertex positions using Eq. (4). Time in these simulations therefore corresponds to numerical relaxation time rather than direct biological time. Simulations were continued until the average vertex displacement became small, indicating convergence to a mechanically stable state. No additional active dynamics, such as self-propulsion or externally imposed active forces, were included. The initial condition for these simulations was a disordered cell configuration within the monolayer. This condition was obtained by simulating tissue growth. This initial condition was created by proliferation from 100 cells to 401 cells under conditions that maintained the monolayer. In the numerical simulations, five disordered monolayer sheets created with different seed values were used as initial conditions. Numerical simulation data were averaged using these five data sets.

### Assessment of epithelial structures

The number of layers in the epithelium, ${N}_{{{{\rm{l}}}}}$, is defined as

9 $${N}_{{{{\rm{l}}}}}=\frac{2{N}_{{{{\rm{t}}}}}}{{N}_{{{{\rm{a}}}}}+{N}_{{{{\rm{b}}}}}}.$$

In this equation, ${N}_{{{{\rm{a}}}}}$ is the number of cells with an apical surface, and ${N}_{{{{\rm{b}}}}}$ is the number of cells with a basal surface. ${N}_{{{{\rm{t}}}}}$ is the number of total cells. This formula applies the trapezoidal formula and measures the average tissue height. This value represents the average number of layers in the tissue.

The nucleation ratio, ${\psi }_{{{{\rm{n}}}}}$, and the growth ratio, ${\psi }_{{{{\rm{g}}}}}$, are defined as

10 $${\psi }_{{{{\rm{n}}}}}=\frac{{N}_{{{{\rm{n}}}}}^{{{{\rm{t}}}}}}{{N}_{{{{\rm{t}}}}}},{\psi }_{{{{\rm{g}}}}}=\frac{{N}_{{{{\rm{g}}}}}^{{{{\rm{t}}}}}}{{N}_{{{{\rm{t}}}}}},$$

respectively. In this equation, ${N}_{{{{\rm{n}}}}}^{{{{\rm{t}}}}}$ and ${N}_{{{{\rm{g}}}}}^{{{{\rm{t}}}}}$ represent the total numbers of cells delaminated through the nucleation and growth process, respectively, over the period in which the stratification ratio *ξ* increases from 0 to 0.5. ${N}_{{{{\rm{t}}}}}$ represents the total number of cells, including both delaminated and non-delaminated cells.

### Frequencies of cell delamination in potential nucleation and growth regions

At a given time *t*, the monolayer region is classified into two types of regions. The first is the potential nucleation region, which is composed of cells for which all first-neighboring cells have both apical and basal surfaces. The second is the potential growth region, composed of cells for which the first-neighboring cells include at least one cell that has only an apical or basal surface or neither (Supplementary Fig. 3f).

Let ${N}_{{{{\rm{n}}}}}^{{{{\rm{pot}}}}}\left(t\right)$, and ${N}_{{{{\rm{g}}}}}^{{{{\rm{pot}}}}}\left(t\right)$ denote the number of cells in the potential nucleation and growth regions at time *t*, respectively. ${N}_{{{{\rm{n}}}}}^{{{{\rm{del}}}}}\left(t,t+\Delta t\right)$ and ${N}_{{{{\rm{g}}}}}^{{{{\rm{del}}}}}\left(t,t+\Delta t\right)$ represent the number of cells in each region at time *t* that undergo delamination during the interval [t,t+Δt]. The frequencies of cell delamination in the potential nucleation and growth regions at time *t* are defined as:

11 $${f}_{{{{\rm{n}}}}}^{{{{\rm{del}}}}}\left(t\right)=\frac{{N}_{{{{\rm{n}}}}}^{{{{\rm{del}}}}}\left(t,t+\Delta t\right)}{{N}_{{{{\rm{n}}}}}^{{{{\rm{pot}}}}}\left(t\right)\Delta t},{f}_{{{{\rm{g}}}}}^{{{{\rm{del}}}}}\left(t\right)=\frac{{N}_{{{{\rm{g}}}}}^{{{{\rm{del}}}}}\left(t,t+\Delta t\right)}{{N}_{{{{\rm{g}}}}}^{{{{\rm{pot}}}}}\left(t\right)\Delta t}.$$

To calculate the ratio of these frequencies, we first averaged each frequency over the period while the stratification ratio *ξ* ranged from 0 to 0.25. The ratio was computed using these average values.

### Quantification of apparent local planar cell density

To quantify the apparent local planar cell density in the potential growth region and the potential nucleation region under mechanically stable configurations, we performed simulations using the 3D vertex model in which cell rearrangements were suppressed. Initial conditions were prepared from the mixed layer structure obtained at a stratification ratio of $\xi \approx 0.1$. Starting from these initial conditions, the vertex positions were relaxed according to Eq. (4), while the network topology was fixed by disabling network reconnection. Fixing the network topology was necessary because reconnection would alter local cell adjacency and continuously change the assignment of cells to the two regions, thereby making direct comparison of regional densities difficult.

For the resulting mechanically stable configurations, we quantified the apparent local planar cell density for each cell. The apparent planar cell density of the *α*th cell, ${\rho }_{\alpha }^{{{{\rm{local}}}}}$, was defined as the inverse of the mean of its apical and basal areas:

12 $${\rho }_{\alpha }^{{{{\rm{local}}}}}=\frac{2}{{A}_{\alpha }^{{{{\rm{a}}}}}+{A}_{\alpha }^{{{{\rm{b}}}}}},$$

where ${A}_{\alpha }^{{{{\rm{a}}}}}$ and ${A}_{\alpha }^{{{{\rm{b}}}}}$ denote the apical and basal areas of the *α*th cell, respectively. For each region, the mean apparent planar cell density was calculated by averaging ${\rho }_{\alpha }^{{{{\rm{local}}}}}$ over all cells assigned to that region.

### Hessian analysis and characterization of energy landscape

To evaluate the mechanical stability of cellular structures obtained from simulations using the 3D vertex model, we performed eigenvalue analysis of the energy Hessian. For a cell with *n* verities, the Hessian matrix of *α*th cell ${{{{\bf{H}}}}}^{{{{\rm{\alpha }}}}}\in {{\mathbb{R}}}^{3n\times 3n}$ was defined as the matrix of second-order partial derivatives of the effective energy U with respect to the vertex coordinates of *α*th cell ${x}_{i}^{\alpha }$ and ${x}_{j}^{\alpha }$:

13 $${H}_{{ij}}^{\alpha }=\frac{{\partial }^{2}U}{\partial {x}_{i}^{\alpha }\partial {x}_{j}^{\alpha }}.$$

We performed eigenvalue analysis by solving ${{{{\bf{H}}}}}^{{{{\rm{\alpha }}}}}{{{\boldsymbol{\phi }}}}=e{{{\boldsymbol{\phi }}}}$, where e and ${{{\boldsymbol{\phi }}}}$ represent the eigenvalue and its corresponding eigenvector, respectively. The number of negative eigenvalues was used as a direct metric of instability. By analogy with the index of a stationary point, we refer to this quantity as the index, although the analyzed states were not necessarily stationary points. A non-zero index indicates the presence of unstable directions in the energy landscape and a tendency toward spontaneous conformational transitions.

### Theoretical model of 3D foam geometry and mechanics

To analyze the mechanics of single-cell delamination, we used a theoretical model based on 3D foam geometry^30^. For simplicity, we assumed that the apical and basal surfaces of the epithelial sheet were constrained to remain planar. We considered the movements of a single center cell and its first neighbors within the sheet, while keeping all other neighboring cells fixed in position. Under this approximation, the planar cell density (ρ) implicitly reflects mechanical effects of the surrounding cells.

Specifically, we considered an epithelial monolayer of thickness (*T*), composed of cells with a fixed volume (${V}_{0}$)_._ Under this assumption, the projected area per cell is ${V}_{0}/T$. Therefore, the planar cell density is expressed as $\rho=T/{V}_{0}$, which is consistent with the definition of cell number per unit projected area used in the experiments and simulations. To analyze the mechanisms of single-cell delamination, we modeled the center cell as an *n*-sided regular frustum (*n* ≥ 3) with volume *V*_0_. In this geometry, the center cell has an apical surface, a basal surface, or both and is adjacent to *n* first-neighbor cells with which it shares lateral boundary faces. The *n* first-neighbor cells also share lateral boundary faces and are aligned radially around the center cell.

To describe the local deformation of the cell during delamination, we introduced the taper parameter (d), defined as

14 $$d=\frac{\sqrt{{A}_{{{{\rm{a}}}}}}-\sqrt{{A}_{{{{\rm{b}}}}}}}{\sqrt{{A}_{{{{\rm{a}}}}}}+\sqrt{{A}_{{{{\rm{b}}}}}}},$$

where ${A}_{{{{\rm{a}}}}}$ and ${A}_{{{{\rm{b}}}}}$ denotes apical and basal surface areas, respectively. The set of *n*, *V*_0_, ${A}_{{{{\rm{a}}}}}$, and ${A}_{{{{\rm{b}}}}}$ uniquely determines the shape of the center cell. Moreover, the packing geometry imposes geometric constraints on the physical parameters. The volume of the frustum is calculated as

15 $${V}_{0}=\frac{T({A}_{{{{\rm{a}}}}}+\sqrt{{A}_{{{{\rm{a}}}}}{A}_{{{{\rm{b}}}}}}+{A}_{{{{\rm{b}}}}})}{3}.$$

By solving Eqs. (14) and (15) simultaneously, with $\rho=T/{V}_{0}$, we obtained ${A}_{{{{\rm{a}}}}}$, and ${A}_{{{{\rm{b}}}}}$ as functions of *ρ* and *d* as described by

6 $${A}_{{{{\rm{a}}}}}\left(\rho,d\right)=\frac{3{\left(d+1\right)}^{2}}{\rho \left({d}^{2}+3\right)},$$

17 $${A}_{{{{\rm{b}}}}}\left(\rho,d\right)=\frac{3{\left(d-1\right)}^{2}}{\rho \left({d}^{2}+3\right)}.$$

Additionally, we introduced several geometric parameters, including apical perimeter of the center cell ${P}_{{{{\rm{c}}}}}\left(\ge 0\right)$; total area of the *n* boundary faces between the center cell and its first-neighbor cells ${A}_{{{{\rm{c}}}}}\left(\ge 0\right)$; and total area of the *n* boundary faces between the first-neighbor cells ${A}_{{{{\rm{p}}}}}$.

We modeled the effective energy of the epithelial sheet as follows. First, we assumed a fixed volume for each cell because the cell volume is almost constant during morphological changes in several organisms. Even under the constraints of constant cell volume and planar geometry, the cell-cell boundaries can move in principle, so *d* remained variable. To consider the same situation as a numerical simulation, we introduced the same energy function described in Eq. (2).

The first term in Eq. (2) is negligible because the total volume of each cell is assumed to be constant. However, the other terms are effective, because only the vertices constituting the center cell can move. We expanded the second term around the center cell and first-neighbor cells as $\kappa \left({A}_{{{{\rm{c}}}}}+{A}_{{{{\rm{p}}}}}\right)$. Similarly, we expanded the third term as $\varGamma \left\{1+\left(1/n\right){\left[1-\csc \left(\pi /n\right)\right]}^{2}\right\}$, where $\left\{1+\left(1/n\right){\left[1-\csc \left(\pi /n\right)\right]}^{2}\right\}$ is a correction factor that reflects the effects of the center cell and first-neighbor cells. Finally, we expanded the last term as $\lambda \left[2-\csc \left(\pi /n\right)\right]$, where $\left[2-\csc \left(\pi /n\right)\right]$ is a correction factor that reflects the effects of the center cell and first-neighbor cells. Thus, Eq. (2) can be written as the effective energy of the center cell and first-neighbor cells in the planar sheet as described by

18 $${U}_{{{{\rm{p}}}}}=\kappa \left({A}_{{{{\rm{c}}}}}+{A}_{{{{\rm{p}}}}}\right)+\varGamma \left[1+\frac{1}{n}{\left(1-\csc \left(\frac{\pi }{n}\right)\right)}^{2}\right]{{P}_{{{{\rm{c}}}}}}^{2}+\lambda \left[2-\csc \left(\frac{\pi }{n}\right)\right]{P}_{{{{\rm{c}}}}}.$$

The geometric parameters ${P}_{{{{\rm{c}}}}}$, ${A}_{{{{\rm{c}}}}}$, and ${A}_{{{{\rm{p}}}}}$ in the energy function *U* are described as functions of *n*, *T*, ${A}_{{{{\rm{a}}}}}$, and ${A}_{{{{\rm{b}}}}}$ as described by

19 $${P}_{{{{\rm{c}}}}}\left(n,{A}_{{{{\rm{a}}}}}\right)=2\sqrt{n{A}_{{{{\rm{a}}}}}\tan \left(\frac{\pi }{n}\right)},$$

20 $${A}_{{{{\rm{c}}}}}\left(n,T,{A}_{{{{\rm{a}}}}},{A}_{{{{\rm{b}}}}}\right)=\left(\sqrt{{A}_{{{{\rm{a}}}}}}+\sqrt{{A}_{{{{\rm{b}}}}}}\right)\sqrt{{A}_{{{{\rm{a}}}}}-2\sqrt{{A}_{{{{\rm{a}}}}}{A}_{{{{\rm{b}}}}}}+{A}_{{{{\rm{b}}}}}+{T}^{2}\tan \left(\frac{\pi }{n}\right)},$$

21 $${A}_{{{{\rm{p}}}}}\left(n,T,{A}_{{{{\rm{a}}}}},{A}_{{{{\rm{b}}}}}\right)=C-T\sqrt{\frac{n}{2\sin \left(\frac{2\pi }{n}\right)}}\left(\sqrt{{A}_{{{{\rm{a}}}}}}+\sqrt{{A}_{{{{\rm{b}}}}}}\right).$$

Here, *C* is a constant that represents the total area of the boundary faces between neighbor cells and the cross section in the center cell. Because ${A}_{{{{\rm{p}}}}}$ is incorporated into the energy function in Eq. (2) as a first-order process, *C* is negligible. Using the analytical expressions of ${U}_{{{{\rm{p}}}}}$, we then calculated energy landscapes as functions of *d* under specific *n*, *ρ*, and *λ* values. Physical parameters were nondimensionalized by units: length, *l*, was scaled by ${V}_{0}^{1/3}$, and energy, *ε*, by $50\kappa {V}_{0}^{2/3}$.

### AFM measurement of cell stiffness

For the AFM measurements, we used a JPK Nanowizard 4 (Bruker) equipped as a confocal microscope (Expert line, Abberior). To measure stiffness at cell-cell junctions, we used a 3XC-GG cantilever (OPUS, spring constant 0.1–0.6 N m^−1^). Immediately before measurement, the medium was replaced with DMEM supplemented with 10% serum, 1% penicillin/streptomycin, and 10 mM HEPES. The following parameters were set: setpoint of 2–4 nN, *z* length of 6–8 µm, *z* speed of 100–120 µm s^−1^, and a scan area of either 30 × 30 or 50 × 50 µm with a resolution of 128 × 128 or 256 × 256 pixels. After measurement, fluorescence images were captured using confocal microscopy, similar to the epithelial measurement. After averaging and baseline subtraction, Young’s modulus was calculated using a Hertz model with a triangular pyramid half-cone angle of 35°. The Young’s modulus map was then exported and converted to monochrome. AFM measurements were used to obtain spatial maps of the apparent elastic modulus across the epithelial surface, followed by separate quantification of junctional and non-junctional cell-body regions. Cell-cell junction regions were manually extracted from these maps, and the junctional stiffness at each pixel was obtained using Gwyddion software. Because this apparent modulus reflects not only passive material elasticity but also cortical and junctional tension^50^, the values measured at junctional regions were interpreted as a relative readout of local junctional tension rather than as a direct measurement of a model parameter.

After each AFM measurement, 3D fluorescence images were acquired using a confocal microscope (Expert Line, Abberior) with a 60× lens (NA 0.7). Images were acquired with 560 nm illumination, 10% laser power, and a 10-µs exposure time per pixel. The scan size was 120 × 120 µm with a resolution of 600 × 600 pixels in the *xy* plane and 20–25 µm in the *z* axis with a 1 µm interval. To assess the dependence of epithelial stiffness, the planar cell density was calculated from the 3D fluorescence images. Because the AFM scan area was centered in the fluorescence image, the image was cropped to a size of 60 × 60 µm including the AFM scan area. A total of 19 stiffness maps were obtained from four dishes.

### Statistics and reproducibility

Sample sizes were chosen based on standard practices in cell biology and quantitative image analysis to ensure adequate statistical power; no statistical methods were used to predetermine sample sizes. No data or samples were excluded from the final analysis. All experiments, including representative micrographs and quantitative measurements, were repeated independently at least three times with similar results. For image analysis, we used ImageJ (version 1.53t), Leica Application Suite X (LAS X) (version [BETA] 3.5.7.12723), and custom C + + code. All statistical analyses were performed using Microsoft Excel (Microsoft 365, version 2501) and R (version 4.4.3).

### Reporting summary

Further information on research design is available in the Nature Portfolio Reporting Summary linked to this article.

## Supplementary information

Supplementary Information

Reporting Summary

Transparent Peer Review file

## Source data

Source Data

## Data Availability

Source data are provided with this paper.

## Code availability

The code used for the analyses in this study is available from the corresponding author upon reasonable request.
