# Supplementary material for: Epithelium stratifies via nucleation and growth induced by foam-geometric instability
Source: Nat Commun. 2026 Nov 13;17:11418. doi: 10.1038/s41467-026-78964-1 (PMC13768340; doi:10.1038/s41467-026-78964-1)
Supplement: Supplementary file 1 [file 41467_2026_78964_MOESM1_ESM.pdf]

Supplementary Information for

Epithelium Stratifies via Nucleation and Growth Induced by Foam-Geometric Instability

Shuya Fukamachi[1], Razib Datta[1], Duligengaowa Wuergezhen[2], Takehiko Ichikawa[3], Rei Yagasaki[3], Phoebe Leeaw[1], Makiko Arai[1], Aki Teranishi[1], Shuhei A. Horiguchi[3], Kohei Omachi[2], Aiko Sada[4], Itaru Imayoshi[5,6], Kentaro Kajiwara[7], Tetsuya Hiraiwa[8], Masanobu Oshima[3,9], Takeshi Fukuma[3], Hironobu Fujiwara[2], Satoru Okuda[3,10]*

[1] Graduate School of Frontier Science Initiative, Kanazawa University, Kanazawa 920-1192, Japan

[2] Laboratory for Tissue Microenvironment, RIKEN Center for Biosystems Dynamics Research (BDR), Kobe 650-0047, Japan

[3] Nano Life Science Institute, Kanazawa University, Kanazawa 920-1192, Japan

[4] Division of Skin Regeneration and Aging, Medical Institute of Bioregulation, Kyushu University, Fukuoka 812-8582, Japan

[5] Graduate School of Biostudies, Kyoto University, Kyoto 606-8501, Japan

[6] Institute for Life and Medical Sciences, Kyoto University, Kyoto 606-8501, Japan

[7] Research Institute for Microbial Diseases, Osaka University, Osaka 565-0871, Japan

[8] Institute of Physics, Academia Sinica, Taiwan 115201, Taiwan

[9] Cancer Research Institute, Kanazawa University, Kanazawa 920-1192, Japan

[10] Sapiens Life Sciences, Evolution and Medicine Research Center, Kanazawa 920-1192, Japan

*Corresponding author: satokuda@staff.kanazawa-u.ac.jp (S.O.)

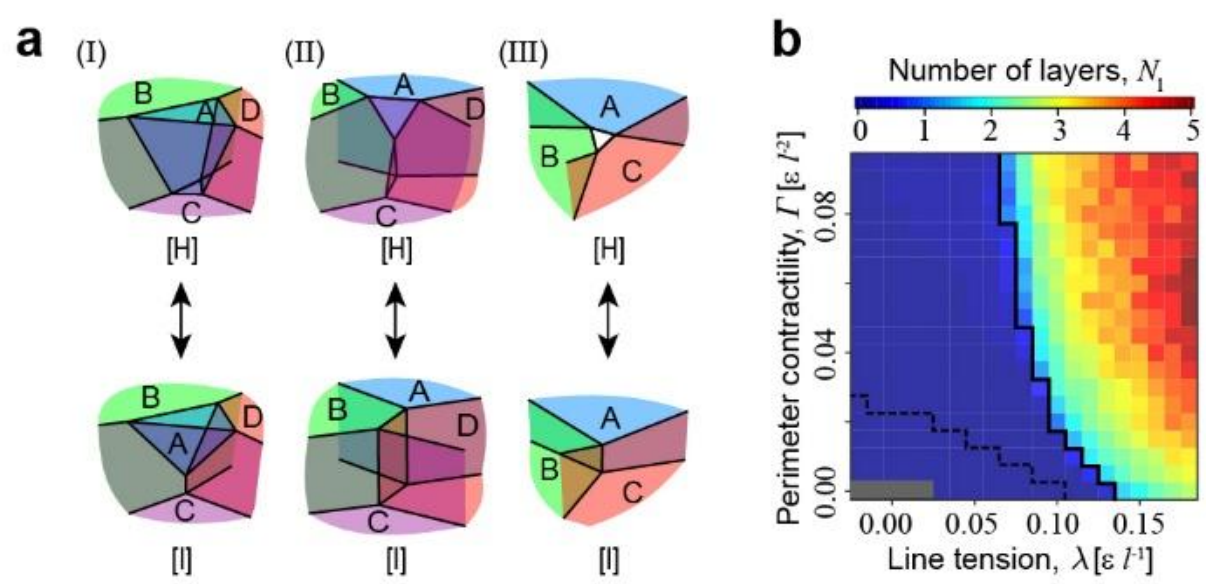

Supplementary Fig. 1. Topological operations and layer number in the 3D vertex model. a Example of topological operations between patterns [H] and [I]. Cell delamination from a monolayer (I), cell rearrangement within the sheet plane (II), and sheet collapse through hole formation (III). **b** Number of layers, N_l, with respect to Γ and λ. In (**b**), solid and dotted lines indicate boundaries among the multilayer ($\xi > 0.5$), mixed layer ($0.5 \geq \xi > 0.0$), and complete monolayer ($\xi = 0.0$). The gray-colored regions indicate the collapsed state. The data in (**b**) indicate the average values calculated from $N = 5$ independent simulation runs with different initial conditions. Source data are provided as a Source Data file.

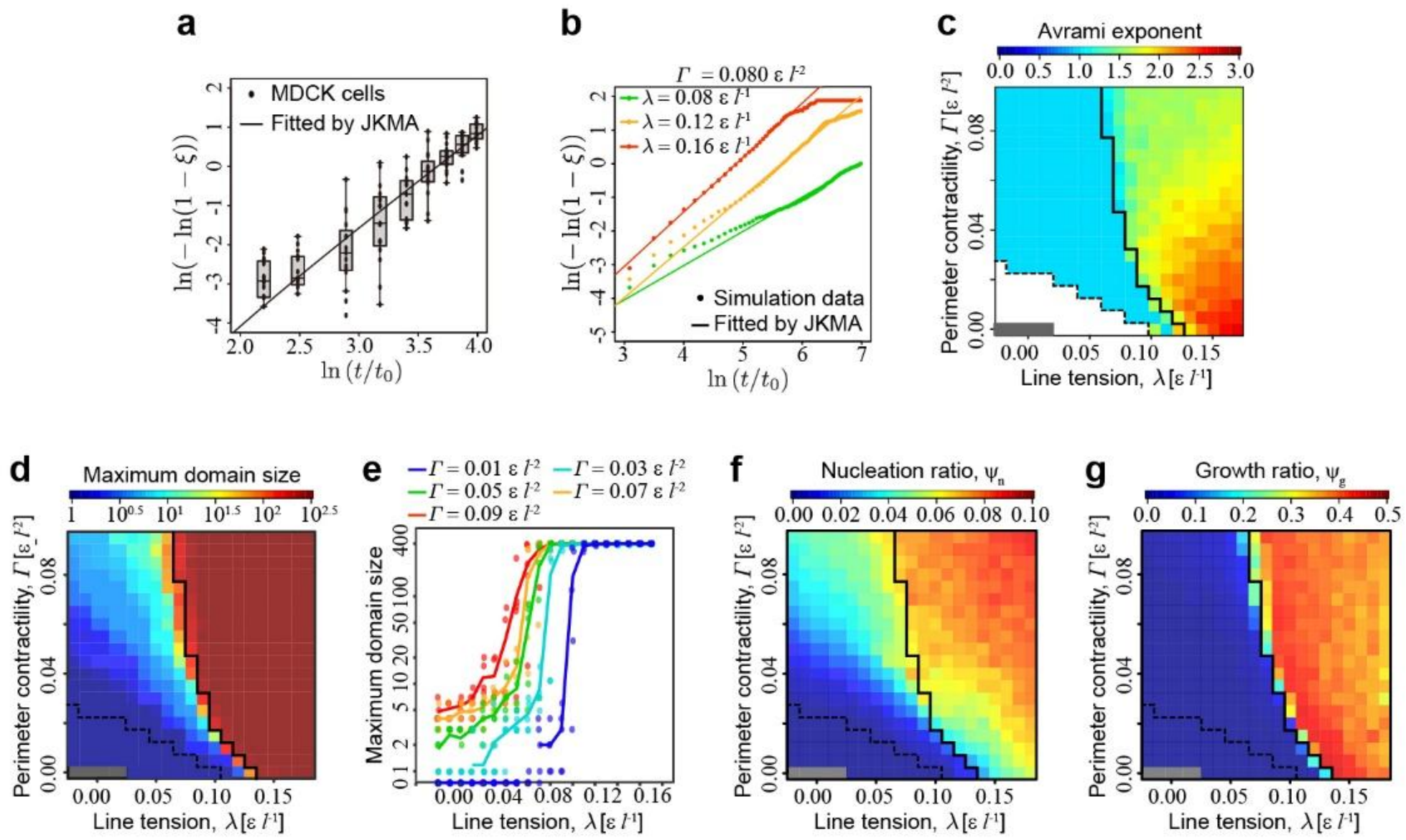

Supplementary Fig. 2. Kinetic analysis of epithelial stratification in experiments and simulations. **a** Avrami plots of the stratification ratio in experiments, ξ, as a function of time, *t*. Solid lines indicate lines fitted by the JMAK model. **b** Avrami plots of the stratification ratio in simulations, ξ, as a function of time, *t*. Dots indicate simulation data. Solid lines indicate lines fitted by the JMAK model. **c** Avrami exponent, ν, with respect to Γ and λ. **d** Maximum domain size over time with respect to Γ and λ. **e** Maximum domain size over time as a function of λ. **f**, **g** Nucleation ratio, ψ_{n}, and growth ratio, ψ_{g}, with respect to Γ and λ. Data in (**a**) represent $n = 15$ fields of view analyzed across $N = 3$ biologically independent cell culture experiments. In (**c**), (**d**), (**f**), and (**g**), solid and dotted lines indicate boundaries among the multilayer ($\xi > 0.5$), mixed layer ($0.5 \geq \xi > 0.0$), and complete monolayer ($\xi = 0.0$). The gray-colored regions indicate the collapsed state. The data in (**c**), (**d**), (**e**), (**f**), and (**g**) indicate the average values calculated from $N = 5$ independent simulation runs with different initial conditions. In (**c**), the white-colored region denotes the monolayer state, for which the Avrami exponent was not calculated. Source data are provided as a Source Data file.

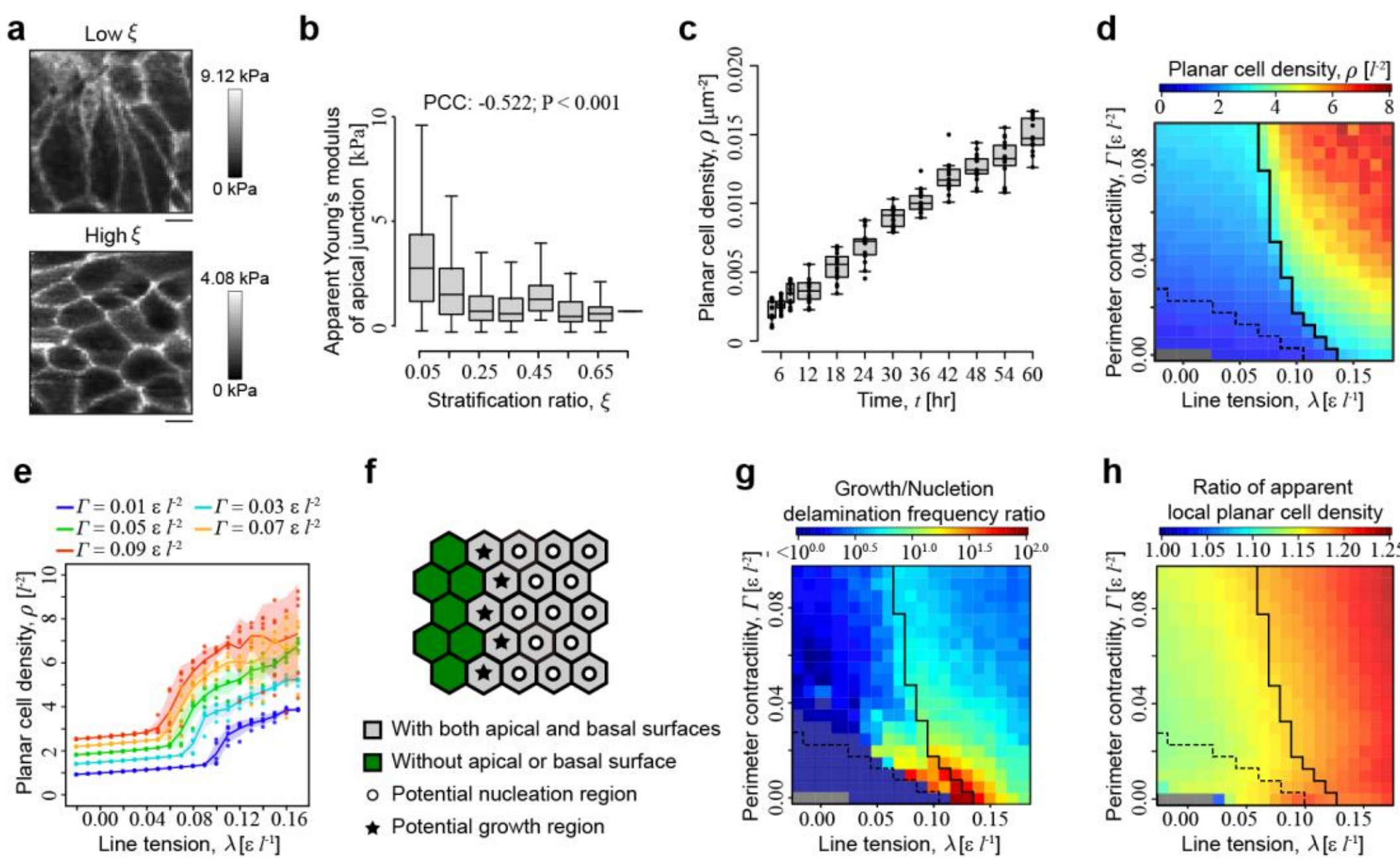

Supplementary Fig. 3. Apical junction mechanics and planar cell density during epithelial stratification. a Apparent Young's modulus maps with high and low ξ. **b** Apparent Young's modulus of apical junction as a function of ξ, obtained from AFM measurements (N = 19). Pearson Correlation Coefficient (PCC) = -0.522, two-sided t (33384) = -111, 95% CI [-0.5298, -0.5141], $P < 1.0 \times 10^{-15}$, n = 33386 pixels. Scale bar: 10 μm. **c** Planar cell density, ρ, as a function of time, t. **d** Planar cell density, ρ, with respect to Γ and λ. **e** Planar cell density, ρ, as a function of λ. **f** Schematic illustration of the potential nucleation and growth regions. **g** Ratio of cell delamination frequency in the potential growth region to that in the potential nucleation region. **h** Ratio of apparent local planar cell density in the potential growth region to that in the potential nucleation region. Apparent local planar cell density was defined as the inverse of the mean planar cell area across the apical and basal surfaces (see Materials and Methods). Data in (**c**) represent n = 15 fields of view analyzed across N = 3 biologically independent cell culture experiments. Box plots in (**b**), and (**c**): center, median; bounds, 25th and 75th percentiles; whiskers, min and max; ***P < 0.001 (two-sided Welch's t-test). In (**b**), individual data points are not shown due to the presence of 33,384 points. In (**b**), the Pearson correlation coefficient (PCC) between the apparent Young's modulus and ξ was calculated; P-values were obtained using Student's t-test for non-correlation. In (**d**), (**g**), and (**h**), solid and dotted lines indicate boundaries among the multilayer ($\xi > 0.5$), mixed layer ($0.5 \geq \xi > 0.0$), and complete monolayer ($\xi = 0.0$). The gray-colored regions indicate the collapsed state. The data in (**d**), (**g**), and (**h**) indicate the average values calculated from N = 5 independent simulation runs with different initial conditions. Band in (**e**) indicates the standard deviation. Source data are provided as a Source Data file.

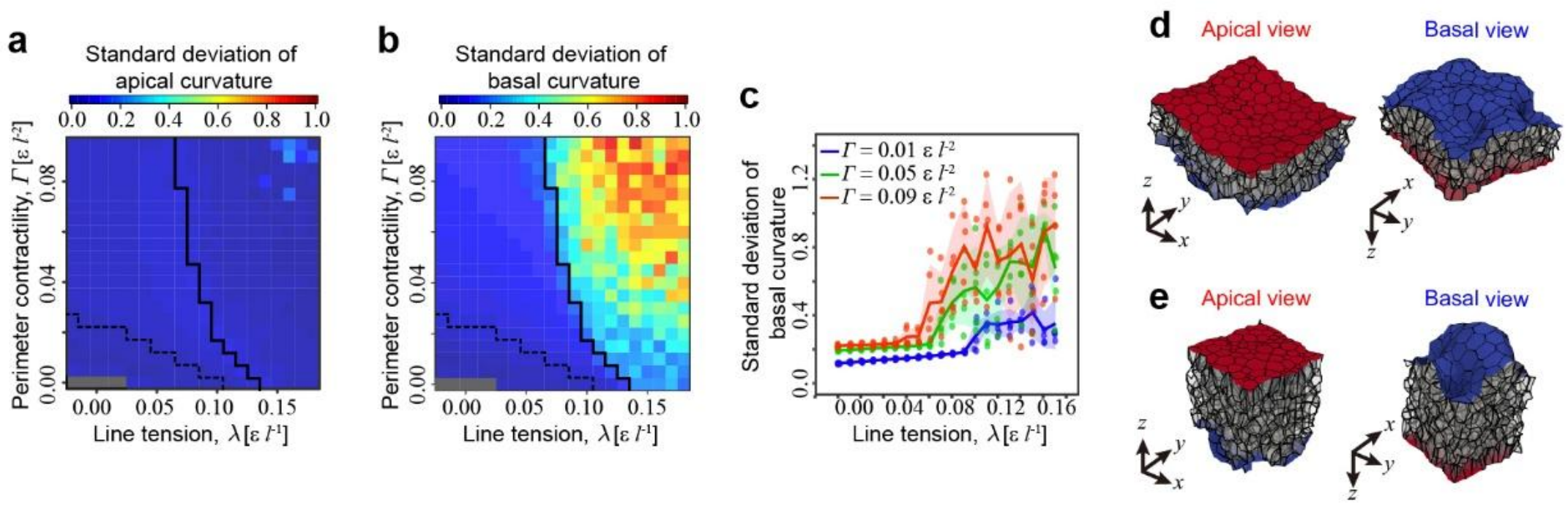

Supplementary Fig. 4. Apical and basal surface curvature in the 3D vertex model. a, **b** Standard deviations of apical and basal curvature, respectively, with respect to Γ and λ. **c** Standard deviation of basal curvature as a function of λ. **d**, **e** Apical and basal surfaces after relaxation. The left and right panels show the apical and basal views in the same tissue, respectively. Surface colors indicate the apical (red), lateral (gray), and basal (blue) surfaces. The parameters used were set to $\Gamma = 0.02\ \varepsilon l^{-2}$, $\lambda = 0.15\ \varepsilon l^{-1}$ (**d**), $\Gamma = 0.08\ \varepsilon l^{-2}$, $\lambda = 0.15\ \varepsilon l^{-1}$ (**e**). In (**a**) and (**b**), solid and dotted lines indicate boundaries among the multilayer ($\xi > 0.5$), mixed layer ($0.5 \geq \xi > 0.0$), and complete monolayer ($\xi = 0.0$). The gray-colored regions indicate the collapsed state. The data in (**a**) and (**b**) indicate the average values calculated from $N = 5$ independent simulation runs with different initial conditions. Source data are provided as a Source Data file.
