# Supplementary material for: Epithelium stratifies via nucleation and growth induced by foam-geometric instability
Source: Nat Commun. 2026 Nov 13;17:11418. doi: 10.1038/s41467-026-78964-1 (PMC13768340; doi:10.1038/s41467-026-78964-1)
Supplement: Supplementary file 2 [file 41467_2026_78964_MOESM2_ESM.pdf]

nature portfolio

Corresponding author(s): Satoru Okuda

Last updated by author(s): Aug 24, 2026

Reporting Summary

Nature Portfolio wishes to improve the reproducibility of the work that we publish. This form provides structure for consistency and transparency in reporting. For further information on Nature Portfolio policies, see our Editorial Policies and the Editorial Policy Checklist.

Statistics

For all statistical analyses, confirm that the following items are present in the figure legend, table legend, main text, or Methods section.

n/a	Confirmed	
[ ]	[x]	The exact sample size (n) for each experimental group/condition, given as a discrete number and unit of measurement
[ ]	[x]	A statement on whether measurements were taken from distinct samples or whether the same sample was measured repeatedly
[ ]	[x]	The statistical test(s) used AND whether they are one- or two-sided<br>*Only common tests should be described solely by name; describe more complex techniques in the Methods section.*
[x]	[ ]	A description of all covariates tested
[ ]	[x]	A description of any assumptions or corrections, such as tests of normality and adjustment for multiple comparisons
[ ]	[x]	A full description of the statistical parameters including central tendency (e.g. means) or other basic estimates (e.g. regression coefficient) AND variation (e.g. standard deviation) or associated estimates of uncertainty (e.g. confidence intervals)
[ ]	[x]	For null hypothesis testing, the test statistic (e.g. F, t, r) with confidence intervals, effect sizes, degrees of freedom and P value noted<br>*Give P values as exact values whenever suitable.*
[x]	[ ]	For Bayesian analysis, information on the choice of priors and Markov chain Monte Carlo settings
[x]	[ ]	For hierarchical and complex designs, identification of the appropriate level for tests and full reporting of outcomes
[ ]	[x]	Estimates of effect sizes (e.g. Cohen's d, Pearson's r), indicating how they were calculated

Our web collection on statistics for biologists contains articles on many of the points above.

Software and code

Policy information about availability of computer code

Data collection	Images were acquired using ZEN 2.6 controlling a confocal microscope (LSM800, Zeiss) and a wide-field microscope (BZ-X810, Keyence).
Data analysis	For image analysis, we used ImageJ (version 1.53t), LAS X (version [BETA] 3.5.7.12723), and custom C++ code. All statistical analyses were performed using Microsoft Excel and R.

For manuscripts utilizing custom algorithms or software that are central to the research but not yet described in published literature, software must be made available to editors and reviewers. We strongly encourage code deposition in a community repository (e.g. GitHub). See the Nature Portfolio guidelines for submitting code & software for further information.

Data

Policy information about availability of data

All manuscripts must include a data availability statement. This statement should provide the following information, where applicable:

- Accession codes, unique identifiers, or web links for publicly available datasets
- A description of any restrictions on data availability
- For clinical datasets or third party data, please ensure that the statement adheres to our policy

Source data are provided with this paper. Additional raw data supporting the findings of this study are available from the corresponding author upon reasonable request.

Research involving human participants, their data, or biological material

Policy information about studies with human participants or human data. See also policy information about sex, gender (identity/presentation), and sexual orientation and race, ethnicity and racism.

Reporting on sex and gender	n/a
Reporting on race, ethnicity, or other socially relevant groupings	n/a
Population characteristics	n/a
Recruitment	n/a
Ethics oversight	n/a

Note that full information on the approval of the study protocol must also be provided in the manuscript.

Field-specific reporting

Please select the one below that is the best fit for your research. If you are not sure, read the appropriate sections before making your selection.

[x] Life sciences   [ ] Behavioural & social sciences   [ ] Ecological, evolutionary & environmental sciences

For a reference copy of the document with all sections, see nature.com/documents/nr-reporting-summary-flat.pdf

Life sciences study design

All studies must disclose on these points even when the disclosure is negative.

Sample size	The sample size was determined by considering the variability of the experimental data. The sample size for each experiment is indicated in the figure legends, and all data are representative of at least three independent experiments.
Data exclusions	Data were excluded only when the corresponding experiment was clearly deemed to have failed, and no other data were removed.
Replication	All replication attempts yielded consistent and reproducible results.
Randomization	In this study, we predefined an imaging criterion in which five images were acquired per time point from the center toward the periphery of the dish.
Blinding	Blinding was not relevant to this study because the data collection and quantitative image analyses were conducted using standardized computational pipelines that strictly follow predefined criteria, eliminating subjective investigator bias during data acquisition and analysis.

Reporting for specific materials, systems and methods

We require information from authors about some types of materials, experimental systems and methods used in many studies. Here, indicate whether each material, system or method listed is relevant to your study. If you are not sure if a list item applies to your research, read the appropriate section before selecting a response.

Materials & experimental systems

n/a	Involved in the study	
[ ]	[x]	Antibodies
[ ]	[x]	Eukaryotic cell lines
[x]	[ ]	Palaeontology and archaeology
[ ]	[x]	Animals and other organisms
[x]	[ ]	Clinical data
[x]	[ ]	Dual use research of concern
[x]	[ ]	Plants

Methods

n/a	Involved in the study	
[x]	[ ]	ChIP-seq
[x]	[ ]	Flow cytometry
[x]	[ ]	MRI-based neuroimaging

Antibodies

Antibodies used	Rabbit anti-ZO-1 polyclonal antibody (Invitrogen, Cat# 61-7300, Lot# 61-7300, polyclonal, dilution 1:100). Goat anti-Rabbit IgG H&L (Alexa Fluor® 488) (Abcam, Cat# ab150077, Lot# ab150077, polyclonal, dilution 1:100–1:200)
Validation	Rabbit anti-ZO-1 antibody (Invitrogen, 61-7300): Validated by the manufacturer and widely cited in literature for immunofluorescence staining of tight junctions in canine (MDCK) and mouse epithelial samples.

Eukaryotic cell lines

Policy information about cell lines and Sex and Gender in Research

Cell line source(s)	Madin-Darby canine kidney (MDCK, type I) cells stably expressing mCherry-CAAX were provided by Dr. Kajiwara (Osaka University, Osaka). Mouse intestine cancer cells were provided by Dr. Oshima (Kanazawa University, Kanazawa).
Authentication	Cell lines were authenticated by distinct morphology using microscopic analysis.
Mycoplasma contamination	No mycoplasma contamination was found.
Commonly misidentified lines (See ICLAC register)	No misidentified cell lines were used in this study.

Animals and other research organisms

Policy information about studies involving animals; ARRIVE guidelines recommended for reporting animal research, and Sex and Gender in Research

Laboratory animals	Pregnant Jcl:ICR mice were obtained from CLEA Japan, Inc.. Primary tissues/cells were harvested from embryos at embryonic days 9.5, 11.5, and 13.5
Wild animals	n/a
Reporting on sex	The sex of the embryos was not determined and was not considered in the study design.
Field-collected samples	n/a
Ethics oversight	All experimental procedures were approved by the RIKEN Center of Biosystem Dynamics Research (BDR) Institutional Animal Care and Use Committee. All mouse care and handling complied with the ethics guidelines of the RIKEN BDR.

Note that full information on the approval of the study protocol must also be provided in the manuscript.

Plants

Seed stocks	n/a
Novel plant genotypes	n/a
Authentication	n/a
