# Supplementary material for: Epithelium stratifies via nucleation and growth induced by foam-geometric instability
Source: Nat Commun. 2026 Nov 13;17:11418. doi: 10.1038/s41467-026-78964-1 (PMC13768340; doi:10.1038/s41467-026-78964-1)
Supplement: Supplementary file 3 [file 41467_2026_78964_MOESM3_ESM.pdf]

nature portfolio

Peer Review File

Epithelium Stratifies via Nucleation and Growth Induced by Foam-Geometric Instability

Corresponding Author: Professor Satoru Okuda

This file contains all reviewer reports in order by version, followed by all author rebuttals in order by version.

Version 0:

Reviewer comments:

Reviewer #1

(Remarks to the Author)
Fukamachi et al. submit an article entitled "Epithelium Stratifies via Nucleation and Growth Induced by Foam-Geometric Instability". The paper reports the transformation of an epithelial MDCK monolayer plated on a gel into a multilayer (stratification) studied by 3D fluorescence microscopy, quantitative imaging, quantification and comparison with a theoretical model (3D vertex model) and numerical simulations. Some mechanical characterisations are performed using AFM. The nucleation and growth process is tested on two other experiment systems – dorsal skin of mouse embryo and malignant transformation of mouse intestinal cancer organoids with similar results and conclusions.
The paper is well presented and well written. Data are convincing from the experimental side and from the theoretical side. To strengthen the impact of this article, I suggest that authors clarify the nature and novelty associated to the identification of a first order phase transition to convince a broad audience in Physics and Biology. I also suggest an additional experiment to test and challenge the core mechanism. Altogether I recommend this excellent paper for publication after these revisions.

Comments :
- The nucleation and growth mechanism is convincing already with images for the MDCK dynamics and the quantification. In this context, the need to go beyond has to be substantiated in the text.
- The signature of a phase transition requires usually the identification of an order parameter and its evolution as a function of temperature – along the theory of Landau. Could the author identify the equivalent for their system if possible and plot them to support the claim of a first order phase transition? Avrami exponent is presented and allows to quantify the kinetics but standard readouts would be needed.
- The identification of a first order phase transition if confirmed is familiar to the physicists. Could the author write in more details the advantages for the general reader?
- The term 'foam' mechanics does not reflect the mechanisms because of the word 'foam' also present in the title and throughout the text. Could the author explain common points and differences between passive foams and active cytoskeleton matter and their impacts on their results ?
- It would be interesting that authors go back to their points presented in the introduction. For example, they could discuss topological defects and the localization for the nucleation phase in the monolayer.
- It would be important to have one experiment preventing of altering nucleation and growth, for example with increased adhesion protein on the gel surfaces.
- How the scutoid shape is robust to changes in parameters ?
- The selection for constant parameters and their influence in Eq. 3 should be discussed – beyond Γ and λ. Also their connections to cellular readouts would be helpful.
- AFM data need to be described in more detail together with the connections to the parameters of the model – see also below. This will support the mechanical instability statement.
- Some typos need to be fixed.

Figures :
Figure 1: could author specify the number of biological repeats and number of monolayer per biological repeats ? The terms cluster is fine but the term disperse can be misleading. Fig1-D model points : can the authors discuss the reliability of the plot ?
Figure 2 : the stratification ratio is the relevant readout. However the author should discuss the sensitivity of parameters to give these results. Intuitive explanation for the mechanism at play could be needed for panel J.

Figure 3 : it would be clearer to have analysis and back-to-back with experimental data. A suggestion is to restructure Figures to streamline the paper.
Figure 4 : why presenting this as a new model? Also, could authors discuss more the AFM experiments relevance with parameters of the model? The connection is not straightforward. In addition, could they do laser ablation to back up the comparison between experiments and theory?
Figure 5 : the same comment as above, it would be important that authors are critical about the robustness of the numerical simulation.
Figure 6 : if possible, it would be nice to have more timepoints like in Fig. 1 to show the nucleation and growth mechanism in these systems – as convincingly as for MDCK cells.

Reviewer #2

(Remarks to the Author)
The manuscript by Fukamachi et al. investigates epithelial delamination and stratification by combining experimental observations, numerical simulations, and theoretical arguments. The authors follow the temporal evolution of an MDCK monolayer transitioning into a multilayered tissue and compare these dynamics with simulations based on a 3D vertex model. They propose that stratification can be understood as a nucleation-and-growth process driven by mechanical instabilities within the tissue. Overall, the study addresses an interesting problem and presents results that could be impactful for the field. However, much of the analysis remains largely descriptive. In my view, the manuscript would benefit from a more quantitative and systematic investigation to substantiate the proposed mechanisms and to move beyond phenomenological observations. As it stands, the mechanistic insights and resulting conclusions appear somewhat superficial. Therefore, I advise a major revision to address key concerns listed below.

1) A major concern relates to the role and usefulness of the mean-field model. The authors show that varying the mean-field parameters can give rise to stable configurations with delaminated cells. However, contrary to previous reports, they also state that the aim of the study is to identify how individual parameters contribute to the stratification process at the tissue level. In this context, it is unclear why the insights obtained from the mean-field model are not translated into measurable or controllable quantities within the 3D vertex model. These quantities could then be directly compared with experimental observations. Establishing such a link would substantially strengthen the mechanistic interpretation of the results. More specifically:

a) The authors compare predictions from the mean-field model with experimentally measured Young's moduli and report a discrepancy between theory and experiment. It is therefore important to clarify how the 3D vertex model behaves in this regard. Based on Fig. 2k, the line tension appears to increase during stratification. Does this indicate that the numerical simulations are likewise inconsistent with the experimental measurements? Furthermore, can Fig. 2k be interpreted in a manner that allows a direct connection to Fig. 4h? If such a connection exists, it remains unclear why apical junctional tension does not emerge as a key driver of stratification, as predicted by the mean-field model. Are there competing mechanisms or compensatory effects in the simulations that could account for the experimental observations?

b) Along similar lines, it would be useful to assess whether the 3D vertex model can reproduce the probability distribution shown in Fig. 4f. Consistency between the simulations and experiments would help validate the relevance of the model.

c) For the analysis as a function of planar density, it would be informative to include vertex-model results analogous to Fig. 4i–j. For which choices of the Hamiltonian parameters does the vertex model recover the observed dependence of the stratification ratio on density? Related to this point, the definition of density itself is currently unclear and inconsistent throughout the manuscript. In one section, density is defined as the inverse of the average apical and basal surface area; in the Methods, it is defined as the number of cells divided by the total image area; and in the mean-field model, density is introduced in Eq. 13 without an explicit definition. I strongly encourage the authors to clarify and unify the definition of density and to provide a clear derivation of Eqs. 13–18, as their origin and assumptions are currently difficult to follow.

2) A central conclusion of the manuscript is that epithelial stratification is triggered by mechanical instabilities induced by apical tension, planar density, or the number of adjacent cells, leading to a nucleation-and-growth–like process. However, the evidence supporting this claim remains largely observational. If the transition to delaminated states is indeed driven by mechanical instabilities, this should be quantitatively demonstrated within the 3D vertex model, for example by analyzing the Hessian of the mechanical energy as a function of time. Do the eigenvalues or normal modes of the Hessian exhibit qualitative or quantitative changes preceding or accompanying stratification? Are such changes correlated with variations in planar density? Performing these analyses would substantially strengthen the mechanistic basis of the proposed interpretation. A similar stability analysis could also be carried out within the mean-field framework. Overall, such assessments would be more informative than the schematic illustration presented in Fig. 4e.

3) In the 3D vertex model, the distinction between analyses of equilibrium configurations obtained after relaxation and analyses of the system's temporal dynamics is currently unclear. For instance, it is not evident how Fig. 2l was generated. The authors state that the parameters Γ and λ were varied over time, but the specific protocol used to implement these temporal changes is not described. Similarly, the origin of the dynamics shown in Fig. 3 is ambiguous. Do these results simply represent the time evolution of the system during energy minimization? Or do they include additional dynamical ingredients, such as self-propelled or active forces? If the former, it is important to clarify whether the reported dynamics correspond to transient states on the way to equilibrium or to a well-defined nonequilibrium process. I strongly encourage the authors to provide more detail on how temporal dynamics are defined and implemented in the vertex-model simulations.

4) Another key conclusion of the manuscript is that epithelial stratification proceeds via a nucleation-and-growth process. Given that this represents one of the central results of the study, a more in-depth and quantitative analysis is needed. In particular, it would be important to characterize the critical nucleus size (or critical radius) associated with the onset of stratification and to examine how this quantity depends on the relevant control parameters. Such an analysis could clarify which factors dominate the growth phase and how these are related to the mechanical instabilities identified in the mean-field model. More specifically, if apical tension and planar density primarily trigger isolated delamination events, what mechanisms promote the growth of delaminated clusters?

5) In their investigation of the 3D vertex model, the authors vary only the parameters Γ and λ. It is unclear why the parameter k, which controls the basolateral surface energy, was not also explored. I would expect this parameter to contribute to the overall mechanical stability of the tissue and, potentially, to the delamination process. It would be helpful clarifying the rationale for fixing k, or alternatively exploring its effect on stratification.

6) As a general final comment, the analyses based on the 3D vertex model remain largely disconnected from the mean-field model. Indeed, the vertex model is used primarily to reproduce experimental observations for selected parameter sets, rather than to systematically test or quantify the proposed mechanisms. As a result, the full potential of the vertex simulations to provide mechanistic insight is not realized. A tighter integration between the mean-field theory, the vertex-model simulations, and the experimental data would be necessary to substantiate the central claims of the work.

Additional minor concerns:

1) If only apical parameters are varied, does this imply that the model can only capture delamination events associated with loss of the apical side? The authors should clarify how different delamination processes are represented, in particular whether the model can account for cells losing either their apical or basal side.

2) The definition of planar density appears inconsistent throughout the manuscript. I recommend that the authors clarify this quantity explicitly and ensure a consistent definition in both the main text and Methods.

3) In the main text, the authors state: “we postulated that all cell-cell boundary surfaces comprised parts of the basolateral surfaces, and the other cell surfaces composing the tissue surface were defined as the apical surfaces.” This sentence is somewhat confusing and is not further clarified in the Methods section. Greater clarity on this point would help the reader interpret the model assumptions.

4) Some figures are not particularly informative, specifically Figure 1f, the simulation snapshots in Figure 2l, and the schematic in Figure 4e. I suggest that the authors either revise these figures to improve clarity or consider removing them to avoid potential confusion.

Reviewer #3

(Remarks to the Author)
The manuscript NCOMMS-25-85464 "Epithelium stratifies via nucleation and growth induced by foam-geometric instability" by Fukamachi et al. reports a comprehensive experimental and theoretical analysis of the transition between single-cell-thick and multilayer epithelial tissues. The authors present experimental results suggesting that stratification can be regarded as nucleation and growth typically associated with, e.g., condensation of gas or crystallization of a liquid. This is an interesting analogy, and the authors provide various pieces of evidence that support it. They also employ a fairly detailed version of the 3D vertex model to simulate stratification, which includes the scutoid shapes. The predictions of the model agree rather nicely with observations. The manuscript is interesting and well-written, the key findings considerably advancing the insight into the mechanics of tissues. The manuscript warrants publication in Nat. Commun. and I suggest that it be accepted after the authors have considered the suggestions listed below.

I only have a few suggestions and questions:

1. In the 3D vertex model, does the tissue remain flat upon stratification? Fig. 2f-h suggest that some deformation takes place. Is this relevant? Does wrinkling present facilitate or suppress stratification?

2. In the model, the apical perimeter elasticity term and the line tension of the apical junctions be combined in a single term of type \Gamma (P-P_0)^2 where P_0 is a preferred cell apical perimeter. It seems that P_0 used in the manuscript is negative so that the two terms would prefer a collapsed apical tiling (cf. Ref. [46], Farhadifar et al. 2007). If I compare Figs. 2i and especially 2j to Fig. 1 from Ref. [46] flipped across the diagonal and somewhat shifted up, it seems to me that the notion

of the collapsed tiling from Ref. [46] may correspond to stratification. Is it so? If yes, would it make sense to discuss it ? Evidently, stratification in a 3D vertex model of a tissue is much more complex but if the collapse of the 2D vertex model can be viewed as an indication of stratification this so far unphysical domain would no longer be unphysical.

If the collapse of the 2D vertex model indeed signals stratification, why the shift in the (contractility, tension) plane mentioned above?

3. Presentation: All figures are quite elaborate and contain many panels. IMHO some panels may be redundant, e.g., Fig. 2j which conveys a very similar message as Fig. 2i. While all graphical material included is relevant, I suggest that the authors consider moving some of it to Supplementary Information so as to better emphasize the essential bits and to reduce reiterating the same message.
Secondly, here and there the language can be improved (e.g., "across broad phenomena" in l. 26 is awkward, "unordered" -> "disordered" in l. 32 and 68). In Figs. 2c, 2i, 2j, 3e, 3f, and 3g, "Perimeter contraction" -> "Perimeter contractility".

Version 1:

Reviewer comments:

Reviewer #1

(Remarks to the Author)
Authors have addressed convincingly the comments with new experiments, new analysis and relevant modifications of the manuscript. I recommend this excellent article for publication.

Reviewer #2

(Remarks to the Author)
The authors have thoroughly addressed all of my concerns and have included new simulations and analyses that significantly improve the clarity and readability of the manuscript. I particularly appreciate their efforts to establish a clearer connection between the mean-field model and the 3D vertex model, as well as their analysis of the mechanical instabilities through computation of the Hessian matrix of the mechanical energy.

I therefore recommend the manuscript for publication in Nature Communications.

Reviewer #3

(Remarks to the Author)
I read the revised version of the manuscript NCOMMS-25-85464 "Epithelium stratifies via nucleation and growth induced by foam-geometric instability" by Fukamachi et al. In the revised main text and supplementary information as well as in their reply to my comments, the authors addressed all issues raised. This interesting, comprehensive, and well-presented manuscript meets all criteria for publication in Nat. Commun. and I suggest that it be accepted.

Open Access This Peer Review File is licensed under a Creative Commons Attribution 4.0 International License, which permits use, sharing, adaptation, distribution and reproduction in any medium or format, as long as you give appropriate credit to the original author(s) and the source, provide a link to the Creative Commons license, and indicate if changes were made.
In cases where reviewers are anonymous, credit should be given to 'Anonymous Referee' and the source.
The images or other third party material in this Peer Review File are included in the article's Creative Commons license, unless indicated otherwise in a credit line to the material. If material is not included in the article's Creative Commons license and your intended use is not permitted by statutory regulation or exceeds the permitted use, you will need to obtain permission directly from the copyright holder.
To view a copy of this license, visit https://creativecommons.org/licenses/by/4.0/

RESPONSE TO REVIEWER #1

Reviewer Comment:

Fukamachi et al. submit an article entitled "Epithelium Stratifies via Nucleation and Growth Induced by Foam-Geometric Instability". The paper reports the transformation of an epithelial MDCK monolayer plated on a gel into a multilayer (stratification) studied by 3D fluorescence microscopy, quantitative imaging, quantification and comparison with a theoretical model (3D vertex model) and numerical simulations. Some mechanical characterisations are performed using AFM. The nucleation and growth process is tested on two other experiment systems – dorsal skin of mouse embryo and malignant transformation of mouse intestinal cancer organoids with similar results and conclusions. The paper is well presented and well written. Data are convincing from the experimental side and from the theoretical side. To strengthen the impact of this article, I suggest that authors clarify the nature and novelty associated to the identification of a first order phase transition to convince a broad audience in Physics and Biology. I also suggest an additional experiment to test and challenge the core mechanism. Altogether I recommend this excellent paper for publication after these revisions.

> **Authors' response:**
> We sincerely thank the reviewer for the careful reading and constructive suggestions, which helped us improve both the clarity and the rigor of the manuscript. We have revised the text and figures to address each point in detail, including additional analyses and new experiments. Revisions and additions are highlighted in **red** in the revised manuscript.

Reviewer Comment 1-1:

The nucleation and growth mechanism is convincing already with images for the MDCK dynamics and the quantification. In this context, the need to go beyond has to be substantiated in the text.

> **Authors' response 1-1:**
> We thank the reviewer for this helpful suggestion. We agree that the time-lapse 3D imaging and quantitative characterization clearly reveal the dynamic transition of the MDCK epithelium from a monolayer to a multilayered structure. We also recognize that the rationale for extending the analysis beyond these observations was not sufficiently explicit in the **Results**. We have therefore

revised the text to clarify that further modeling analysis is needed not simply to reproduce the observed transition, but to examine dynamical and mechanical features that are difficult to access experimentally and to link cell-level behaviors to tissue-scale organization. The following text has been added to the **Results**.

[Results, Page 5]
These findings raise two key questions: how epithelial stratification progresses across the tissue over time, and which cell-level behaviors give rise to this tissue-scale structural transition. To address these questions, we next constructed a cell-based mechanical model linking cellular behaviors to tissue-scale organization and used it together with quantitative observations of epithelial stratification at single-cell resolution to examine its dynamical and mechanical features.

Reviewer Comment 1-2:
The signature of a phase transition requires usually the identification of an order parameter and its evolution as a function of temperature – along the theory of Landau. Could the author identify the equivalent for their system if possible and plot them to support the claim of a first order phase transition? Avrami exponent is presented and allows to quantify the kinetics but standard readouts would be needed.

Authors' response 1-2:
We appreciate this important comment and apologize for the misleading wording in the original manuscript. We agree that a first-order phase transition in the strict thermodynamic/Landau sense would require a well-defined order parameter and a temperature-like control parameter, together with standard thermodynamic readouts such as a discontinuous change in the order parameter. This was not our intention. We have therefore revised the manuscript throughout to avoid this potential misunderstanding: we replaced "phase transition" with "structural transition" where appropriate, and now describe the process as "first-order-like" only in a phenomenological sense, referring to nucleation-and-growth dynamics and hysteretic behavior, rather than as a thermodynamic phase transition. We therefore did not introduce an artificial Landau-type order parameter or temperature-like control variable.

Reviewer Comment 1-3:
The identification of a first order phase transition if confirmed is familiar to the physicists. Could the author write in more details the advantages for the general reader?

Authors' response 1-3:
We thank the reviewer for this helpful suggestion. As clarified in our response to Comment 1-2, we have revised the manuscript to avoid implying a thermodynamic first-order phase transition, and instead describe epithelial stratification from this nucleation-and-growth viewpoint in a phenomenological sense. We have expanded the **Discussion** to clarify the advantage of this structural-transition viewpoint for a broad readership: it provides a simple framework to understand stratification not as a gradual accumulation of delaminating cells, but as a two-step process involving the initiation of local stratified domains and their subsequent expansion. This distinction helps link tissue-scale pattern formation to cell-level behaviors and clarifies which cellular processes control each step.

[Discussion, Page 16]
This viewpoint is useful because it describes epithelial stratification not simply as a gradual accumulation of delaminating cells, but as a structural transition involving the initiation and expansion of multilayer domains. It therefore allows us to separately evaluate the onset of local delamination events and the subsequent growth of multilayer regions, and to identify which cellular behaviors control each step.

Reviewer Comment 1-4:
The term 'foam' mechanics does not reflect the mechanisms because of the word 'foam' also present in the title and throughout the text. Could the author explain common points and differences between passive foams and active cytoskeleton matter and their impacts on their results ?

Authors' response 1-4:
We thank the reviewer for raising this important point. We have revised the **Discussion** to clarify that the term "foam" is used as a geometric and mechanical analogy, rather than to imply that epithelial tissues behave as passive foams. Specifically, we now explain that epithelial cells and passive foams share

constraints arising from cell-volume constraints, surface/interface tensions, and space filling, which can generate foam-like geometric instabilities. Unlike passive foams, epithelial tissues are active materials in which cytoskeletal contraction, adhesion, growth, and proliferation are dynamically regulated by cellular processes. In our model, these active processes are coarse-grained into effective mechanical parameters and cellular dynamics that are held fixed within each simulation. Thus, our results identify a geometry-driven instability that can arise under foam-like packing constraints, while state-dependent regulation of active cellular mechanics remains an important subject for future work.

[Discussion, Page 15]
Here, "foam mechanics" refers to the geometric and mechanical analogy between epithelial cells and foam-like packed objects, where cell shapes are constrained by volume, surface tension, and interfacial tension. Unlike passive foams, epithelial tissues are active materials, but in our model their active processes, such as cytoskeletal contraction, adhesion, and proliferation, are coarse-grained into effective mechanical parameters held constant within each simulation. This approximation allows us to extract the foam-like geometric instability underlying delamination and stratification, while local, state-dependent regulation of active mechanical processes remains an important direction for future work.

Reviewer Comment 1-5:
It would be interesting that authors go back to their points presented in the introduction. For example, they could discuss topological defects and the localization for the nucleation phase in the monolayer.

Authors' response 1-5:
We thank the reviewer for this helpful suggestion. Following the reviewer's comment, we have expanded the **Discussion** to reconnect our findings with the concepts introduced in the Introduction, in particular the possible role of topological defects and local packing heterogeneity in determining where nucleation occurs. We now discuss that such defects may locally alter cell geometry and mechanical stress, thereby serving as preferential sites for the initiation of stratification. We also note that incorporating these spatial cues into the present nucleation-and-growth framework will be important for

understanding how nucleation sites are selected in developing and pathological epithelia.

[Discussion, Page 16]

Topological defects may also influence the spatial localization of nucleation within the monolayer. Previous studies have shown that such defects can promote the initiation of stratification by locally altering cell geometry and mechanical stress[32,33]. Thus, topological defects may act as preferential nucleation sites, and incorporating these spatial cues into the present framework will be important for understanding how nucleation sites are selected in developing and pathological epithelia.

Reviewer Comment 1-6:

It would be important to have one experiment preventing of altering nucleation and growth, for example with increased adhesion protein on the gel surfaces.

Authors' response 1-6:

We thank the reviewer for this important suggestion. To experimentally perturb a key variable in our framework, we performed new MDCK experiments in which the time-dependent increase in planar cell density was suppressed by inhibiting cell proliferation with aphidicolin. Although the reviewer suggested increasing adhesion proteins on the gel surface as one possible perturbation, we focused on planar cell density because our model identifies it as a central control variable for nucleation and growth of stratified domains. Aphidicolin treatment at the time windows corresponding to the nucleation and growth phases suppressed the increase in planar cell density and correspondingly reduced the stratification ratio at matched time points, consistent with the model prediction. These new data are now presented in **Fig. 3i–k** and described in the **Results** and **Methods**.

[Results, Page 11]

To further test this association, we inhibited cell proliferation using aphidicolin at 6 and 24 hours after seeding (Fig. 3i), corresponding to the periods when nucleation and growth predominantly occur, respectively (Fig. 2a, b). Aphidicolin treatment significantly suppressed the increase in ρ compared with the control (Fig. 3j) and consistently suppressed the increase in ξ at the

corresponding time points (Fig. 3k). These results suggest that increasing planar cell density contributes to the progression of epithelial stratification in MDCK cell culture.

[Figure 3i-k & legend]

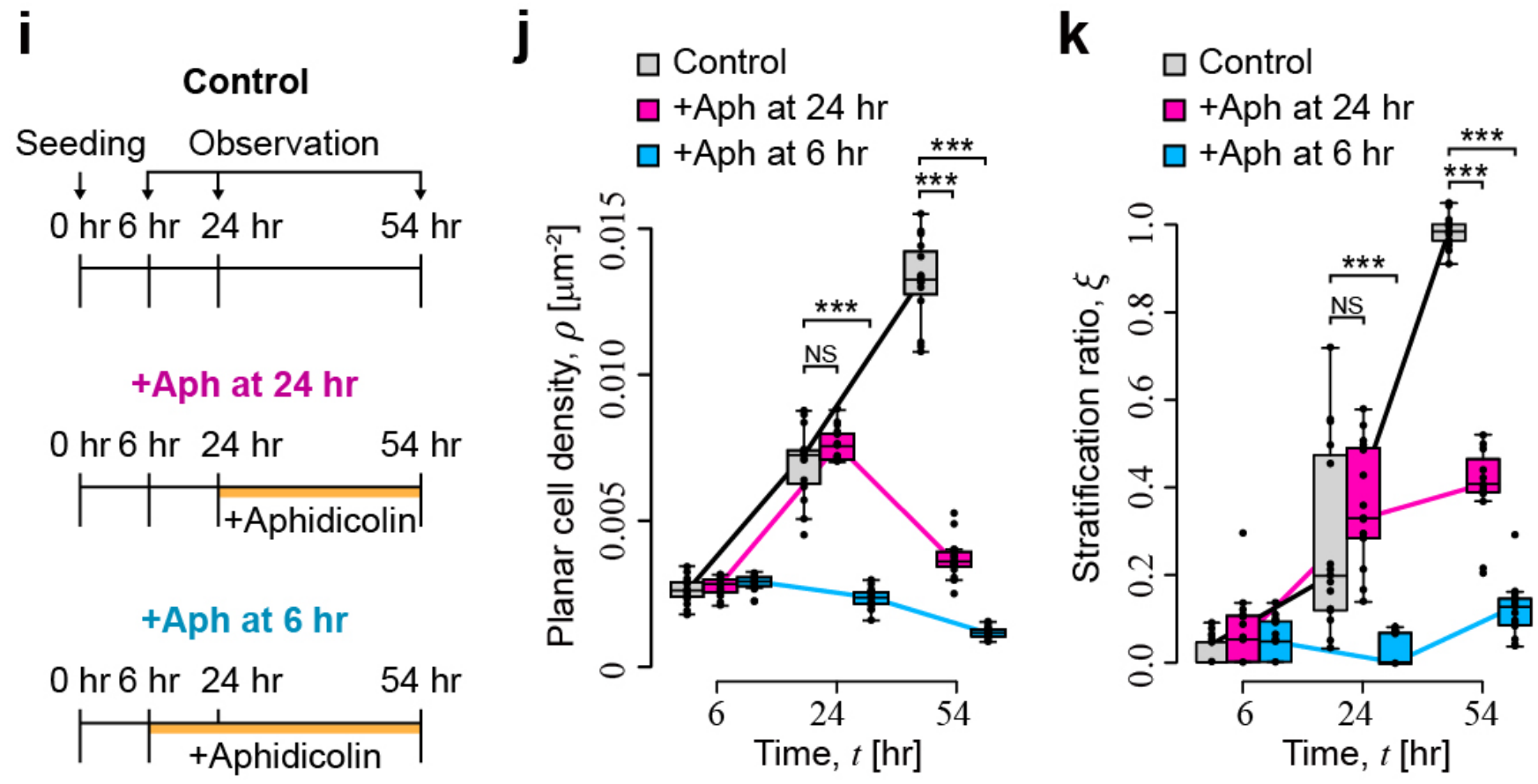

i–**k**, Aphidicolin perturbation assays in MDCK cells. Aphidicolin was added at 6 or 24 h after seeding to suppress the time-dependent increase in planar cell density. **i**, Treatment scheme. **j**, Planar cell density, ρ, as a function of time. **k**, Stratification ratio, ξ, as a function of time. Plots in (**e**), (**h**), (**i**), (**j**), and (**k**) include data from 15 images from three biologically independent samples. Box plots in (**h**), (**j**), and (**k**): center, median; bounds, 25th and 75th percentiles; whiskers, min and max; ***P < 0.001 (two-sided Welch's t-test).

[Methods, Page 18]

To inhibit proliferation, MDCK cells were treated with aphidicolin (1 μM final concentration; prepared from a 10 mM stock solution in dimethyl sulfoxide; Sigma-Aldrich, A0781) at 6 or 24 hours after addition of the cell suspension.

Reviewer Comment 1-7:

How the scutoid shape is robust to changes in parameters ?

Authors' response 1-7:

We thank the reviewer for this important question. To examine the robustness of scutoid formation against parameter changes, we quantified the fraction of cells ever becoming scutoids over a range of Γ and λ. Scutoids were rarely observed

in the complete monolayer regime, but appeared robustly in parameter regions where stratification occurred, particularly in monolayer and multilayer states. These results indicate that scutoid formation is not restricted to a single parameter set but emerges broadly in association with stratification. The new analysis is now shown in **Fig. 4j** and described in the **Results**.

[Results, Page 15]

Moreover, scutoids became more frequent as stratification progressed and were observed in both mixed layer and complete multilayer structures (Fig. 4j). However, within the complete multilayer regime near the monolayer boundary at low Γ, scutoid numbers remained relatively low. This may reflect a regime in which domain growth contributes relatively more than new domain formation (Fig. 2i; Supplementary Figs. 2d, e and 3g).

[Figure 4j & legend]

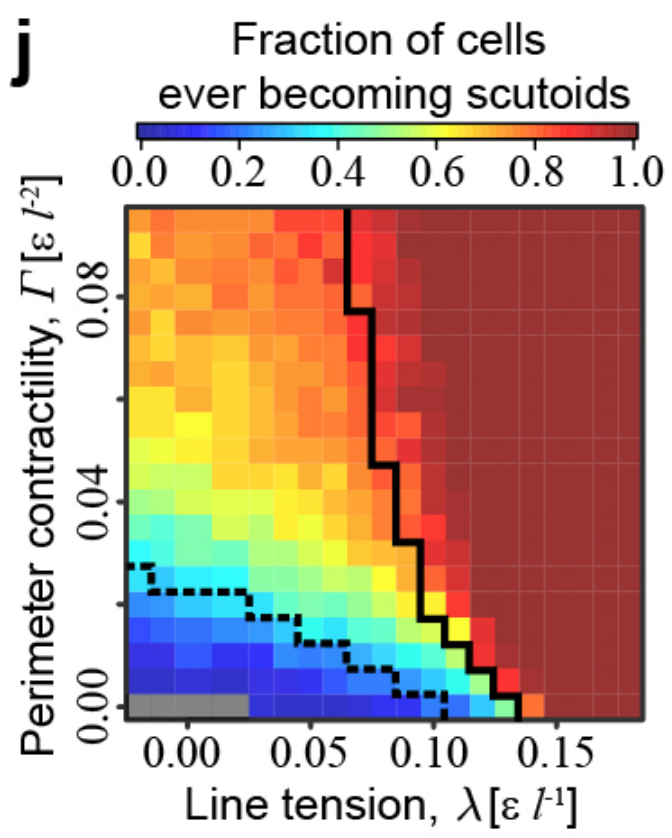

j Fraction of cells ever becoming scutoids with respect to Γ and λ. In (j), solid and dotted lines indicate boundaries among the multilayer ($\xi > 0.5$), mixed layer ($0.5 \geq \xi > 0.0$), and complete monolayer ($\xi = 0.0$). The data in (j) indicate the average values calculated from five independent initial conditions. Gray regions indicate collapsed states.

Reviewer Comment 1-8:

The selection for constant parameters and their influence in Eq. 3 should be discussed – beyond Γ and λ. Also their connections to cellular readouts would be helpful.

Authors' response 1-8:

We thank the reviewer for this helpful suggestion. We have clarified the selection

of model parameters in the revised manuscript. In Eq. 3, K and V_0 set the volume-elasticity and preferred-volume scales and were kept fixed, whereas Γ, λ, and κ represent the independently varied mechanical parameters associated with apical perimeter elasticity, apical junctional line tension, and basolateral surface tension, respectively. In the original manuscript, we mainly analyzed the dependence on Γ and λ while keeping κ fixed. Following the reviewer's suggestion, we have now extended this analysis to examine the influence of κ on epithelial stratification. We mapped the stratification ratio, ξ, over the κ–λ parameter space and found that increasing λ and decreasing κ promote the transition from a monolayered to a multilayered structure. These results are now shown in **Fig. 1m** and described in the **Results**.

[Results, Page 6]
Because K and V_0 set the volume elasticity and preferred-volume scales and were kept fixed in this study, we independently varied κ, Γ, and λ in Equation 2.

[Results, Page 7]
We also quantified the dependence of the layer structure on the apical junctional parameter, λ, and basolateral surface tension, κ (Fig.1m). As λ increase and κ decrease, the epithelial structure transitioned from a monolayer to a multilayer structure. Quantitatively, ξ also jumped from 0 to 1 with an increase in λ and a decrease in κ.

[Fig. 1m & legend]

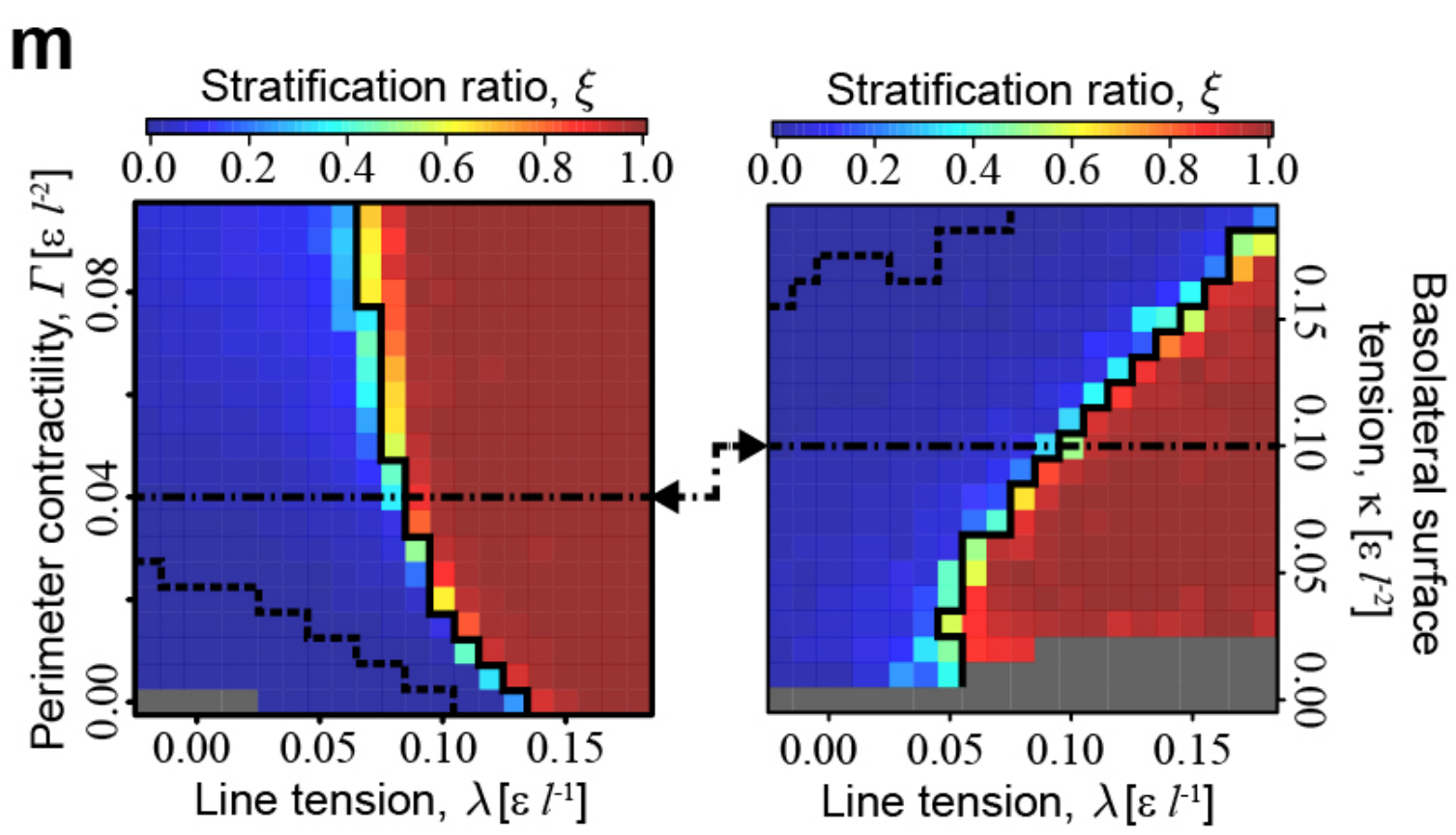

m Stratification ratio, ξ, with respect to Γ and λ (left), with respect to κ and λ (right), respectively. In (m), solid and dotted lines indicate boundaries among the

multilayer ($\xi > 0.5$), mixed layer ($0.5 \geq \xi > 0.0$), and complete monolayer ($\xi = 0.0$). The data in (**l**), (**m**), and (**o**) indicate the average values calculated from five independent initial conditions. Gray regions indicate collapsed states.

Reviewer Comment 1-9:
AFM data need to be described in more detail together with the connections to the parameters of the model – see also below. This will support the mechanical instability statement.

Authors' response 1-9:
We thank the reviewer for this helpful suggestion. We have revised the **Methods** to describe the AFM measurement and analysis in more detail and to clarify how the AFM data should be interpreted in relation to the mechanical regulation of stratification. In our analysis, AFM was used to obtain spatial maps of the apparent elastic modulus, after which junctional and non-junctional cell-body regions were segmented and quantified separately. We do not interpret the AFM-derived modulus as a direct measurement of a specific model parameter. Rather, because the apparent modulus obtained by AFM indentation reflects not only passive material elasticity but also cortical and junctional tension, the modulus measured at junctional regions was used as a relative readout of local junctional tension (Ito et al., Nat Commun 2017).

[Methods, Page 29]
AFM measurements were used to obtain spatial maps of the apparent elastic modulus across the epithelial surface, followed by separate quantification of junctional and non-junctional cell-body regions.

[Methods, Page 29]
Because this apparent modulus reflects not only passive material elasticity but also cortical and junctional tension [83], the values measured at junctional regions were interpreted as a relative readout of local junctional tension rather than as a direct measurement of a model parameter.

Reviewer Comment 1-10:

- Some typos need to be fixed.

Authors' response 1-10:

We thank the reviewer for pointing this out. We have corrected typographical and grammatical errors throughout the manuscript.

Reviewer Comment 1-11:

Figures :

Figure 1: could author specify the number of biological repeats and number of monolayer per biological repeats ? The terms cluster is fine but the term disperse can be misleading. Fig1-D model points : can the authors discuss the reliability of the plot ?

Authors' response 1-11:

We thank the reviewer for these helpful suggestions. We revised the Figure 1 legend to specify the number of biological replicates and monolayers analyzed, replaced the term "disperse" with "isolated," and added the fitting procedure and coefficient of determination for the JMAK model to clarify the reliability of this phenomenological fit in the **Results**.

[Results, Page 8]

The time evolution of ξ in MDCK cell culture was well fitted by the JMAK model (Fig. 1c, Supplementary Fig. 2a; detailed in Materials and Methods), with a high coefficient of determination (R^2 = 0.90).

Reviewer Comment 1-12:

Figure 2 : the stratification ratio is the relevant readout. However the author should discuss the sensitivity of parameters to give these results. Intuitive explanation for the mechanism at play could be needed for panel J.

Authors' response 1-12:

We expanded the **Results** to discuss the parameter sensitivity of the stratification ratio in **Fig. 2j** of the previous manuscript, now shown in **Fig. 1m** of the revised manuscript. We found that the stratification ratio was more sensitive to λ and κ than to Γ within the examined parameter range. We also added an intuitive explanation that λ and κ regulate the balance of apical, basal, and basolateral

mechanical tensions that promote multilayer formation, whereas Γ mainly shifts the onset position of stratification. These explanations have been added to the **Results**.

[Results, Page 7]

The stratification ratio depended more strongly on λ and κ than on Γ within the examined parameter range, indicating that λ and κ primarily control the mechanical balance leading to stratification. In contrast, Γ mainly shifted the onset of stratification without substantially altering the overall structural behavior.

Reviewer Comment 1-13:

Figure 3 : it would be clearer to have analysis and back-to-back with experimental data. A suggestion is to restructure Figures to streamline the paper.

Authors' response 1-13:

We thank the reviewer for this helpful suggestion. We have reorganized the figures and corresponding **Results** to place the numerical simulations directly alongside the matching experimental data, allowing clearer back-to-back comparisons. Specifically, Figure 1 now presents the monolayer-to-multilayer transition observed in MDCK cells together with the corresponding 3D vertex model simulations. Figure 2 now presents the nucleation-and-growth dynamics and related analyses in MDCK cells together with the corresponding simulations. We believe that this revised structure streamlines the presentation and clarifies the relationship between the experimental observations and the theoretical analysis.

Reviewer Comment 1-14:

Figure 4 : why presenting this as a new model? Also, could authors discuss more the AFM experiments relevance with parameters of the model? The connection is not straightforward. In addition, could they do laser ablation to back up the comparison between experiments and theory?

Authors' response 1-14:

We apologize for the misleading impression in the original manuscript. We revised the text to clarify that we are not proposing an entirely new model, but

applying an established 3D vertex-model framework to epithelial stratification to derive simple mechanistic insights. We also clarified that AFM-derived apparent stiffness at junctional regions is interpreted as a relative readout of junctional tension, rather than as a direct measurement of a specific model parameter (see also response 1-9). Although laser ablation would be valuable for directly assessing junctional tension, reliable ablation and recoil quantification were not feasible in our multilayered MDCK samples because of tissue thickness and limited optical access with the commercial laser systems available to us. We have added this limitation as an important direction for future work.

Reviewer Comment 1-15:

Figure 5 : the same comment as above, it would be important that authors are critical about the robustness of the numerical simulation.

Authors' response 1-15:

We thank the reviewer for this helpful suggestion. To further assess the robustness of the numerical simulations, we added a parameter-sensitivity analysis of scutoid formation over a range of Γ and λ. This analysis shows that scutoids are not observed only at a single fine-tuned parameter set, but appear broadly in the parameter regions where stratification occurs. We also report the average over five independent initial conditions and indicate collapsed states separately. These results are now shown in **Fig. 4j** and described in the **Results**.

[Results, Page 13]

Moreover, scutoids became more frequent as stratification progressed and were observed in both mixed layer and complete multilayer structures (Fig. 4j). However, within the complete multilayer regime near the monolayer boundary at low Γ, scutoid numbers remained relatively low. This may reflect a regime in which domain growth contributes relatively more than new domain formation (Fig. 2i; Supplementary Figs. 2d, e and 3g).

[Figure 4j & legend]

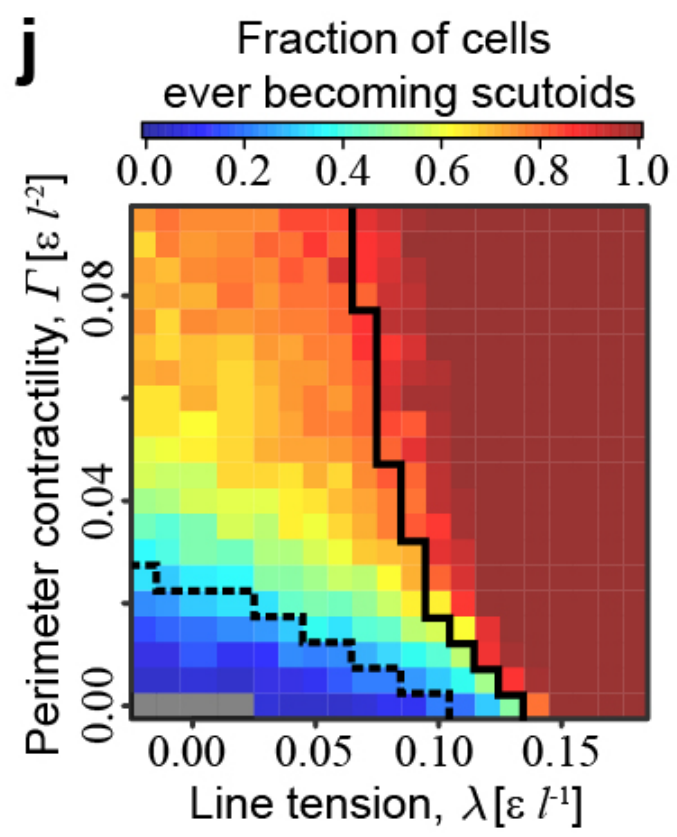

j Fraction of cells ever becoming scutoids with respect to Γ and λ. In (j), solid and dotted lines indicate boundaries among the multilayer ($\xi > 0.5$), mixed layer ($0.5 \geq \xi > 0.0$), and complete monolayer ($\xi = 0.0$). The data in (j) indicate the average values calculated from five independent initial conditions. Gray regions indicate collapsed states.

Reviewer Comment 1-16:

Figure 6 : if possible, it would be nice to have more timepoints like in Fig. 1 to show the nucleation and growth mechanism in these systems – as convincingly as for MDCK cells.

Authors' response 1-16:

Following the reviewer's comment, we added additional time points for the malignant transformation organoid system to better visualize the temporal progression of nucleation and growth, analogous to the MDCK time course. We also quantified the stratification ratio and the temporal changes in isolated and clustered delaminated cells to show how local delamination events develop into clustered stratified regions. These new data are now included in **Fig. 5j–l**.

[Figure 5j-l & legend]

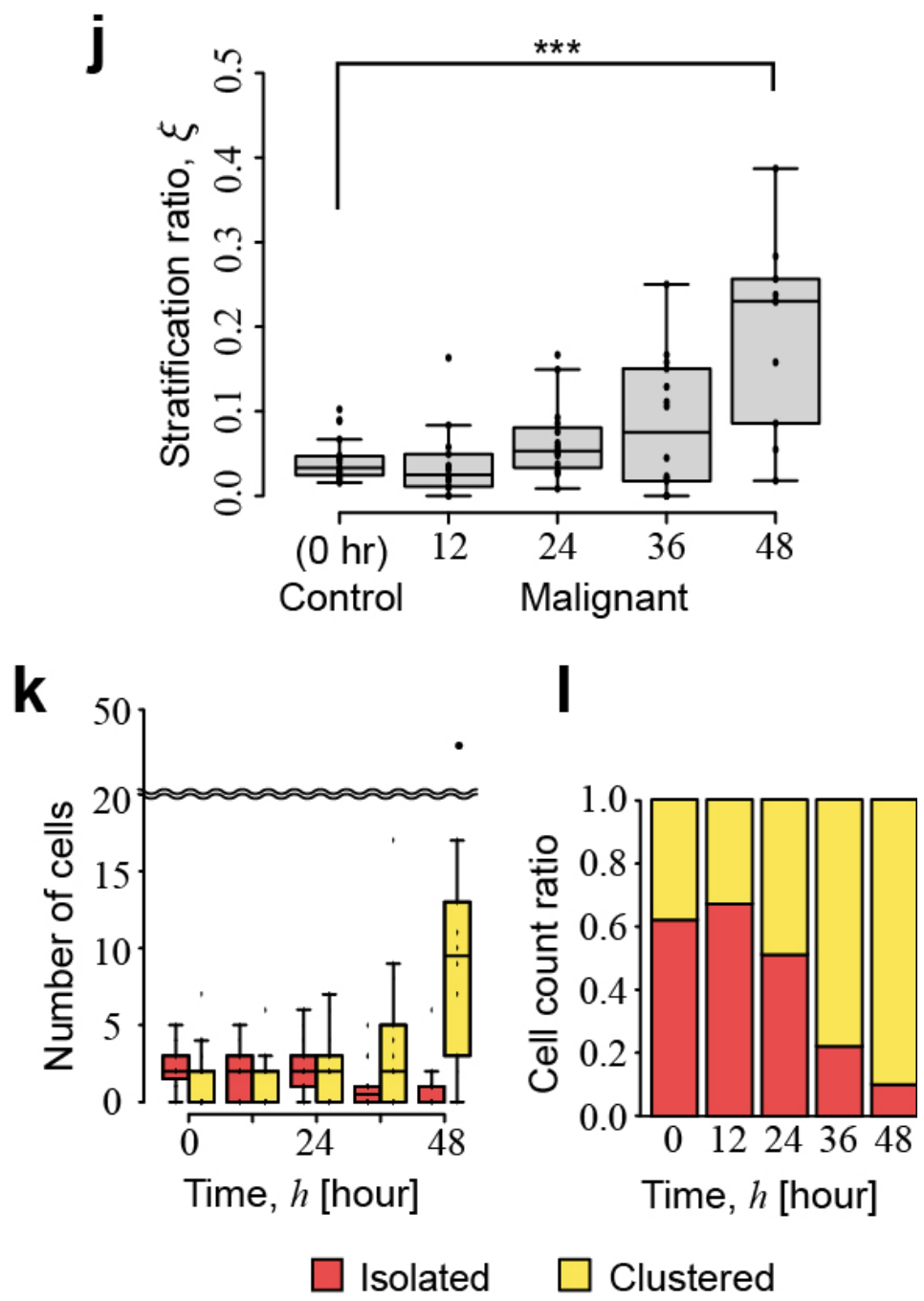

j Stratification ratio, ξ, under control and malignant induction. **k** Temporal changes in the number of isolated versus clustered delaminated cells. **l** Temporal changes in the ratio of isolated and clustered delaminated cells. In (**c**), and (**i**), isolated and clustered delaminated cells are colored red and yellow, respectively. Plots in (**j**), (**k**), and (**l**) include data from ten or more images from three or more biologically independent samples. Box plots in (**d**), (**e**), (**f**), (**j**), (**k**), and (**l**): center: median; bounds, 25th and 75th percentiles; whiskers, min and max; ***$P < 0.001$ (two-sided Welch's t-test).

RESPONSE TO REVIEWER #2

Reviewer Comment:

The manuscript by Fukamachi et al. investigates epithelial delamination and stratification by combining experimental observations, numerical simulations, and theoretical arguments. The authors follow the temporal evolution of an MDCK monolayer transitioning into a multilayered tissue and compare these dynamics with simulations based on a 3D vertex model. They propose that stratification can be understood as a nucleation-and-growth process driven by mechanical instabilities within the tissue. Overall, the study addresses an interesting problem and presents results that could be impactful for the field. However, much of the analysis remains largely descriptive. In my view, the manuscript would benefit from a more quantitative and systematic investigation to substantiate the proposed mechanisms and to move beyond phenomenological observations. As it stands, the mechanistic insights and resulting conclusions appear somewhat superficial. Therefore, I advise a major revision to address key concerns listed below.

Reviewer Comment 2-1:

A major concern relates to the role and usefulness of the mean-field model. The authors show that varying the mean-field parameters can give rise to stable configurations with delaminated cells. However, contrary to previous reports, they also state that the aim of the study is to identify how individual parameters contribute to the stratification process at the tissue level. In this context, it is unclear why the insights obtained from the mean-field model are not translated into measurable or controllable quantities within the 3D vertex model. These quantities could then be directly compared with experimental observations. Establishing such a link would substantially strengthen the mechanistic interpretation of the results. More specifically:

Reviewer Comment 2-1-1:

a) The authors compare predictions from the mean-field model with experimentally measured Young's moduli and report a discrepancy between theory and experiment. It is therefore important to clarify how the 3D vertex model behaves in this regard. Based on Fig. 2k, the line tension appears to increase during stratification. Does this indicate that the numerical simulations are likewise inconsistent with the experimental measurements? Furthermore, can Fig. 2k be interpreted in a manner that allows a direct connection to Fig. 4h? If such a connection exists, it remains unclear why apical junctional tension does not

emerge as a key driver of stratification, as predicted by the mean-field model. Are there competing mechanisms or compensatory effects in the simulations that could account for the experimental observations?

Authors' response 2-1-1:
We thank the reviewer for raising this important point. We have revised the manuscript to clarify how the mean-field model, 3D vertex-model simulations, and AFM measurements should be interpreted together. The mean-field model identifies several possible routes to mechanical destabilization of the embedded state, including increased apical line tension, increased planar cell density, and reduced neighbor number, but it does not determine which route is dominant in MDCK cell culture. Experimentally, the apparent Young's modulus measured at junctional regions decreased with increasing stratification, suggesting that increased junctional tension is not the primary driver in this system. In contrast, planar cell density increased during stratification, and suppressing this increase by aphidicolin treatment reduced stratification (**Fig. 3i–k**). We also clarified that, in the 3D vertex model, planar cell density was not imposed as an independent control parameter, but emerged from tissue geometry depending on Γ and λ (**Supplementary Fig. 3d, e**); the stratification ratio generally increased with planar cell density in the simulations (**Fig. 3l**). Together, these results support the interpretation that planar cell density, rather than increased apical junctional tension, is the dominant driver of stratification in MDCK cell culture. This discussion has been added to the **Results**.

[Results, Page 11]
To further test this association, we inhibited cell proliferation using aphidicolin at 6 and 24 hours after seeding (Fig. 3i), corresponding to the periods when nucleation and growth predominantly occur, respectively (Fig. 2a, b). Aphidicolin treatment significantly suppressed the increase in ξ compared with the control (Fig. 3j) and consistently suppressed the increase in ξ at the corresponding time points (Fig. 3k). These results suggest that increasing planar cell density contributes to the progression of epithelial stratification in MDCK cell culture.

We next examined how planar cell density contributes to epithelial stratification in the simulations. Planar cell density varied depending on Γ and λ and increased with λ across the examined parameter range (Supplementary Fig.

3d, e). The stratification ratio generally increased with increasing density, although the relationship between density and layer structure varied across parameter regimes (Fig. 3l). Because domain growth occurs adjacent to pre-existing multilayer domains, we next focused on differences around these domains. We classified the monolayer region into potential nucleation and growth regions (Supplementary Fig. 3f). The potential nucleation region consisted of cells located away from existing multilayer domains, whereas the potential growth region consisted of cells adjacent to multilayer domains. We then calculated the frequency of delamination events in each region (Supplementary Fig. 3g).

Across most values of Γ and λ, delamination events occurred more frequently in the potential growth region than in the potential nucleation region. Moreover, the apparent planar cell density, defined for each cell as the inverse of the mean of its apical and basal areas (see Materials and Methods), was consistently higher on average in the potential growth region than in the potential nucleation region (Supplementary Fig. 3h). These results suggest that pre-existing multilayer domains locally increase cell crowding, thereby facilitating nearby delamination events and promoting cooperative domain growth during epithelial stratification.

[Figure 3i-k & legend]

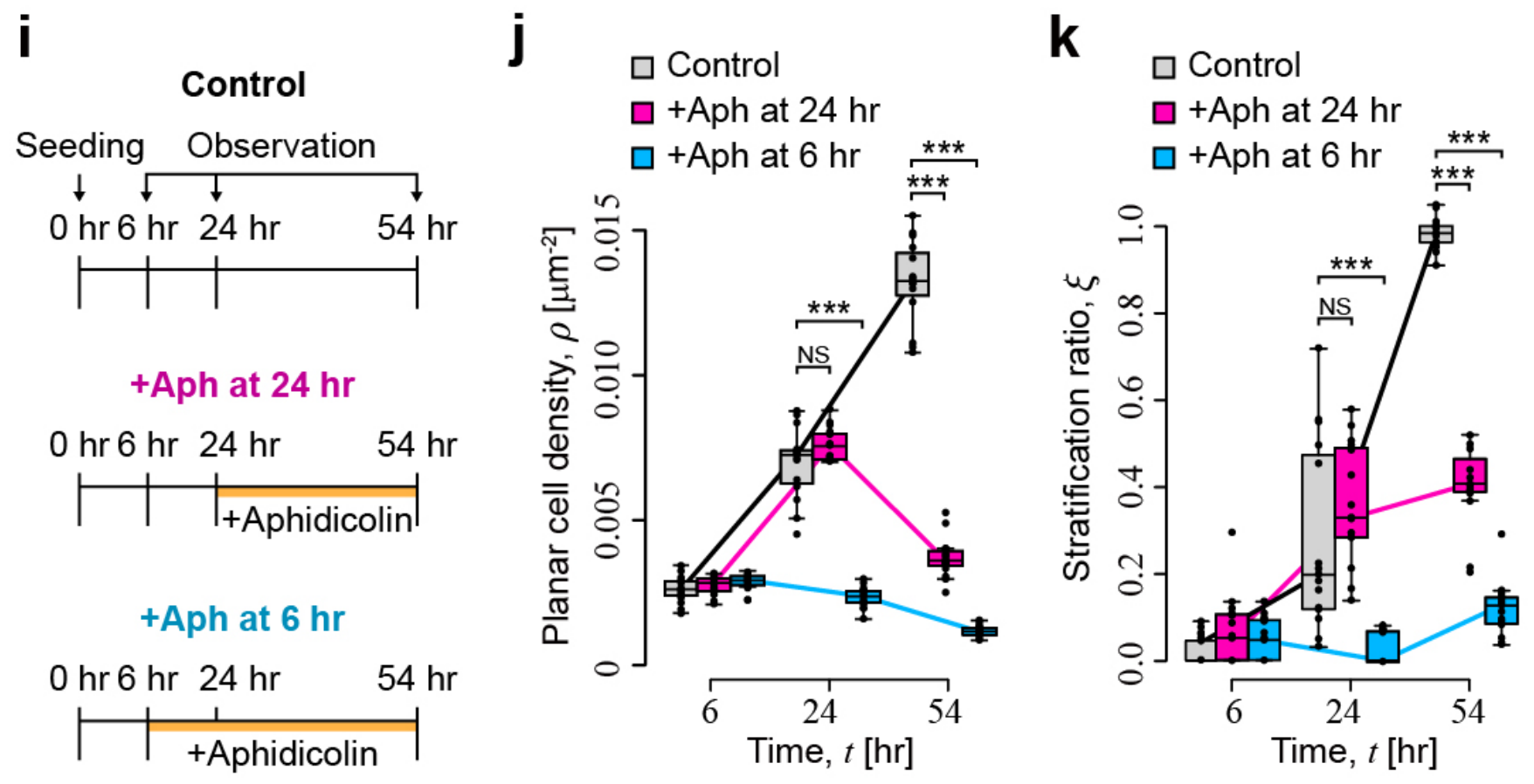

i–**k**, Aphidicolin perturbation assays in MDCK cells. Aphidicolin was added at 6 or 24 h after seeding to suppress the time-dependent increase in planar cell density. **i**, Treatment scheme. **j**, Planar cell density, ρ, as a function of time. **k**, Stratification ratio, ξ, as a function of time. Plots in (**e**), (**h**), (**i**), (**j**), and (**k**)

include data from 15 images from three biologically independent samples. Box plots in (**h**), (**j**), and (**k**): center, median; bounds, 25th and 75th percentiles; whiskers, min and max; ***P < 0.001 (two-sided Welch's t-test).

[Supplementary Fig. 3d, e]

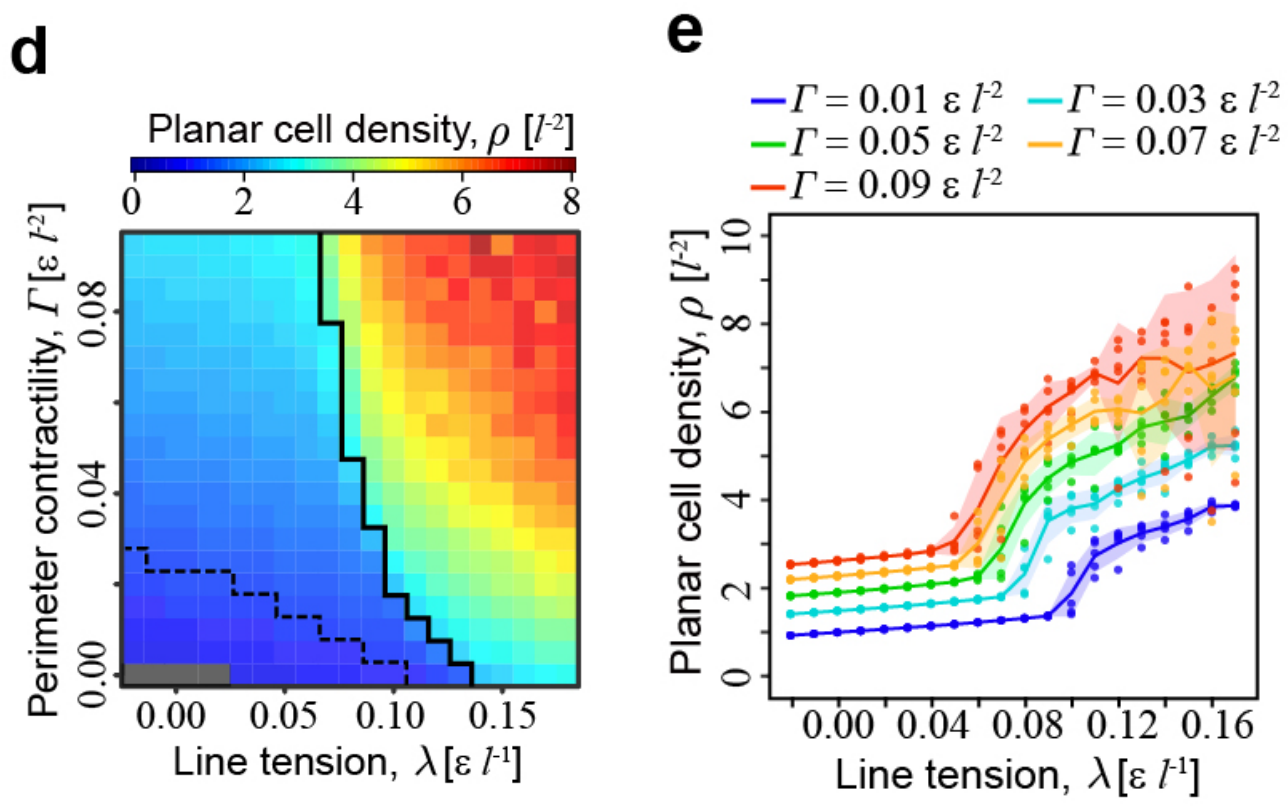

d Planar cell density, ρ, with respect to Γ and λ. **e** Planar cell density, ρ, as a function of λ. In (**d**), (**g**), and (**h**), solid and dotted lines indicate boundaries among the multilayer ($\xi > 0.5$), mixed layer ($0.5 \geq \xi > 0.0$), and complete monolayer ($\xi = 0.0$). In (**d**), (**g**), and (**h**), the gray-colored regions indicate the collapsed state. The data in (**d**), (**g**), and (**h**) indicate the average values calculated using five initial conditions.

[Fig. 3l & legend]

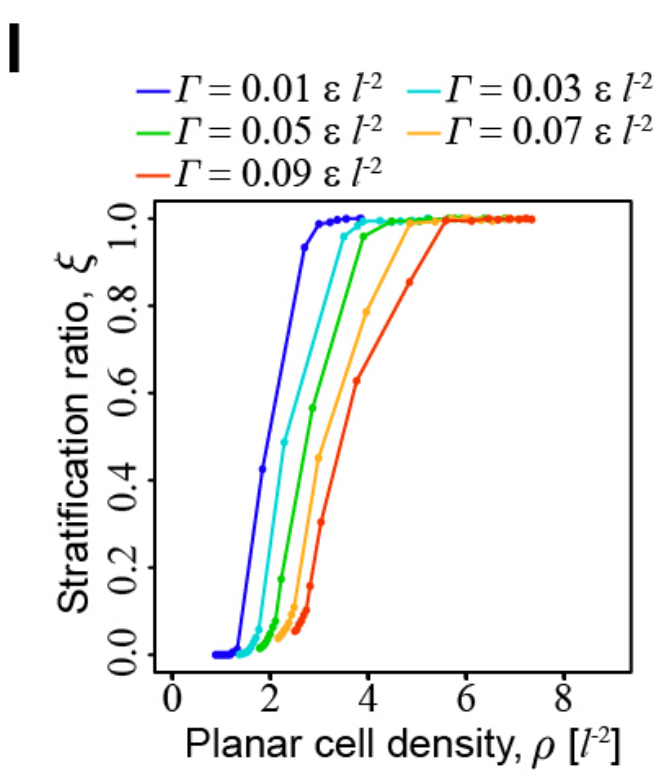

l Stratification ratio, ξ, as a function of planar cell density ρ, for different values of λ. The data in (e),and (l) indicate the average values calculated from five independent initial conditions.

Reviewer Comment 2-1-2:

b) Along similar lines, it would be useful to assess whether the 3D vertex model can reproduce the probability distribution shown in Fig. 4f. Consistency between the simulations and experiments would help validate the relevance of the model.

Authors' response 2-1-2:

To address this point, we calculated the probability distribution of the cell taper parameter, d, in the 3D vertex-model simulations using the same definition as in the experimental analysis and compared it with the MDCK data. The simulations qualitatively recapitulated the trimodal distribution observed in experiments, with peaks corresponding to embedded cells and apically or basally delaminated cells. This result supports the interpretation that stratification proceeds through a mechanical instability in which cells preferentially adopt either embedded or delaminated states rather than intermediate configurations. These new results are now presented in **Fig. 3e** and described in the **Results**.

[Results, Page 10]

To examine whether this predicted mechanical instability is reflected in epithelial stratification, we calculated the probability distribution of cell taper, d in MDCK cell culture and 3D vertex-model simulations (Fig. 3e). Both experiments and simulations showed a trimodal distribution around d = -1, 0, and 1, with two distinct gaps between d = -1 and 0 and between d = 0 and 1. This pattern indicates that cells predominantly adopt either an embedded state or apically or basally delaminated states, rather than remaining in intermediate configurations. These observations are consistent with the idea that loss of stability of the embedded state drives cells toward delamination through an inherent mechanical instability.

[Figure 3e & legend]

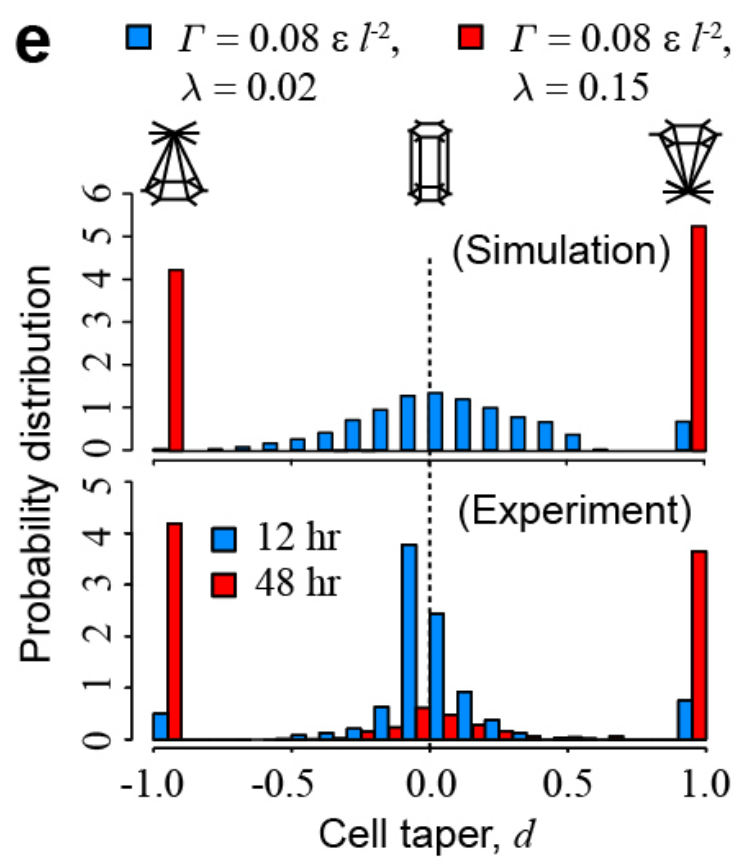

e Probability distribution of the cell taper, d, calculated from 3D vertex-model simulations and experimental MDCK data. Blue and red indicate low- and high-stratification conditions, respectively, corresponding to $\lambda = 0.02\,\varepsilon l^{-1}$ and $\lambda = 0.15\,\varepsilon l^{-1}$ in simulations, and 12 and 48 h in MDCK cultures. Simulations were performed with $\Gamma = 0.08\,\varepsilon l^{-2}$. Plots in (**e**), (**h**), (**i**), (**j**), and (**k**) include data from 15 images from three biologically independent samples. The data in (e), and (l) indicate the average values calculated from five independent initial conditions.

Reviewer Comment 2-1-3:

c) For the analysis as a function of planar density, it would be informative to include vertex-model results analogous to Fig. 4i–j. For which choices of the Hamiltonian parameters does the vertex model recover the observed dependence of the stratification ratio on density? Related to this point, the definition of density itself is currently unclear and inconsistent throughout the manuscript. In one section, density is defined as the inverse of the average apical and basal surface area; in the Methods, it is defined as the number of cells divided by the total image area; and in the mean-field model, density is introduced in Eq. 13 without an explicit definition. I strongly encourage the authors to clarify and unify the definition of density and to provide a clear derivation of Eqs. 13–18, as their origin and assumptions are currently difficult to follow.

Authors' response 2-1-3:

We thank the reviewer for this important suggestion. We apologize that the definition and usage of planar cell density were not sufficiently clear in the original manuscript, and that one description of density was inaccurate. In the

revised manuscript, we have unified the definition of planar cell density, ρ, as the number of cells per unit projected area, and clarified how this quantity is calculated in experiments, simulations, and the mean-field analysis. Following the reviewer's suggestion, we added vertex-model results analogous to the experimental density analysis by quantifying the stratification ratio, ξ, as a function of ρ in the simulations (**Fig. 3l**). This analysis shows that ξ generally increases with ρ, particularly in parameter regimes where the monolayer is mechanically close to multilayer formation, qualitatively reproducing the density dependence observed in MDCK cell culture. We have revised the **Results**, **Methods**, and figure legend accordingly, and expanded the derivation of Eqs. 13–18 to clarify the origin and assumptions of the mean-field analysis.

[Results, Page 11]
We next examined how planar cell density contributes to epithelial stratification in the simulations. Planar cell density varied depending on Γ and λ and increased with λ across the examined parameter range (Supplementary Fig. 3d, e). The stratification ratio generally increased with increasing density, although the relationship between density and layer structure varied across parameter regimes (Fig. 3l).

[Methods, Page 20]
Planar cell density, ρ, was defined as the number of cells per unit projected area in both experiments and simulations. In MDCK experiments, cells were counted using mCherry-CAAX fluorescence. To account for cells intersecting the image boundary, the number of cells was calculated as $N_{\mathrm{c}} + N_{\mathrm{e}}/2$, where N_{c} is the number of cells fully contained within an image and N_{e} is the number of cells intersecting the image boundary. ρ was then calculated by dividing this cell number by the total image area. To analyze the relationship between ξ and ρ, both quantities were calculated for each individual image. In simulations, where periodic boundary conditions were imposed, ρ was calculated as $N_{\mathrm{t}}/(L_x L_y)$, where N_{t} is the total number of cells and L_x and L_y are the system sizes in the x- and y-directions, respectively.

[Methods, Page 27]
Specifically, we considered an epithelial monolayer of thickness (T), composed of cells with a fixed volume (V_0). Under this assumption, the projected area per

cell is V_0/T. Therefore, the planar cell density is expressed as $\rho = T/V_0$, which is consistent with the definition of cell number per unit projected area used in the experiments and simulations.

[Methods, Page 27]

Moreover, the packing geometry imposes geometric constraints on the physical parameters. The volume of the frustum is calculated as

$$V_0 = \frac{T(A_\mathrm{a} + \sqrt{A_\mathrm{a}A_\mathrm{b}} + A_\mathrm{b})}{3}. \tag{15}$$

By solving Equations 14 and 15 simultaneously, with $\rho = T/V_0$, we obtained A_a, and A_b as functions of ρ and d as described by

$$A_\mathrm{a}(\rho, d) = \frac{3(d+1)^2}{\rho(d^2+3)}, \tag{16}$$

$$A_\mathrm{b}(\rho, d) = \frac{3(d-1)^2}{\rho(d^2+3)}. \tag{17}$$

Additionally, we introduced several geometric parameters, including apical perimeter of the center cell P_c (≥ 0); total area of the n boundary faces between the center cell and its first-neighbor cells A_c (≥ 0); and total area of the n boundary faces between the first-neighbor cells A_p.

[Fig. 3l & legend]

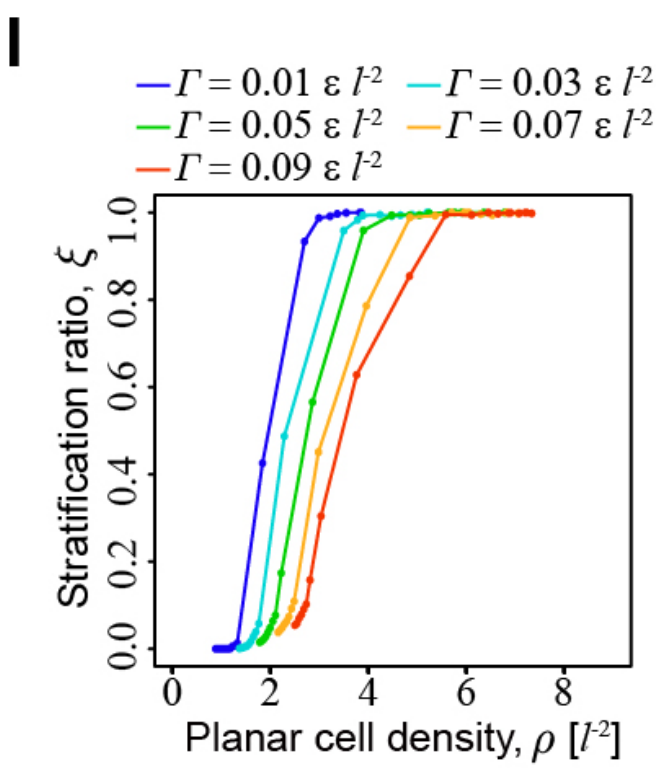

l Stratification ratio, ξ, as a function of planar cell density ρ in the 3D vertex model, for different values of Γ. The data in (e), and (l) indicate the average values calculated from five independent initial conditions.

Reviewer Comment 2-2:
A central conclusion of the manuscript is that epithelial stratification is triggered by mechanical instabilities induced by apical tension, planar density, or the number of adjacent cells, leading to a nucleation-and-growth–like process. However, the evidence supporting this claim remains largely observational. If the transition to delaminated states is indeed driven by mechanical instabilities, this should be quantitatively demonstrated within the 3D vertex model, for example by analyzing the Hessian of the mechanical energy as a function of time. Do the eigenvalues or normal modes of the Hessian exhibit qualitative or quantitative changes preceding or accompanying stratification? Are such changes correlated with variations in planar density? Performing these analyses would substantially strengthen the mechanistic basis of the proposed interpretation. A similar stability analysis could also be carried out within the mean-field framework. Overall, such assessments would be more informative than the schematic illustration presented in Fig. 4e.

Authors' response 2-2:
We thank the reviewer for this important suggestion. Following the reviewer's comment, we quantitatively assessed local mechanical stability in the 3D vertex-model simulations by analyzing the Hessian matrix of the mechanical energy around individual delaminating cells. We found that cells approaching delamination exhibited an increase in the number of negative eigenvalues immediately before detachment, indicating that the local energy landscape becomes mechanically unstable prior to cell delamination. This analysis provides quantitative support for our interpretation that delamination, and consequently epithelial stratification, is associated with mechanical instability. These new results are now presented in **Fig. 3f, g** and described in the **Results**.

[Results, Page 10]
Furthermore, to quantitatively assess local mechanical stability in the simulations, we analyzed the Hessian matrix of the effective energy around individual delaminating cells in the 3D vertex model (see Materials and Methods). Cells approaching delamination showed an increase in the number of negative eigenvalues immediately before delamination, indicating a loss of local mechanical stability in the energy landscape (Fig. 3f, g). These results suggest that cell delamination, and consequently epithelial stratification, is preceded by mechanically unstable cell states.

[Figure 3f, g & legend]

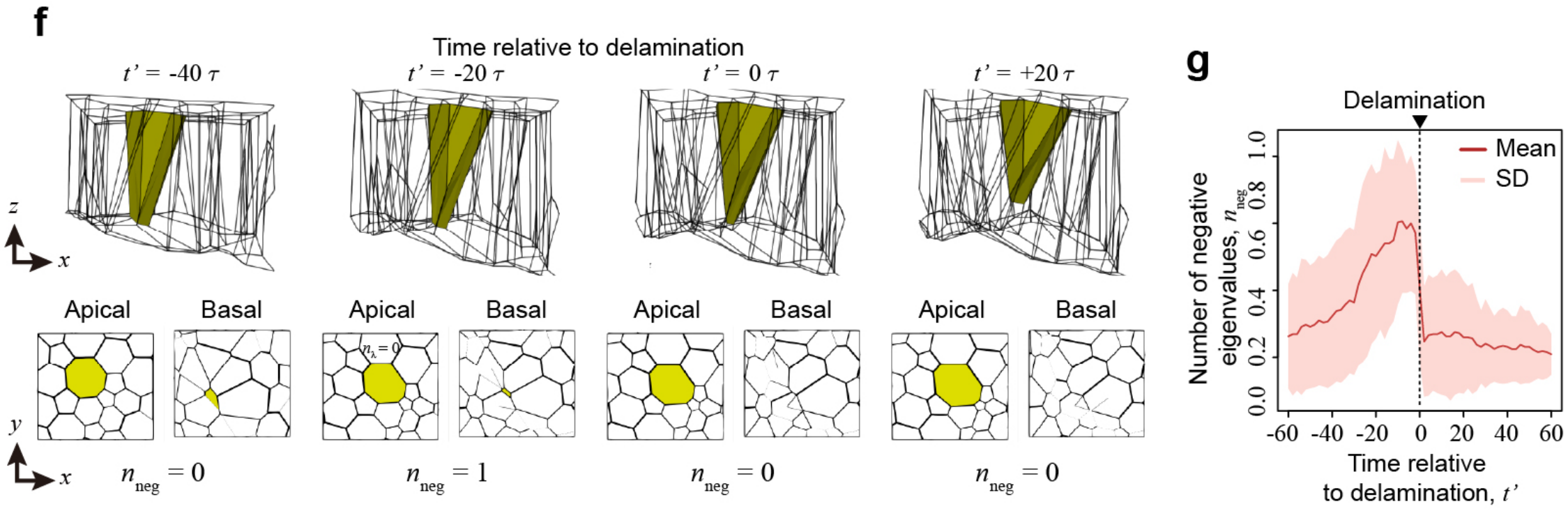

f, **g,** Temporal evolution of mechanical instability during cell delamination in the 3D vertex model. In (**f**), representative snapshots show cell delamination. Top and bottom images show 3D and cross-sectional views, respectively. The number of negative eigenvalues of the Hessian matrix for the delaminating cell, n_{neg}, is indicated below each image. In (**g**), n_{neg} is plotted as a function of time relative to delamination, t'. Data represent mean ± s.d.

Reviewer Comment 2-3:

In the 3D vertex model, the distinction between analyses of equilibrium configurations obtained after relaxation and analyses of the system's temporal dynamics is currently unclear. For instance, it is not evident how Fig. 2l was generated. The authors state that the parameters Γ and λ were varied over time, but the specific protocol used to implement these temporal changes is not described. Similarly, the origin of the dynamics shown in Fig. 3 is ambiguous. Do these results simply represent the time evolution of the system during energy minimization? Or do they include additional dynamical ingredients, such as self-propelled or active forces? If the former, it is important to clarify whether the reported dynamics correspond to transient states on the way to equilibrium or to a well-defined nonequilibrium process. I strongly encourage the authors to provide more detail on how temporal dynamics are defined and implemented in the vertex-model simulations.

Authors' response 2-3:

We thank the reviewer for pointing out this important ambiguity, and we apologize for the insufficient and partially incorrect description in the original manuscript. We have corrected the statement that "Γ and λ were varied over time" in the Results. The corresponding panel in the previous manuscript did not involve a temporal protocol with time-varying parameters; rather, it showed equilibrium configurations obtained after energy minimization from different

initial conditions, which were used to assess dependence on initialization. No additional dynamical ingredients, such as self-propulsion or active forces, were included. For the panels showing temporal progression in the simulations, the trajectories correspond to numerical relaxation during energy minimization, not to explicitly defined biological time or a prescribed physical nonequilibrium process. We have clarified this distinction between equilibrium configurations and relaxation trajectories in the **Results** and added the full simulation protocol to the **Methods**.

[Results, Page 7]

Using this model, we numerically calculated the energy relaxation process starting from an initial condition of a complete monolayer (Fig. 1d; detailed in Materials and Methods) and obtained an equilibrium structure at the local energy minimum.

[Methods, Page 25]

Time in these simulations therefore corresponds to numerical relaxation time rather than direct biological time. Simulations were continued until the average vertex displacement became small, indicating convergence to a mechanically stable state. No additional active dynamics, such as self-propulsion or externally imposed active forces, were included.

Reviewer Comment 2-4:

Another key conclusion of the manuscript is that epithelial stratification proceeds via a nucleation-and-growth process. Given that this represents one of the central results of the study, a more in-depth and quantitative analysis is needed. In particular, it would be important to characterize the critical nucleus size (or critical radius) associated with the onset of stratification and to examine how this quantity depends on the relevant control parameters. Such an analysis could clarify which factors dominate the growth phase and how these are related to the mechanical instabilities identified in the mean-field model. More specifically, if apical tension and planar density primarily trigger isolated delamination events, what mechanisms promote the growth of delaminated clusters?

Authors' response 2-4:

We thank the reviewer for this important suggestion. To further quantify the nucleation-and-growth process, we analyzed the number and size of stratified

domains over the Γ–λ parameter space (**Fig. 2i, j; Supplementary Fig. 2d, e**). We also retained the nucleation and growth ratio analyses in the Supplementary Information (**Supplementary Fig. 2f, g**), which show that the growth ratio more clearly distinguishes monolayered and multilayered states than the nucleation ratio. These analyses indicate that stratification proceeds mainly through growth of existing domains, while the frequency of new domain formation is modulated by apical junctional parameters. To further clarify what promotes domain growth, we compared regions adjacent to pre-existing multilayer domains with regions away from them (**Supplementary Fig. 3f**). Delamination events were more frequent near pre-existing domains (**Supplementary Fig. 3g**), where apparent planar cell density was also higher (**Supplementary Fig. 3h**). These results suggest that local crowding around stratified domains facilitates neighboring delamination and promotes cooperative domain growth. These analyses and the corresponding discussion have been added to the **Results**.

[Results, Page 9]

To further characterize the nucleation-growth process in the simulations, we analyzed the number and size of multilayer domains during epithelial stratification (Fig. 2i, j; Supplementary Fig. 2d, e). Domains were quantified by identifying contiguous multilayer regions. Across the examined Γ–λ parameter range, stratification proceeded mainly through the growth of existing domains, whereas the number of newly formed domains varied depending on the apical junctional parameters. Consistently, analysis of the nucleation ratio, ψ_{n}, and growth ratio, ψ_{g}, showed that ψ_{g} more clearly distinguished monolayered and multilayered states than ψ_{n} (Supplementary Fig. 2f, g). These results indicate that apical junctional parameters modulate the frequency of nucleation events, whereas subsequent domain growth is the major contributor to multilayer expansion.

[Results, Page 11]

We next examined how planar cell density contributes to epithelial stratification in the simulations. Planar cell density varied depending on Γ and λ and increased with λ across the examined parameter range (Supplementary Fig. 3d, e). The stratification ratio generally increased with increasing density, although the relationship between density and layer structure varied across parameter regimes (Fig. 3l). Because domain growth occurs adjacent to pre-existing multilayer

domains, we next focused on differences around these domains. We classified the monolayer region into potential nucleation and growth regions (Supplementary Fig. 3f). The potential nucleation region consisted of cells located away from existing multilayer domains, whereas the potential growth region consisted of cells adjacent to multilayer domains. We then calculated the frequency of delamination events in each region (Supplementary Fig. 3g).

Across most values of Γ and λ, delamination events occurred more frequently in the potential growth region than in the potential nucleation region. Moreover, the apparent planar cell density, defined for each cell as the inverse of the mean of its apical and basal areas (see Materials and Methods), was consistently higher on average in the potential growth region than in the potential nucleation region (Supplementary Fig. 3h). These results suggest that pre-existing multilayer domains locally increase cell crowding, thereby facilitating nearby delamination events and promoting cooperative domain growth during epithelial stratification.

[Fig. 2i, j & legend]

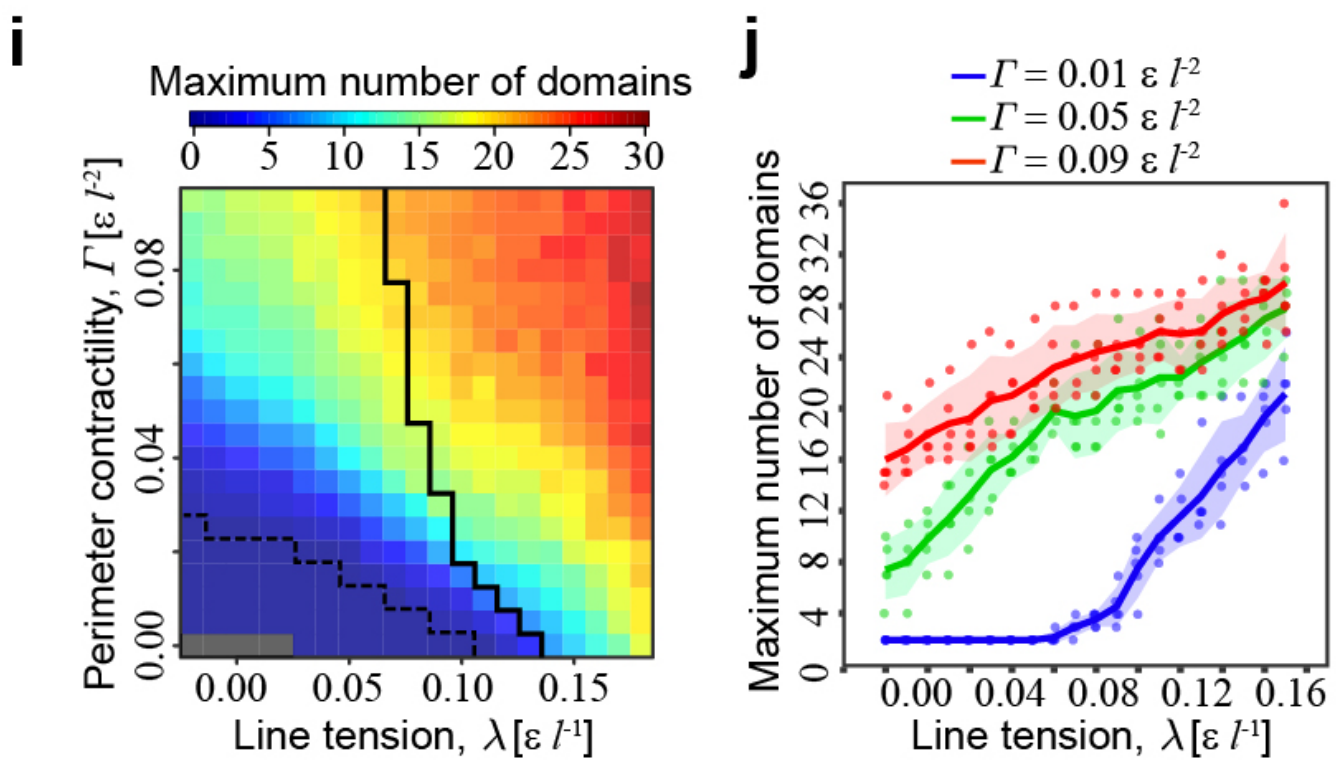

i Maximum number of domains over time with respect to Γ and λ. **j** Maximum number of domains over time as a function of λ. In (**i**), solid and dotted lines indicate boundaries among the multilayer ($\xi > 0.5$), mixed layer ($0.5 \geq \xi > 0.0$), and complete monolayer ($\xi = 0.0$). In (**i**), the gray-colored regions indicate the collapsed state. The data in (**i**) indicate the average values calculated using five initial conditions. Gray regions indicate collapsed states. Band in (**j**) indicates the standard deviation.

[Supplementary Fig. 2d, e & legend]

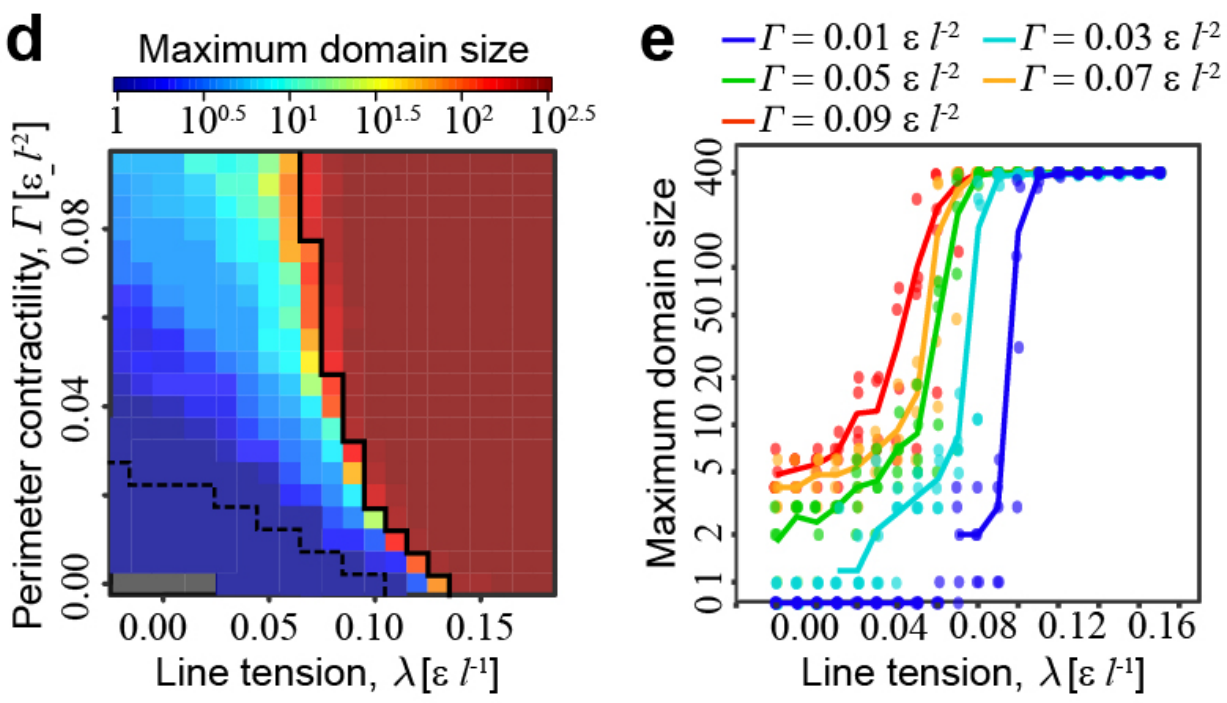

d Maximum domain size over time with respect to Γ and λ. **e** Maximum domain size over time as a function of λ. In (**c**), (**d**), (**e**), and (**f**), solid and dotted lines indicate boundaries among the multilayer ($\xi > 0.5$), mixed layer ($0.5 \geq \xi > 0.0$), and complete monolayer ($\xi = 0.0$). The data in (**c**), (**d**), (**e**), and (**f**) indicate the average values calculated from five independent initial conditions. Gray regions indicate collapsed states.

[Supplementary Fig. 3f-h & legend]

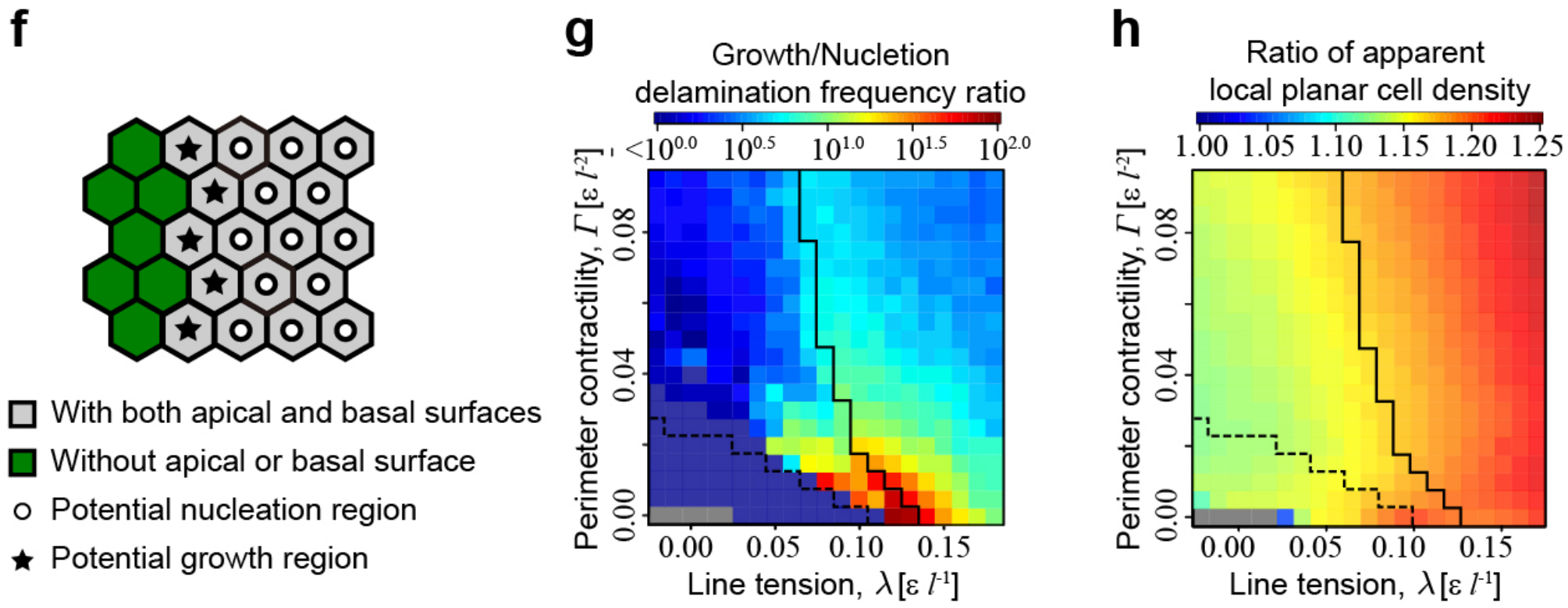

f Schematic illustration of the potential nucleation and growth regions. **g** Ratio of cell delamination frequency in the potential growth region to that in the potential nucleation region. **h** Ratio of apparent local planar cell density in the potential growth region to that in the potential nucleation region. Apparent local planar cell density was defined as the inverse of the mean planar cell area across the apical and basal surfaces (see Materials and Methods). In (**d**), (**g**), and (**h**), solid and dotted lines indicate boundaries among the multilayer ($\xi > 0.5$), mixed layer ($0.5 \geq \xi > 0.0$), and complete monolayer ($\xi = 0.0$). Gray regions indicate collapsed states. The data in (**d**), (**g**), and (**h**) indicate average values calculated

from five initial conditions.

Reviewer Comment 2-5:

In their investigation of the 3D vertex model, the authors vary only the parameters Γ and λ. It is unclear why the parameter k, which controls the basolateral surface energy, was not also explored. I would expect this parameter to contribute to the overall mechanical stability of the tissue and, potentially, to the delamination process. It would be helpful clarifying the rationale for fixing k, or alternatively exploring its effect on stratification.

Authors' response 2-5:

We thank the reviewer for this helpful suggestion. In the revised manuscript, we explored the effect of the basolateral surface-tension parameter, κ, in the 3D vertex model. By mapping the stratification ratio, ξ, over the κ–λ parameter space, we found that decreasing κ promotes stratification and shifts the boundary between monolayered and multilayered states. Importantly, the qualitative behavior was preserved: the monolayer-to-multilayer structural transition occurred over a range of κ values. These new results are now presented in **Fig. 1m** and described in the **Results**.

[Results, Page 6]

Because K and V_0 set the volume elasticity and preferred-volume scales and were kept fixed in this study, we independently varied κ, Γ, and λ in Equation 2.

[Results, Page 7]

We also quantified the dependence of the layer structure on the apical junctional parameter λ and basolateral surface tension κ (Fig.1m). As λ increases and κ decreases, the epithelial structure transitioned from a monolayer to a multilayer structure. Quantitatively, ξ also jumped from 0 to 1 with an increase in λ and a decrease in κ.

[Fig. 1m & legend]

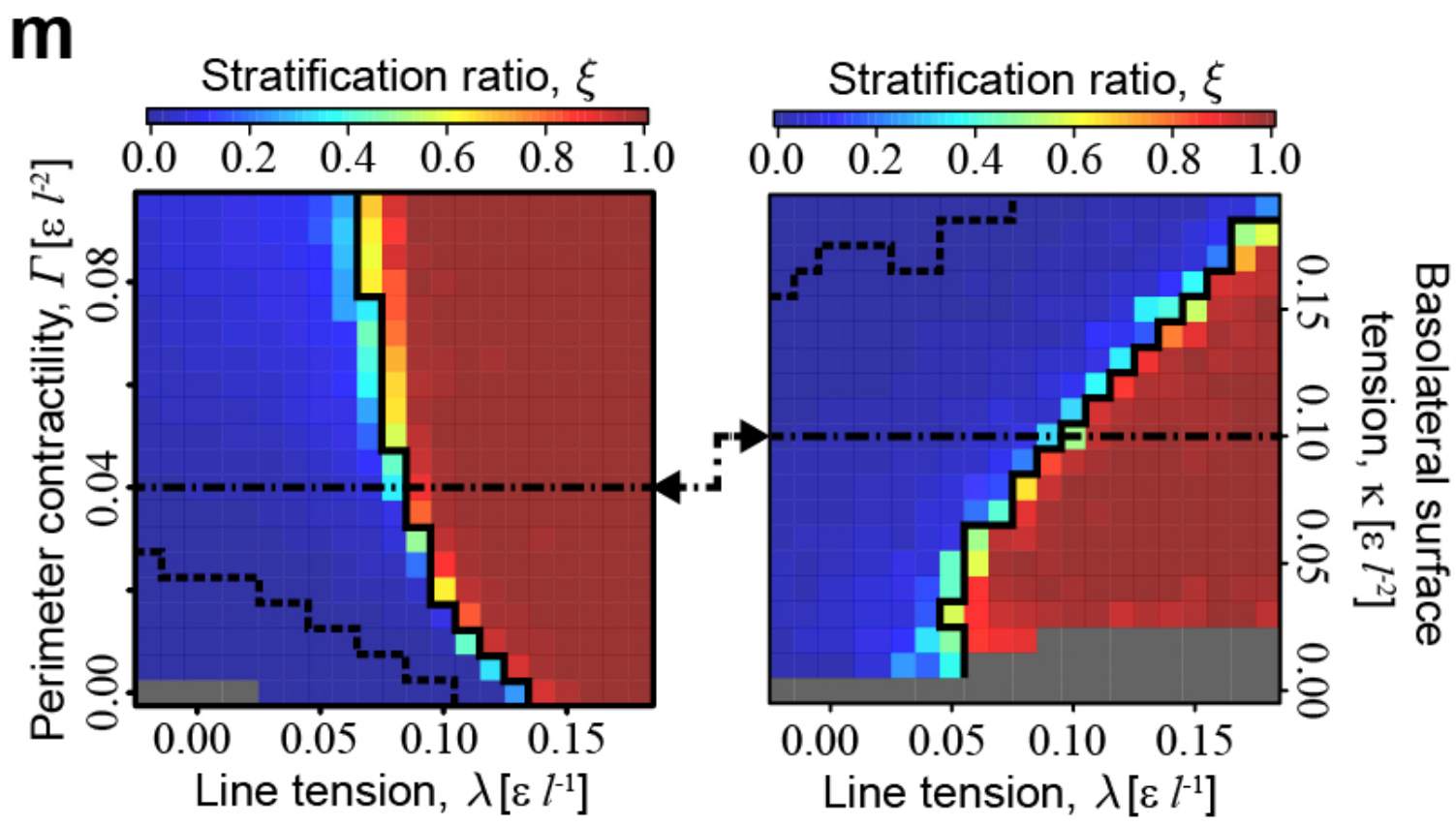

m Stratification ratio, ξ, with respect to Γ and λ (left), with respect to κ and λ (right), respectively. In (m), solid and dotted lines indicate boundaries among the multilayer ($\xi > 0.5$), mixed layer ($0.5 \geq \xi > 0.0$), and complete monolayer ($\xi = 0.0$). The data in (**l**), (**m**), and (**o**) indicate the average values calculated from five independent initial conditions. Gray regions indicate collapsed states.

Reviewer Comment 2-6:

As a general final comment, the analyses based on the 3D vertex model remain largely disconnected from the mean-field model. Indeed, the vertex model is used primarily to reproduce experimental observations for selected parameter sets, rather than to systematically test or quantify the proposed mechanisms. As a result, the full potential of the vertex simulations to provide mechanistic insight is not realized. A tighter integration between the mean-field theory, the vertex-model simulations, and the experimental data would be necessary to substantiate the central claims of the work.

Authors' response 2-6:

We thank the reviewer for this important general comment. We agree that the relationship among the mean-field theory, 3D vertex-model simulations, and experimental data was not sufficiently clear in the original manuscript. As described in our responses to the specific comments above, we have revised the manuscript to better integrate these three components. In particular, we added quantitative vertex-model analyses to test the mechanical-instability interpretation and clarified that the mean-field model identifies possible routes to delamination, whereas the vertex simulations and experiments indicate which route is relevant in MDCK cell culture. These revisions have been incorporated into the revised **Results** and **Methods**.

Reviewer Comment 2-7:
Additional minor concerns:
1) If only apical parameters are varied, does this imply that the model can only capture delamination events associated with loss of the apical side? The authors should clarify how different delamination processes are represented, in particular whether the model can account for cells losing either their apical or basal side.

Authors' response 2-7:
We thank the reviewer for this helpful comment. In the 3D vertex model, delamination is defined geometrically based on cell contact with the apical and basal surfaces. Thus, the model can represent cells losing either apical or basal contact, and delamination is not restricted to loss of the apical side even when apical mechanical parameters are varied. In the present analysis, we grouped both apical-loss and basal-loss events under a single definition of delamination. We have clarified this definition and its implementation in the revised **Results**.

[Results, Page 7]
As in the experiments, we classified cells in the model according to their contacts with the apical and basal surfaces as apicobasal-spanning cells (Fig. 1a). Cells that lacked either the apical surface, the basal surface, or both were defined as delaminated cells.

Reviewer Comment 2-8:
The definition of planar density appears inconsistent throughout the manuscript. I recommend that the authors clarify this quantity explicitly and ensure a consistent definition in both the main text and Methods.

Authors' response 2-8:
We thank the reviewer for pointing this out, and we apologize for the unclear presentation in the original manuscript. We have unified the definition of planar cell density throughout the manuscript as the number of cells per unit projected area. In the experiments and 3D vertex-model simulations, this quantity is calculated directly by dividing the number of cells by the projected area. In the mean-field model, the expression $\rho = T/V$ follows from the same definition under the assumption of a fixed cell volume, because the projected area per cell

is V/T. We have revised the main text and **Methods** to make this definition and its derivation explicit.

[Methods, Page 20]
Planar cell density, ρ, was defined as the number of cells per unit projected area in both experiments and simulations. In MDCK experiments, cells were counted using mCherry-CAAX fluorescence. To account for cells intersecting the image boundary, the number of cells was calculated as $N_c + N_e/2$, where N_c is the number of cells fully contained within an image and N_e is the number of cells intersecting the image boundary. ρ was then calculated by dividing this cell number by the total image area. To analyze the relationship between ξ and ρ, both quantities were calculated for each individual image. In simulations, where periodic boundary conditions were imposed, ρ was calculated as $N_t/(L_x L_y)$, where N_t is the total number of cells and L_x and L_y are the system sizes in the x- and y-directions, respectively.

[Methods, Page 27]
Specifically, we considered an epithelial monolayer of thickness (T), composed of cells with a fixed volume (V_0). Under this assumption, the projected area per cell is V_0/T. Therefore, the planar cell density is expressed as $\rho = T/V_0$, which is consistent with the definition of cell number per unit projected area used in the experiments and simulations.

Reviewer Comment 2-9:
In the main text, the authors state: "we postulated that all cell-cell boundary surfaces comprised parts of the basolateral surfaces, and the other cell surfaces composing the tissue surface were defined as the apical surfaces." This sentence is somewhat confusing and is not further clarified in the Methods section. Greater clarity on this point would help the reader interpret the model assumptions.

Authors' response 2-9:
We apologize for the confusion caused by our previous wording. We have revised the corresponding sentence in the **Results** to clarify the surface definitions used in the model. The epithelial sheet has two free boundary surfaces: the upper free surface is defined as the apical surface, and the lower free surface is defined as the basal surface. Cell–cell interface faces are defined as lateral surfaces.

Throughout the manuscript, we use the term “basolateral surfaces” to refer collectively to the basal and lateral surfaces, that is, all non-apical surfaces.

[Results, Page 6]

In our model, the epithelial sheet has two free surfaces, which we define as the apical and basal surfaces. Specifically, the upper free surface is defined as the apical surface, whereas the lower free surface is defined as the basal surface. Throughout the manuscript, we refer to the cell–cell interfaces and the basal surface collectively as the basolateral surfaces.

Reviewer Comment 2-10:

Some figures are not particularly informative, specifically Figure 1f, the simulation snapshots in Figure 2l, and the schematic in Figure 4e. I suggest that the authors either revise these figures to improve clarity or consider removing them to avoid potential confusion.

Authors’ response 2-10:

We thank the reviewer for this helpful suggestion. We revised the figure set to improve clarity and reduce redundancy. Specifically, we removed **Fig. 1f** and the schematic in **Fig. 4e** from the previous manuscript, moved the simulation snapshots in **Fig. 2l** embedded in a graph, and updated the corresponding figure legends. We also merged **Fig. 1c** with **Fig. 1a** where appropriate to streamline the presentation. These changes are reflected in the revised figures and legends.

RESPONSE TO REVIEWER #3

Reviewer Comment:

The manuscript NCOMMS-25-85464 "Epithelium stratifies via nucleation and growth induced by foam-geometric instability" by Fukamachi et al. reports a comprehensive experimental and theoretical analysis of the transition between single-cell-thick and multilayer epithelial tissues. The authors present experimental results suggesting that stratification can be regarded as nucleation and growth typically associated with, e.g., condensation of gas or crystallization of a liquid. This is an interesting analogy, and the authors provide various pieces of evidence that support it. They also employ a fairly detailed version of the 3D vertex model to simulate stratification, which includes the scutoid shapes. The predictions of the model agree rather nicely with observations. The manuscript is interesting and well-written, the key findings considerably advancing the insight into the mechanics of tissues. The manuscript warrants publication in Nat. Commun. and I suggest that it be accepted after the authors have considered the suggestions listed below.

I only have a few suggestions and questions:

Reviewer Comment 3-1:

In the 3D vertex model, does the tissue remain flat upon stratification? Fig. 2f-h suggest that some deformation takes place. Is this relevant? Does wrinkling present facilitate or suppress stratification?

> **Authors' response 3-1:**
>
> We thank the reviewer for this important observation. To examine whether tissue deformation accompanies stratification in our 3D vertex model, we quantified wrinkling by measuring the standard deviation of apical and basal surface curvature and compared these values with epithelial layer structures. This analysis showed that monolayered tissues generally remained relatively flat, whereas stratified tissues often exhibited increased surface curvature and wrinkling. These results suggest that wrinkling is associated with stratification, although the present analysis does not establish whether wrinkling is a cause or a consequence of multilayer formation. We therefore discuss in the **Discussion** the possibility that local curvature and geometric deformation may influence stratification by altering local mechanical stresses and packing constraints, while

noting that the causal role of curvature remains an important question for future work. The corresponding analysis has been added to **Supplementary Fig. 4a–e**.

[Discussion, Page 15]

Relatedly, monolayer tissues generally remained relatively flat, whereas stratified tissues in our simulations often exhibited increased surface curvature and wrinkling (Supplementary Fig. 4a–e). These observations suggest that collapsed tiling in 2D may represent a planar signature of an instability that can manifest as stratification and tissue-scale deformation in 3D, although future studies will be needed to quantitatively compare these regimes and determine the causal role of curvature.

[Supplementary Fig. 4 & legend]

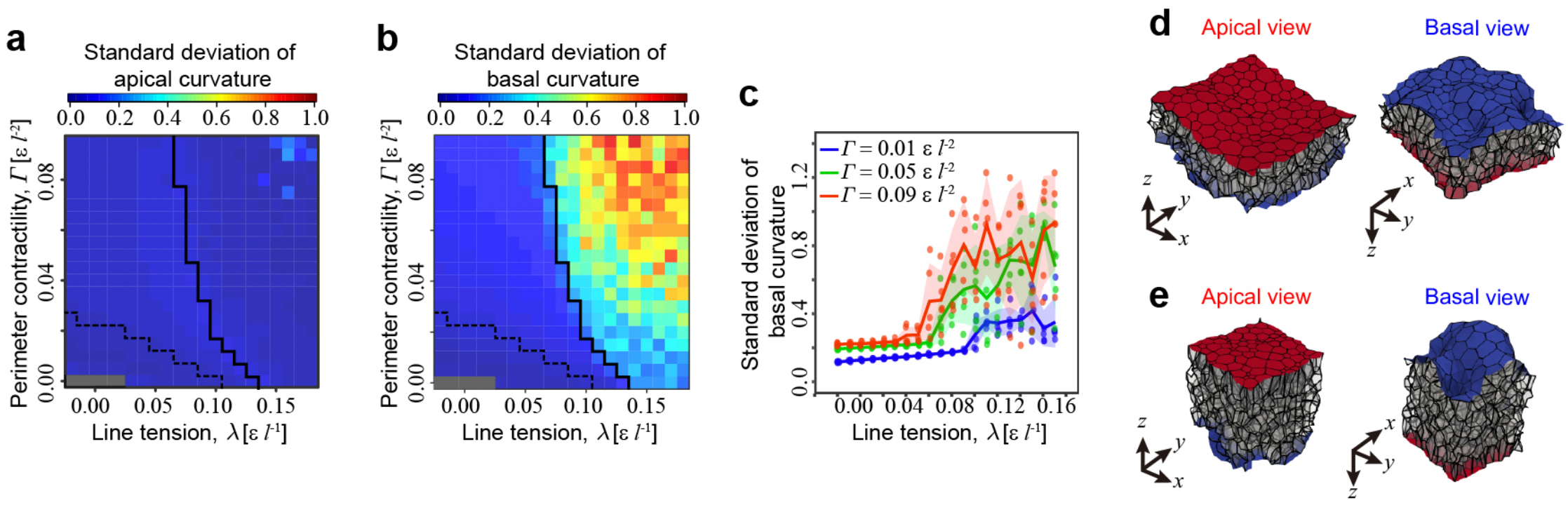

a, **b** Standard deviation of apical curvature, and standard deviation of basal curvature, with respect to apical perimeter contractility, Γ, and apical line tension, λ. **c** standard deviation of basal curvature as a function of λ. **d**, **e** Apical and basal surfaces after relaxation. The left and right panels show the same tissue viewed from apical and basal sides, respectively. Surface colors indicate the apical (red), lateral (gray), and basal (blue) surfaces. The parameters used were set to $\Gamma = 0.02\ \varepsilon l^{-2}$, $\lambda = 0.15\ \varepsilon l^{-1}$ (**d**), $\Gamma = 0.08\ \varepsilon l^{-2}$, $\lambda = 0.15\ \varepsilon l^{-1}$ (**e**). In (**a**) and (**b**), solid and dotted lines indicate boundaries among the multilayer ($\xi > 0.5$), mixed layer ($0.5 \geq \xi > 0.0$), and complete monolayer ($\xi = 0.0$). ssIn (**a**) and (**b**), the gray-colored regions indicate the collapsed state. The data in (**a**) and (**b**) indicate the average values calculated from five independent initial conditions.

Reviewer Comment 3-2:

In the model, the apical elasticity term and the line tension of the apical junctions be

combined in a single term of type ¥Gamma (P-P_0)^2 where P_0 is a preferred cell apical perimeter. It seems that P_0 used in the manuscript is negative so that the two terms would prefer a collapsed apical tiling (cf. Ref. [46], Farhadifar et al. 2007). If I compare Figs. 2i and especially 2j to Fig. 1 from Ref. [46] flipped across the diagonal and somewhat shifted up, it seems to me that the notion of the collapsed tiling from Ref. [46] may correspond to stratification. Is it so? If yes, would it make sense to discuss it ? Evidently, stratification in a 3D vertex model of a tissue is much more complex but if the collapse of the 2D vertex model can be viewed as an indication of stratification this so far unphysical domain would no longer be unphysical. If the collapse of the 2D vertex model indeed signals stratification, why the shift in the (contractility, tension) plane mentioned above?

Authors' response 3-2:

We thank the reviewer for this insightful comment. Following the reviewer's suggestion, we added a discussion on the possible relationship between the collapsed-tiling regime in 2D vertex models and stratification in our 3D vertex model. We agree that these two phenomena may be conceptually related: parameter regimes that destabilize apical packing in 2D can, in 3D, be resolved not only by in-plane rearrangements but also by apicobasal deformation and vertical cell rearrangements, leading to delamination and stratification. However, because our 3D model includes additional geometric degrees of freedom, free apical and basal surfaces, and basolateral surface energy, we do not interpret the correspondence quantitatively or expect a direct mapping of the parameter planes. As additional data for the reviewer, we also generated a figure corresponding to Ref. [46] and calculated the distributions of the number of sides and areas of cell apical faces, although these data are not included in the manuscript. We have added this discussion to the **Discussion**.

[Additional data for the reviewer]

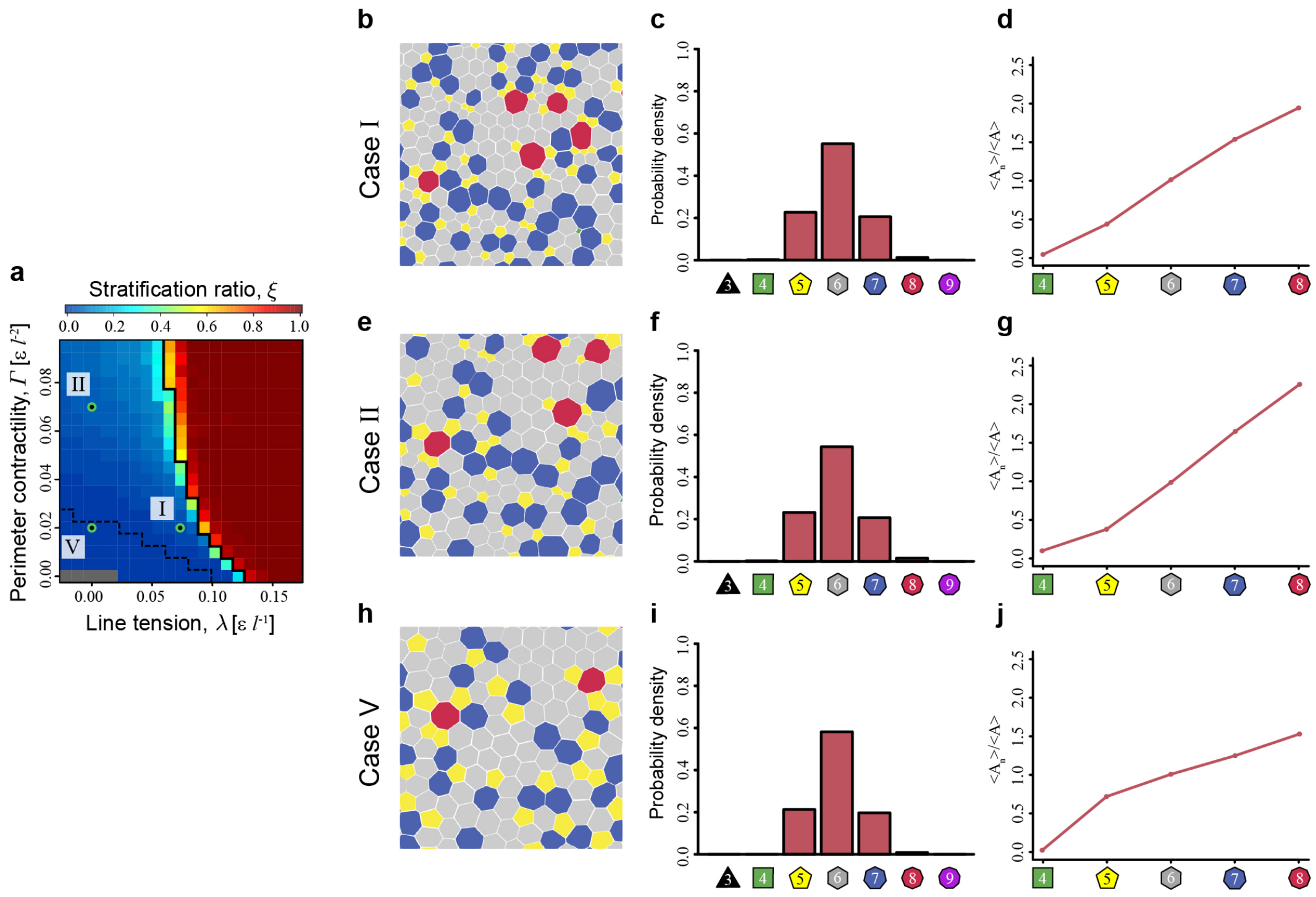

a Stratification ratio, ξ, with respect to Γ and λ (shown in **Fig. 1m**). Green dots indicate the parameter sets corresponding to three representative cases, like those shown in Figure 1(Farhadifar et al. 2007). **b**, **e**, and **h** Examples of network pattern in apical surface for cases I, II, and V. Color indicates number of neighboring cells. **c**, **f**, and **i** Probability density of neighbor numbers P_n for cases I, II, and V. **d**, **g**, and **j** Average areas of different polygon classes normalized to the average area of cells in the network, $\langle A_n \rangle / \langle A \rangle$, for cases I, II, and V.

[Discussion, Page 15]

The stratified regime in our 3D model may be conceptually related to the collapsed-tiling regime described in 2D vertex models[46]. In 2D, apical packing instability can only be resolved within the epithelial plane, whereas in 3D cells can also deform and rearrange along the apicobasal axis, leading to delamination and stratification. Therefore, although the two regimes appear qualitatively similar, they occupy different regions in the λ and Γ parameter space. This additional geometric freedom may explain why the corresponding regimes are not quantitatively aligned in parameter space.

Reviewer Comment 3-3:

Presentation: All figures are quite elaborate and contain many panels. IMHO some panels may be redundant, e.g., Fig. 2j which conveys a very similar message as Fig. 2i. While all graphical material included is relevant, I suggest that the authors consider moving some of it to Supplementary Information so as to better emphasize the essential bits and to reduce reiterating the same message.

Authors' response 3-3:

We thank the reviewer for this helpful suggestion. We revised the figures to reduce redundancy and better emphasize the essential results. Specifically, we merged the previous Fig. 1c into Fig. 1a, removed redundant panels from the previous Figs. 1, 2, and 4, and moved several supporting panels from the main figures to the Supplementary Information, including the previous Fig. 1e, Fig. 3d–g, and Fig. 4g–i. We updated the corresponding figure numbering, legends, and text accordingly.

Reviewer Comment 3-4:

Secondly, here and there the language can be improved (e.g., "across broad phenomena" in l. 26 is awkward, "unordered" -> "disordered" in l. 32 and 68). In Figs. 2c, 2i, 2j, 3e, 3f, and 3g, "Perimeter contraction" -> "Perimeter contractility".

Authors' response 3-4:

We thank the reviewer for pointing these out. We improved the language throughout the manuscript, including replacing "across broad phenomena" with "across a wide range of phenomena" and "unordered" with "disordered," and corrected typographical errors in the figure labels.
