# Supplementary material for: Epithelium stratifies via nucleation and growth induced by foam-geometric instability
Source: Nat Commun. 2026 Nov 13;17:11418. doi: 10.1038/s41467-026-78964-1 (PMC13768340; doi:10.1038/s41467-026-78964-1)
Supplement: Supplementary file 4 [file 41467_2026_78964_MOESM4_ESM.zip › SourceData2.zip!/README.rtf]

This dataset contains VTK files of the simulation results shown in Fig. 1m. The simulation parameters and time are encoded in the file and folder names.
File naming convention
Example 1:
LambdaGamma/lambda0.07_gamma0.060_octv000000010000000.vtk
•	lambda0.07: apical line tension coefficient (ë = 0.07)
•	gamma0.060: apical perimeter contraction coefficient (Ã = 0.060)
•	octv000000010000000: simulation time (ô = 1000)
Example 2:
LambdaKappa /lambda0.17_kappa0.180_octv000000010000000.vtk
•	lambda0.17: apical line tension coefficient (ë = 0.17)
•	kappa0.180: basolateral surface tension coefficient (ê = 0.180)
•	octv000000010000000: simulation time (ô = 1000)
The simulation time ô is obtained by dividing the numerical value following octv by 10,000.
